# Gene Expression Profile and Toxic Effects in Human Bronchial Epithelial Cells Exposed to Zearalenone

**DOI:** 10.1371/journal.pone.0096404

**Published:** 2014-05-02

**Authors:** Mei Yu So, ZhiPeng Tian, Yong Shian Phoon, Sha Sha, Michael N. Antoniou, JiangWen Zhang, Rudolf S. S. Wu, Kian C Tan-Un

**Affiliations:** 1 School of Biological Sciences, The University of Hong Kong, Hong Kong SAR, China; 2 School of Professional and Continuing Education, The University of Hong Kong, Hong Kong SAR, China; 3 Department of Medical and Molecular Genetics, Gene Expression and Therapy Group, King's College London School of Medicine, Guy's Hospital, London, United Kingdom; Univesity of Texas Southwestern Medical Center at Dallas, United States of America

## Abstract

Zearalenone (ZEA), a mycoestrogen produced by *Fusarium* fungal species, is mainly found in cereal crops such as maize, wheat and barley. Although ZEA has been reported to be present in air, little is known about the health risk or the molecular basis of action when lung cells are exposed to ZEA. As ZEA has a similar structure to estrogen, its potential risk as an endocrine disrupting chemical (EDC) has thus aroused both environmental and public health concerns. The purpose of this study is to identify the responses and underlying molecular changes that occur when human bronchial epithelial BEAS-2B cells are exposed to ZEA. Differential gene expression profiles were identified in cells that were treated with 40 µM ZEA for 6 h and 24 h by high-throughput microarray analysis using Affymetrix Human Gene 2.0 GeneChip. The array results showed that after ZEA treatment, 262 genes at 6 h and 1073 genes at 24 h were invovled in the differential regulation. Pathway analysis revealed that diverse cellular processes were affected when lung cells were exposed to ZEA resulting in impaired response to DNA damage, cell cycle arrest, down-regulation of inflammatory responses and alterations of epigenetic marks. Results of further experiments indicated that 40 µM ZEA decreased cell viability, induced apoptosis and promoted reactive oxygen species (ROS) generation in a time-dependent manner. Immuno-suppressive effects of ZEA were further revealed through the suppression of lipopolysaccharide (LPS)-induced expression of pro-inflammatory cytokines (IL-6, IL-8 and IL-1β). Interestingly, the level of global DNA methylation was markedly decreased after 24 h exposure to ZEA. Collectively, these observations suggested that a broad range of toxic effects are elicited by ZEA. Particularly, ROS may play a pivotal role in ZEA-induced cell death. These adverse effects observed in lung cells suggest that exposure to ZEA may increase susceptibility of lung cells to diseases and required further investigations.

## Introduction

Mycotoxin Zearalenone (ZEA) is a secondary metabolite produced by various *Fusarium* fungal species [1], [2] which are usually found in contaminated maize, wheat and barley [3]. Due to its structural similarity to estrogen, ZEA competes with estradiol for binding to estrogen receptors (ERs) and provokes estrogenic activities. Extensive studies have found that ZEA caused endocrine disruption and reproductive disorders in *in vitro* models and in laboratory and farm animals [4], [5], [6], [7]. In addition, other effects of ZEA including developmental toxicity, immunotoxicity and genotoxicity have also been reported [3]. Increasing evidence suggested that these effects are not exclusively due to the estrogenic potency of ZEA but that oxidative stress may be an important mediator of these observed toxic effects [8], [9], [10].

Besides foods and feeds, inhalation is another route of exposure to ZEA. The detection of ZEA-producing fungi and toxigenic spores in nasal cavity has been reported [11], [12]. In addition, the detection of air-borne ZEA was also documented. In a Belgium study, the maximum level of ZEA detected was 2.4 µg/kg ZEA which meant that exposure through dust inhalation for workers in those companies was estimated to be 0.1% of the tolerable daily intake of ZEA [13]. In a study carried out in Dalian, China, it was reported that the daily inhaled ZEA by a worker in a poultry house was estimated to be 17.432–20.512 ng respectively [14].

Estrogens have been shown to induce proliferation of non-small cell lung cancer (NSCLC) through ER-mediated signaling pathways [15]. Additionally, estrogen is also involved in the activation of carcinogens via the metabolism of polycyclic aromatic hydrocarbonds (PAHs) which promotes the formation of catechol estrogens and potentially mutagenic DNA adducts [16], [17]. Interestingly, large cohort epidemiological studies indicate that females are more susceptible to developing chronic lung diseases including asthma and Chronic Obstructive Pulmonary Disease (COPD) [18].

To date, the molecular basis of the effects of ZEA in lung cells has not been fully investigated. Using a toxigenomic approach, we attempted to study the mechanism of actions of ZEA on lung cells. In addition, we show that ZEA induces a broad range of toxic effect not solely because of its estrogenic potency but also through induction of oxidative stress. A BEAS-2B cell line over-expressing a free radical scavenger, cytoglobin (CYGB) confirm that ZEA generates free radicals.

## Materials and Methods

### Cell culture and treatments

Human bronchial epithelial BEAS-2B cell line [19] (from the American Type Culture Collection, ATCC) was cultured in Dulbecco's Modified Eagles Medium (DMEM) supplemented with 10% (v/v) fetal bovine serum (FBS) and 100 U/ml of penicillin and 10 µg/ml of streptomycin. All cells were maintained in a 37°C humidified incubator with 5% CO_2_. DMEM with Geneticin (G418, 200 µg/ml) were used to maintain and select Cygb overexpressing cells.

ZEA powder (Sigma) was dissolved in DMSO, aliquoted and stored at −20°C. The stock solution of ZEA was freshly diluted by culture medium before use. BEAS-2B cells were seeded overnight to achieve confluency. Cells were exposed to different concentration of ZEA or 0.05% DMSO solvent control for different durations (6, 12, 24 and 48 h) depend on experiments. For immune-responsive experiments, cells were stimulated by 2 µg/ml lipopolysaccharide (LPS, Sigma) for 6 h.

### Cell viability assay

1×10^4^–1×10^5^ cells were seeded in 96-well plates. After incubation overnight, cells were exposed to a serial concentration of ZEA (from 0 to 160 uM) for 6 h, 24 h or 48 h. 20 µl of 3-(4,5-dimethylthiazol- 2-yl)-2,5-diphenyltetrazolium bromide (MTT, Invitrogen) solution was then added to each well. After 3 hours incubation, the medium was removed and DMSO was added to dissolve the purple formazan. The optical density of each well was quantified by measuring absorbance at 540 nm. The viabilities of treated groups were expressed as a percentage of control group, which was assumed as 100%.

### Sample preparation and RNA extraction

5×10^5^ cells were seeded onto 6-well plates. After incubation overnight, cells were treated with either 0.05% DMSO (solvent control) or 40 µM ZEA (treatment) for 6 h and 24 h. RNA was extracted by Trizol reagent (Invitrogen) according to the manufacturer's protocol. The quality of RNA for microarray analysis was analyzed by the RNA 6000 Nano total RNA Assay using the Agilent 2100 Bioanalyzer. Only RNA samples with an A_260_/A_280_ ratio ∼1.8, 28S/18S ratio larger than 1.8 and RNA integrity number (RIN) larger than 8.0 were used for downstream GeneChip analysis.

### Gene expression profiling and data analysis

The gene expression profiles were determined using GeneChip® Human Gene 2.0 ST Arrays (Affymetrix). Samples were hybridized onto array chips, stained, washed, and scanned according to Affymetrix protocol. The array image and cell intensity files (.CEL files) were generated by Affymetric GeneChip Command Console. Both the RNA quality control tests and GeneChip analysis were conducted by Center for Genomic Services, HKU.

The data of GeneChip were analyzed using Agilent GeneSpring GX11 Software, Affrymetrix Transcriptome Analysis Console (TAC) Software and R software (http://www.r-project.org). When using the GeneSpring Software, filtering was performed to remove background noise. Probes that have signals weaker than 20^th^ percentile of overall signal were not included into analysis. Differentially expressed genes were identified as fold change ≥1.5 and p<0.05 by unpaired t-test. Over-represented Gene Ontology (GO) terms and enriched pathways associated with the list of differentially expressed genes were generated by the built-in GO and Single Experiment Analysis (SEA) of GeneSpring. Only GO terms and pathways that have more than 2 entities (≧3) involved and p-value <0.05 were considered.

To further analyze the gene expression data, the Gene Set Enrichment Analysis (GSEA) were used to identify cohorts of genes which are linked to certain biological processes/cellular signaling pathways. Differentially expressed genes with annotations and known features were subjected to GSEA. Pathways are ranked according to the significance of enrichment [61]. Gene sets with a p-value <0.05 by one-way ANOVA and False Discovery Rate (FDR) <0.05 by multiple test correction of were considered to be significantly affected.

### Validation of gene expression by Quantitative Real-time PCR (qPCR)

cDNA was synthesized from 1 µg RNA by using PrimeScript™ RT Master Mix (Takara) according to manufacturer's instructions. Primers were designed to avoid amplification of genomic DNA using Primer Premier 5 software. The sequences of the primers used are shown in Table S1. qPCR was performed using FastStart Universal SYBR Green Master mix (Roche) and reaction mixes were set up as per manufacturer's instructions. The cycles were set as 95°C, 10 min, 40 cycles of 95°Cfor 15 sec, 60°Cfor 20 sec and 72°Cfor 45 sec followed by melting curve analysis. The change in gene expression was calculated by comparative C_T_ method with the housekeeping gene β–actin used for normalization.

### Measurement of Reactive Oxygen Species (ROS) levels by DCFH-DA assay

The intracellular ROS levels were quantified by a fluorescent probe, 2′,7′-dichlorofluorescein-diacetate (DCFH-DA, Molecular Probes). After treatment with 40 µM ZEA, cells were washed and resuspended in PBS at a concentration of 10^6^ cells/ml and then incubated with 10 µM DCFH-DA at 37°C for 40 minutes in the dark. The ROS production was quantified by DCF fluorescence intensity from 10^4^ cells by flow cytometry. Results were expressed as the percentage of ROS generation as compared to control.

### Establishment of *Cygb* stably overexpressing cells

The stable BEAS-2B over-expressing Cytoglobin (*Cygb*) by comprising the coding region of mouse *Cygb* which was cloned to pcDNA 3.1/V5-His A vector (Invitrogen) between Hind III and Xho I sites.

To establish stably *Cygb* expressing cells, a 1576 bp core ubiquitously-acting chromatin opening element (UCOE, a gift from Dr. Michael Antoniou, School of Medicine, King's College London, UK) was inserted to the upstream of the CMV promoter of pcDNA 3.1/V5-His A/Cygb. Successfully transfected cells were selected using selective medium (DMEM with 10% FBS and 600 µg/ml G418). After 14 days of selection, single colonies were picked and expression levels were checked to identify the clones with *Cygb* over-expression. The incorporation of the UCOE onto the *Cygb*–transgene construct resulted in the sustained high expression of cytogobin in the stable BEAS-2B cell line. This stable over-expression cell line was employed as a model system to investigate the effects of free radicals generated by ZEA on cell viability.

### Cell death pathway analysis by Annexin V/PI double staining

The apoptotic statuses of cells were determined using Annexin V-FITC Apoptosis Detection Kit I (BD Pharmingen) following manufacturer's instructions. Briefly, cells treated with 40 µM ZEA were washed with phosphate buffered saline (PBS) and then resuspended in binding buffer at a concentration of 10^6^ cells/ml. Annexin V-FITC and Propidium Iodide (PI) were added to the resuspended cells. Cells were incubated for 15 min at room temperature in the dark. Apoptotic cells were analysed from 10^4^ cells with a Beckman-Coulter FACScan flow cytometer. The percentage of living, early apoptotic and dead cells were quantified using the Modfit LT program.

### Global DNA methylation analysis

To obtain an insight into the epigenetic effects of ZEA, global DNA methylation levels were investigated. 5-aza-cytidine, a known DNA methylation inhibitor acting as a substitute substrate for DNA methyltransferase, was used as positive control. BEAS-2B cells were treated with DMSO, 40 µM ZEA or 1 µM 5-aza-cytidine for 24 h. After treatment, genomic DNA was extracted using UltraClean Tissue & Cells DNA Isolation Kit (MO Bio Laboratories, Inc.) according to the manufacturer's protocols. The concentrations and qualities of DNA were quantified by NanoDrop ND-1000 Spectrophotometer (Nano-Drop Technologies) and checked by 0.7% agarose gel electrophoresis, respectively. The global DNA methylation levels were determined using MethylFlash Methylated DNA Quantification Kit (Colorimetric) (Epigentek Group Inc.) following manufacturer's instructions. DNA is bound to specifically treated strip wells that have high DNA affinity. The 5-methylcytosine of DNA is detected using antibodies and quantified using an ELISA-like reaction by reading absorbance at 450 nm.

### Statistical analysis

Unless otherwise specified, all data are results of 3 independent experiments, each with 3 samples per group and represent as mean± SD. Student t-test and one-way analysis of variance (ANOVA) followed by Duncan's post hoc test were conducted using SPSS v11.5 software. Values of *p*<0.05 were considered as significant.

## Results

### ZEA reduced viability of BEAS-2B cells

**Figure 1:**
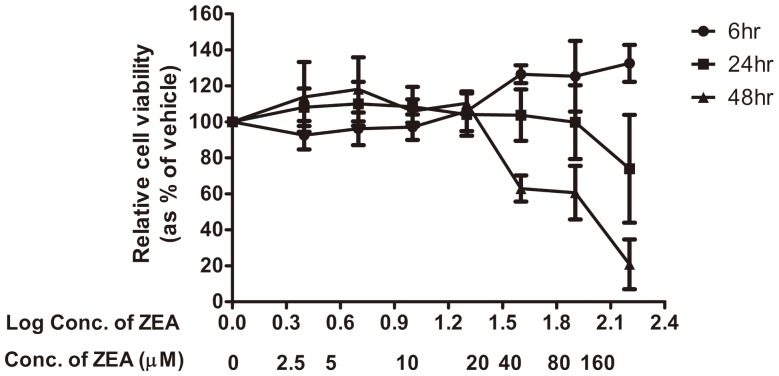
Cytotoxic effects of ZEA on BEAS-2B cells determined by MTT assay. Cells were treated with increasing concentrations of ZEA (from 0 to 160 µM) for 48 h. Cell viability was expressed as percentages of control (values taken as 100%) and are mean ± SD of at least 3 independent experiments. * represents *p*<0.05 significantly different from control as assessed by t-test.

The cytotoxic effects of ZEA on cell viability were determined using MTT assay. The results revealed that ZEA induced cell death in a time- and dose-dependent manner (*p*<0.05). After 48 h treatments, the Lowest Observable Adverse Effect Level (LOAEL) was 40 µM, with a percentage of viable cells of 62.96±7.30% (Figure 1). As 40 µM has no significant inhibitory effect on cell viability after 6 h and 24 h treatments but was the LOAEL after 48 h treatment, it was chosen as the concentration for use in subsequent experiments.

### Identification of differentially expressed genes

**Figure 2:**
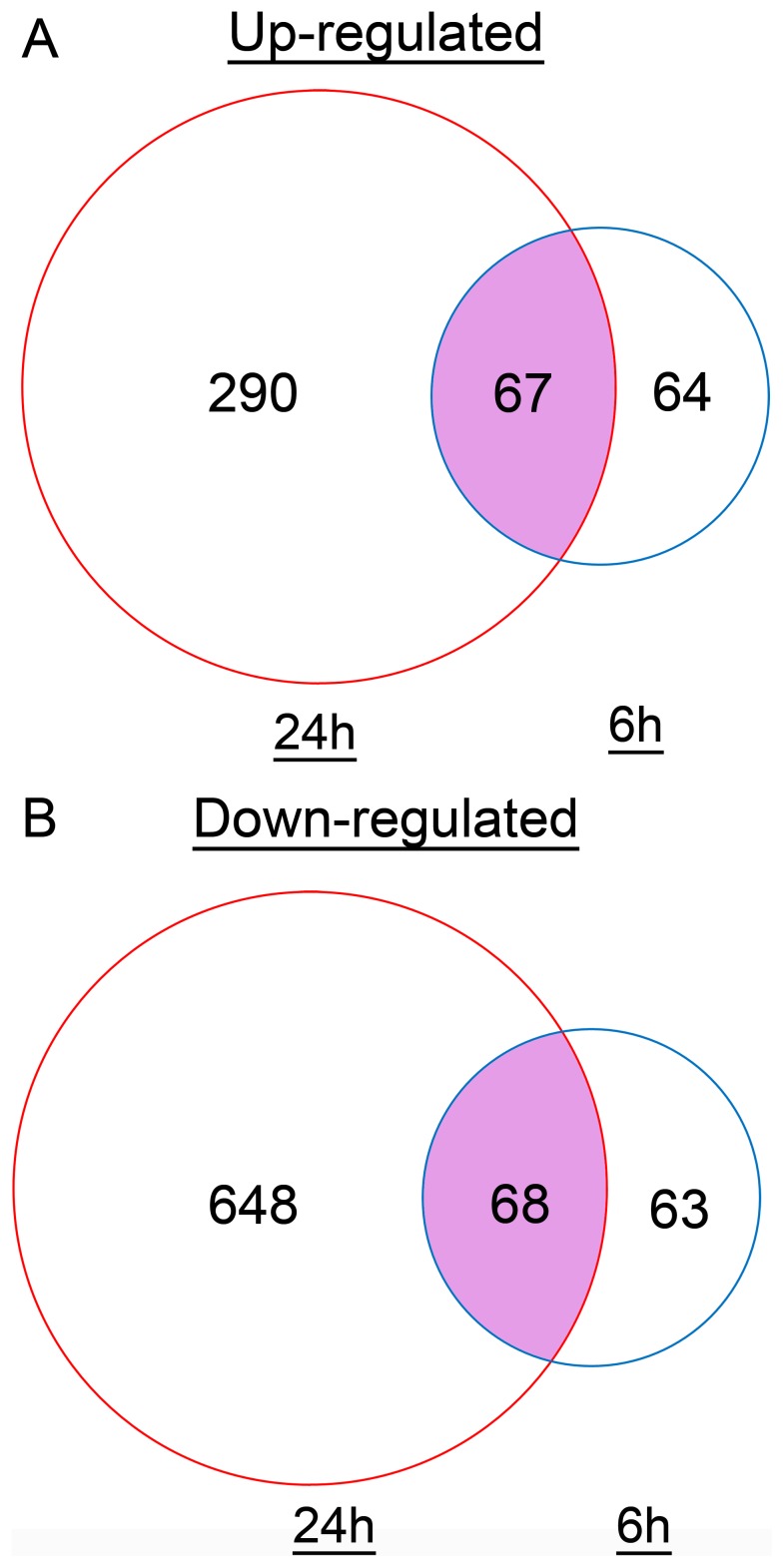
Venn diagrams of differential expressed genes at different time points after ZEA treatment. The blue circles represent 6(A) Up-regulated genes. (B) Down-regulated genes.

The number of differentially expressed genes are observed to increase in a time-dependent manner. According to the Transcriptome Analysis Console (TAC) software, out of 53,617 gene probes on the Genechip, 262 (131 genes up-regulated and 131 genes down-regulated) and 1073 (357 genes up-regulated and 716 genes down-regulated) genes were differentially expressed at 6 h and 24 h, respectively. The complete list of the differentially expressed genes and their fold change at 6 h and 24 h are shown in Table S2 and S3, respectively. The number of commonly up-regulated and down-regulated genes at both time points is 67 and 68, respectively (Figure 2).

For both time-point, the most significantly up-regulated genes are heat shock proteins of 70 kDA in size (HSPA1B, HSPA1A) which increased by 3.7-fold and 6.54-fold at 6 h and 24 h, respectively (Table 1 and Table 2). On the other hand, the most significantly down-regulated gene common for both time points is serpin peptidase inhibitor, clade B (ovalbumin), member 2 and 10 (SERPINB2, B10) which decreased by 2.87-fold and 5.52-fold at 6 h and 24 h, respectively.

**Table 1:** The 30 most differentially expressed genes in BEAS-2B cells after 6 h treatment with ZEA.

Gene symbol	Gene description	Fold change	p-value
			
***Up-regulated:***			
HSPA1B, HSPA1A	Heat shock 70 kDa protein 1B, heat shock 70 kDa protein 1A	3.78	7.87E-07
SDIM1	Stress responsive DNAJB4 interacting membrane protein 1	2.63	0.00041
COPG2IT1	COPG2 imprinted transcript 1 (non-protein coding)	2.56	0.00087
DNAJA4	DnaJ (Hsp40) homolog, subfamily A, member 4	2.29	8.2E-05
LINC00473	Long intergenic non-protein coding RNA 473	2.29	6.4E-05
PARD6G-AS1	PARD6G antisense RNA 1 (non-protein coding)	2.25	7.2E-05
FAM72C	Family with sequence similarity 72, member C	2.22	0.0003
HSPA4L	Heat shock 70 kDa protein 4-like	2.17	1.7E-05
DDIT4	DNA-damage-inducible transcript 4	2.15	0.00044
ARL17A, ARL17B	ADP-ribosylation factor-like 17A, ADP-ribosylation factor-like 17B, ADP-ribosylation factor-like protein 17-like	2.13	9.25E-07
BAG3	BCL2-associated athanogene 3	2.12	2.1E-05
MT1F	Metallothionein 1F	2.05	0.00039
DRP2	Dystrophin related protein 2	2.03	0.00581
CHORDC1	Cysteine and histidine-rich domain (CHORD) containing 1	2.02	0.00012
LINC00310	Long intergenic non-protein coding RNA 310	1.98	6.9E-05
***Down-regulated:***			
KRTAP2-4	Keratin associated protein 2-4-like, keratin associated protein 2-4	−3.28	7.7E-05
SERPINB2, SERPINB10	Serpin peptidase inhibitor, clade B (ovalbumin), member 2, serpin peptidase Inhibitor, clade B (ovalbumin), member 10	−2.87	4E-06
PLAU	Plasminogen activator, urokinase	−2.79	4.2E-05
SHISA2	Shisa homolog 2 (Xenopus laevis)	−2.72	0.00001
CYP1B1	Cytochrome P450, family 1, subfamily B, polypeptide 1	−2.66	7E-06
DLX2	Distal-less homeobox 2	−2.63	0.00007
EDN1	Endothelin 1	−2.57	6.9E-05
FOSL1	FOS-like antigen 1	−2.44	1.1E-05
ADAMTS1	ADAM metallopeptidase with thrombospondin type 1 motif, 1	−2.29	0.00062
SMAD7	SMAD family member 7	−2.24	0.00039
KLF10	Kruppel-like factor 10	−2.22	0.00018
IL8	Interleukin 8	−2.22	0.0107
KRT80	Keratin 80	−2.11	0.00016
RUNX2	Runt-related transcription factor 2	−2.05	2.6E-05
LOC100131234	Familial acute myelogenous leukemia related factor	−2.04	8.4E-05

**Table 2:** The 30 most differentially expressed genes in BEAS-2B cells after 24 h treatment with ZEA.

Gene symbol	Gene name	Fold change	p-value
			
***Up-regulated:***			
HSPA1B, HSPA1A	Heat shock 70 kDa protein 1B, heat shock 70 kDa protein 1A	6.54	3.46E-08
NR4A3	Nuclear receptor subfamily 4, group A, member 3	5.79	0.00018
CLDN12, CDK14	Claudin 12, cyclin-dependent kinase 14	5.25	0.00074
AGBL5-AS1	AGBL5 antisense RNA 1 (non-protein coding)	4.11	0.0001
LMOD1	Leiomodin 1 (smooth muscle)	3.91	4.9E-05
DNAJA4	DnaJ (Hsp40) homolog, subfamily A, member 4	3.32	4.5E-05
CHORDC1	Cysteine and histidine-rich domain (CHORD) containing 1	2.96	2.9E-05
LRP4-AS1	LRP4 antisense RNA 1 (non-protein coding)	2.95	0.00208
SDIM1	Stress responsive DNAJB4 interacting membrane protein 1	2.79	0.00018
HSPA4L	Heat shock 70 kDa protein 4-like	2.75	1.3E-05
HSPH1	Heat shock 105 kDa/110 kDa protein 1	2.66	6.3E-05
DDIT4	DNA-damage-inducible transcript 4	2.62	1.7E-05
ALDH1L2	Aldehyde dehydrogenase 1 family, member L2	2.55	0.00011
SNORD14E	Small nucleolar RNA, C/D box 14E	2.54	0.00154
CCDC146	Coiled-coil domain containing 146	2.52	0.0004
			
***Down-regulated:***			
HLF	Hepatic leukemia factor	−6.4	1.8E-05
SERPINB2, SERPINB10	Serpin peptidase inhibitor, clade B (ovalbumin), member 2, serpin Peptidase inhibitor, clade B (ovalbumin), member 10	−5.52	0.00067
DIO2	Deiodinase, iodothyronine, type II	−5.45	1.2E-05
PALMD	Palmdelphin	−4	0.00019
F2RL2	Coagulation factor II (thrombin) receptor-like 2	−3.92	6.9E-05
MIRLET7A2	MicroRNA let-7a-2	−3.81	0.02093
SHISA2	Shisa homolog 2 (Xenopus laevis)	−3.78	8.5E-05
PSG5	Pregnancy specific beta-1-glycoprotein 5	−3.75	0.00002
TACSTD2	Tumor-associated calcium signal transducer 2	−3.65	5.4E-05
SNORD116-28, SNORD115-26, SNORD115-13, SNORD115-7, SNORD107	Small nucleolar RNA, C/D box 116-28, small nucleolar RNA, C/D box 115-26, small nucleolar RNA, C/D box 115-13, small nucleolar RNA, C/D box 115-7, small nucleolar RNA, C/D box 107	−3.32	0.00459
ADAMTS1	ADAM metallopeptidase with thrombospondin type 1 motif, 1	−3.32	0.00096
CPA4	Carboxypeptidase A4	−3.23	0.00001
MIRLET7C	microRNA let-7c	−3.22	0.0225
FBXO32	F-box protein 32	−3.06	0.00046
EPGN	Epithelial mitogen homolog (mouse)	−3.06	0.00547

### Functional classification of differentially expressed genes

The identification of enriched “Biological Processes” under GO category was performed using GeneSpring. The enriched GO terms at 6 h and 24 h are shown in Table S4 and Table S5 respectively. The most significantly enriched GO term at 6 h is “protein folding” (p = 5.59E-07) while that at 24 h is “DNA dependent DNA replication” (p = 1.35E-14).

### Pathway analysis of differentially expressed genes

The pathways altered after ZEA treatments were identified by single experiment analysis (SEA) using GeneSpring. The over-represented pathways at 6 h and 24 h are shown in Table 3. At 6 h, only the “transforming growth factor-beta (TGF-β) signaling” pathway is altered. At 24 h, the top five most significantly altered pathways are “DNA Replication”, “G1 to S cell cycle control”, “cell cycle”, “synthesis of DNA” and “cholesterol biosynthesis”. These results suggest that ZEA alters DNA replication and cell cycle progression in BEAS-2B cells. The differentially expressed genes associated with the progression of cell cycle from G1 to S phase, the replication, damage and repair of DNA and the apoptotic pathway are shown in Table 4, Table 5 and Table 6 respectively.

**Table 3:** Key pathways predicted by Single Experiment Analysis (SEA) following treatment with ZEA in BEAS-2B cells.

Pathway	Number of differential entities involved	Total number of entities in the category	p-values
***Treated with ZEA for 6 hr:***			
TGF-beta signaling pathway	3	55	0.00499
***Treated with ZEA for 24 hr:***			
DNA Replication	14	42	0
G1 to S cell cycle control	17	68	1.21E-10
Cell cycle	19	103	2.33E-10
Synthesis of DNA	7	13	1.01E-09
Cholesterol biosynthesis	7	17	2.26E-08
Regulation of DNA replication	5	7	6.95E-08
SREBP signaling	10	56	1.45E-07
TGF Beta Signaling Pathway	9	55	1.55E-06
miRNA regulation of DNA Damage Response	10	98	2.64E-05
Lymphocyte TarBase	22	420	4.97E-05
Epithelium TarBase	17	278	5.52E-05
Senescence and Autophagy	10	106	6.23E-05
DNA damage response	8	68	7.07E-05
E2F-MIRHG1 feedback-loop	3	5	8.12E-05
Mitotic M-M-G1 phases	4	15	1.93E-04
AhR pathway	5	28	2.28E-04
SREBF and miR33 in cholesterol and lipid homeostasis	4	18	4.13E-04
APC-C-mediated degradation of cell cycle proteins	3	10	9.03E-04
L1CAM interactions	4	27	0.0011
BMP signaling and regulation	3	12	0.0016
Androgen receptor signaling pathway	7	85	0.0018
Unfolded Protein Response	3	14	0.0021
Leukocyte TarBase	8	128	0.0043
p38 MAPK Signaling Pathway	4	34	0.0048
MAPK signaling pathway	9	161	0.0056
TSH signaling pathway	5	65	0.010
Complement and Coagulation Cascades	4	64	0.020
Mitotic G1-G1-S phases	2	11	0.020
Metabolism of nucleotides	2	12	0.024
Apoptosis	5	83	0.027
Integrin cell surface interactions	2	16	0.032
Interleukin-1 signaling	2	15	0.032
Keap1-Nrf2 Pathway	2	14	0.032
Cancer prevention	2	15	0.036
Cell Cycle Checkpoints	2	16	0.036
miRNAs involved in DDR	4	69	0.043
Interleukin-11 Signaling Pathway	3	40	0.047

**Table 4:** Differentially expressed genes related to cell cycle regulation.

Gene symbol	Gene description	Fold change*	
		6 h	24 h
ANAPC16	Anaphase promoting complex subunit 16		1.6
ASNS	Asparagine synthetase (glutamine-hydrolyzing)		2.35
ATF3	Activating transcription factor 3		−1.63
AURKA	Aurora kinase A		1.62
CCNB1	Cyclin B1		1.6
CCNE1	Cyclin E1	−1.33	−2.11
CCNE2	Cyclin E2	−1.32	−2.93
CD24	CD24 molecule		−2.18
CDC20	Cell division cycle 20 homolog (S. Cerevisiae)		−2.25
CDC45	Cell division cycle 45 homolog (S. Cerevisiae)	−1.26	−1.87
CDC6	Cell division cycle 6 homolog (S. Cerevisiae)		−1.69
CDCA3	Cell division cycle associated 3		1.72
CDCA7	Cell division cycle associated 7		−1.88
CDK14	Cyclin-dependent kinase 14	−1.66	5.25
CDKN2B	Cyclin-dependent kinase inhibitor 2B (p15, inhibits CDK4)		−1.52
CKAP5	Cytoskeleton associated protein 5		1.55
CKS2	CDC28 protein kinase regulatory subunit 2	1.19	1.57
CTGF	Connective tissue growth factor		1.51
DBF4B	DBF4 homolog B (S. Cerevisiae)		1.51
DLGAP5	Discs, large (Drosophila) homolog-associated protein 5		1.52
DSN1	DSN1, MIND kinetochore complex component, homolog (S. Cerevisiae)		−1.65
E2F1	E2F transcription factor 1		−1.61
E2F7	E2F transcription factor 7	−1.12	−1.84
EGR1	Early growth response 1	−1.28	−2.09
GAS1	Growth arrest-specific 1		−2.05
GAS2L3	Growth arrest-specific 2 like 3		1.57
HIST1H2BB	Histone cluster 1, h2bb	−1.48	−2.03
HIST1H3A-J	Histone cluster 1, H3A-J	−1.34	−1.66
HIST2H2AC	Histone cluster 2, h2ac	−1.24	−1.58
HIST1H1B	Histone cluster 1, h1b	−1.18	−1.78
H1F0	H1 histone family, member 0		−1.9
HIST1H2AB, HIST1H2AE	Histone cluster 1, h2ab, histone cluster 1, h2ae		−1.79
HIST1H1C	Histone cluster 1, h1c		−1.77
HIST1H2BC, HIST1H2BI, HIST1H2BE-G	Histone cluster 1, h2bc, histone cluster 1, h2bi, histone cluster 1, h2be-g		−1.65
H1FX	H1 histone family, member X		−1.6
HIST2H4B, HIST4H4, HIST1H4A-F, HIST1H4H-L,	Histone cluster 2, h4b, histone cluster 4, H4, histone cluster 2, H4A-F histone cluster 1, H4H-L		−1.58
HIST1H2AE, HIST1H2AB	Histone cluster 1, h2ae, histone cluster 1, h2ab		−1.53
HIST1H2BN	Histone cluster 1, h2bn		2.2
H2BFXP	H2B histone family, member X, pseudogene	1.6	
INCENP	Inner centromere protein antigens 135/155 kda		1.54
JUN	Jun proto-oncogene	−1.35	−2.67
KLF10	Kruppel-like factor 10	−2.24	−2.1
KLF11	Kruppel-like factor 11	−1.35	−1.69
LOC100289187|ZNF655	Transmembrane protein 225-like, zinc finger protein 655	1.51	1.92
MYB	V-myb myeloblastosis viral oncogene homolog (avian)		−2.68
MYC	V-myc myelocytomatosis viral oncogene homolog (avian)	−1.31	−1.63
NEK2	NIMA (never in mitosis gene a)-related kinase 2		2.17
NEK6	NIMA (never in mitosis gene a)-related kinase 6	−1.24	−1.57
NOG	Noggin	−1.95	−2.35
PAK3	P21 protein (Cdc42/Rac)-activated kinase 3	1.63	1.91
PLK2	Polo-like kinase 2	−1.16	−1.64
RHOB	Ras homolog gene family, member B	−1.43	−1.81
SERPINE1	Serpin peptidase inhibitor, clade E (nexin, plasminogen activator inhibitor type 1), member 1	−1.09	−2.33
TGFB2	Transforming growth factor, beta 2	−1.2	−1.75
TGFBR1	Transforming growth factor, beta receptor 1		−1.51
TGFBR3	Transforming growth factor, beta receptor III		−1.57
THBS1	Thrombospondin 1	−1.59	−1.47
UBE2C	Ubiquitin-conjugating enzyme E2C		1.77
UBE2S	Ubiquitin-conjugating enzyme E2S		1.62
WISP2	WNT1 inducible signaling pathway protein 2	−1.26	−2.24

*Only the fold change with p<0.05 are shown.

**Table 5:** Differentially expressed genes related to replication, damage and repair of DNA.

Gene symbol	Gene name	Fold change*	
		6 h	24 h
AGTR1	Angiotensin II receptor, type 1		−1.53
BLM	Bloom syndrome, recq helicase-like		−1.66
BUB1	Budding uninhibited by benzimidazoles 1 homolog (yeast)		1.69
CDC45	Cell division cycle 45 homolog (S. Cerevisiae)	−1.26	−1.87
CDC6	Cell division cycle 6 homolog (S. Cerevisiae)		−1.69
CENPF	Centromere protein F, 350/400 kda (mitosin)		1.53
CLSPN	Claspin	−1.11	−1.84
DDB2	Damage-specific DNA binding protein 2, 48 kda	−1.22	−1.73
EXO1	Exonuclease 1		−2.25
GINS2	GINS complex subunit 2 (Psf2 homolog)	−1.16	−2.15
GINS3	GINS complex subunit 3 (Psf3 homolog)	−1.16	−1.65
HELB	Helicase (DNA) B	−1.26	−1.78
HELLS	Helicase, lymphoid-specific	−1.3	−1.97
KIF18A	Kinesin family member 18A	1.1	1.59
KIF2C	Kinesin family member 2C		−1.9
MCM10	Minichromosome maintenance complex component 10	−1.08	−1.77
MCM2	Minichromosome maintenance complex component 2	−1.15	−2.2
MCM3	Minichromosome maintenance complex component 3	−1.15	−1.53
MCM4	Minichromosome maintenance complex component 4		−1.65
MCM5	Minichromosome maintenance complex component 5	−1.14	−2.29
MCM6	Minichromosome maintenance complex component 6		−1.82
MCM7	Minichromosome maintenance complex component 7	−1.16	−1.82
NUF2	NUF2, NDC80 kinetochore complex component, homolog (S. Cerevisiae)	1.09	−1.75
ORC1|ORC1L	Origin recognition complex, subunit 1 | origin recognition complex, subunit 1-like (S. Cerevisiae)		−1.89
POLA1	Polymerase (DNA directed), alpha 1, catalytic subunit	−1.25	−1.56
POLE2	Polymerase (DNA directed), epsilon 2 (p59 subunit)	−1.15	−1.81
POLN	Polymerase (DNA directed) nu	1.85	1.56
PRIM1	Primase, DNA, polypeptide 1 (49 kda)	−1.23	−1.8
PTTG1	Pituitary tumor-transforming 1		1.77
RAD51	RAD51 homolog (S. Cerevisiae)	−1.08	−1.54
RFC2	Replication factor C (activator 1) 2, 40 kda	−1.17	−1.57
RRM2	Ribonucleotide reductase M2	−1.13	−1.58
RUVBL2	Ruvb-like 2 (E. Coli)		1.54
SPC24	SPC24, NDC80 kinetochore complex component, homolog (S. Cerevisiae)	−1.19	−1.79
STAG1	Stromal antigen 1	−1.21	−1.54
TIPIN	TIMELESS interacting protein	−1.58	−1.64
TK2	Thymidine kinase 2, mitochondrial	−1.16	−1.84
TYMS	Thymidylate synthetase	−1.18	−1.53
UNG	Uracil-DNA glycosylase	−1.1	−1.77

*Only the fold change with p<0.05 are shown.

**Table 6:** Differentially expressed genes related to apoptosis.

Gene symbol	Gene description	Fold change*	
		6 h	24 h
BAG3	BCL2-associated athanogene 3	2.12	1.91
BIRC5	Baculoviral IAP repeat-containing 5		−1.59
BLID	BH3-like motif containing, cell death inducer	−1.76	−2.2
DDIT4	DNA-damage-inducible transcript 4	2.15	2.62
DEDD2	Death effector domain containing 2	1.64	1.89
DFFA	DNA fragmentation factor, 45 kda, alpha polypeptide	1.15	1.45
DLX2	Distal-less homeobox 2	−2.63	
F3	Coagulation factor III (thromboplastin, tissue factor)	−1.56	−2.8
FOSL1	FOS-like antigen 1	−2.44	
GABARAPL1	GABA(A) receptor-associated protein like 1		−1.76
HELLS	Helicase, lymphoid-specific	−1.3	−1.97
HSPA1A, HSPA1B	Heat shock 70 kda protein 1A, heat shock 70 kda protein 1B	4.57	6.27
IER3	Immediate early response 3	−1.92	−1.28
IFI16	Interferon, gamma-inducible protein 16	−1.15	−2.01
IGFBP3	Insulin-like growth factor binding protein 3	−1.45	−2.62
IKBKE	Inhibitor of kappa light polypeptide gene enhancer in B-cells, kinase epsilon	−1.5	−1.85
JMJD6	Jumonji domain containing 6	1.36	1.73
KLF10	Kruppel-like factor 10	−2.24	−2.1
MYC	V-myc myelocytomatosis viral oncogene homolog (avian)	−1.31	−1.63
PDCD4, MIR4680	Programmed cell death 4 (neoplastic transformation inhibitor), microrna 4680		−1.51
SERPINB2, SERPINB10	Serpin peptidase inhibitor, clade B (ovalbumin), member 2 and member 10	−2.87	−5.52
SFN	Stratifin	−1.21	−2.78
SMAD6	SMAD family member 6	−2	−2.4
SMAD7	SMAD family member 7	−2.24	−2.51
SNAI2	Snail homolog 2 (Drosophila)	−1.59	−1.49
TGM2	Transglutaminase 2 (C polypeptide, protein-glutamine-gamma-glutamyltransferase)		1.99
THBS1	Thrombospondin 1	−1.59	−1.47

In addition, the analysis also revealed significantly enriched Keap1-Nrf2 pathway (*p-*value = 0.032, Table 3) which suggested that the oxidative status of cells is altered. The apoptotic pathway is another significantly altered biological process that was observed (p-value = 0.027, Table 3). The list of differentially expressed genes related to apoptosis is summarized in Table 6.

**Figure 3:**
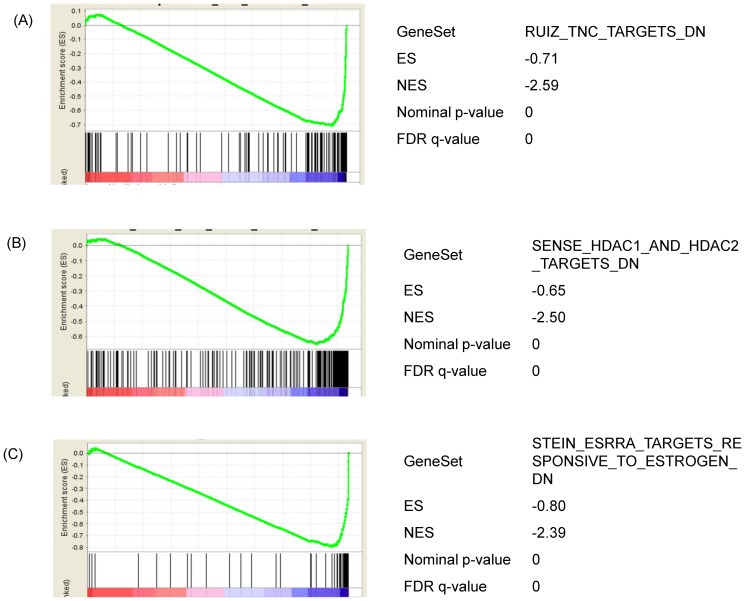
Analysis of the functional gene set enrichment after 24 h ZEA treatment by GSEA. Differential gene expression was ranked by fold change. The most up-regulated genes are shown on the left while the most down-regulated genes are shown on the right. The black vertical lines indicate where the genes in the signature get set appeared. (A) Genes that is down-regulated in the presence of extracellular matrix molecule Tenascin C. (B) Genes that are down-regulated upon knockdown of boh histone deacetylase (HDAC) 1 and 2. (C) Genes that are down-regulated by estradol and down-regulated by estrogen-related receptor alpha. Enrichment score (ES, Y axis) is a running-sum statistic showing if the prior defined set of genes are randomly distributed or found at the extremes (top or bottom) of the list. If the genes are overrepresented at the bottom of our ranked list of genes, the ES will be close to −1 and vice versa. A normalized enrichment score (NES) takes into account the number of genes in the pathway. A negative NES indicates “bottom” enrichment of the list. The interpretation of the plots referred to [61].

To further identify the dysregulated biological processes, differential regulated genes were subjected to Gene Set Enrichment Analysis (GSEA). GSEA enabled us to determine whether a priori defined set of genes is statistically significantly (with nominal p-value <0.05 and FDR<0.25) enriched after treatment with ZEA. The detailed results are shown in Table S6. Interestingly, in addition to the pathways as identified by SEA, gene sets related to the extracellular matrix molecule tenascin C [20], histone deacetylation [21] and estrogenic responses [22] were also recognized to be enriched (Figure 3).

### ZEA induced oxidative stress in BEAS-2B cells

**Figure 4:**
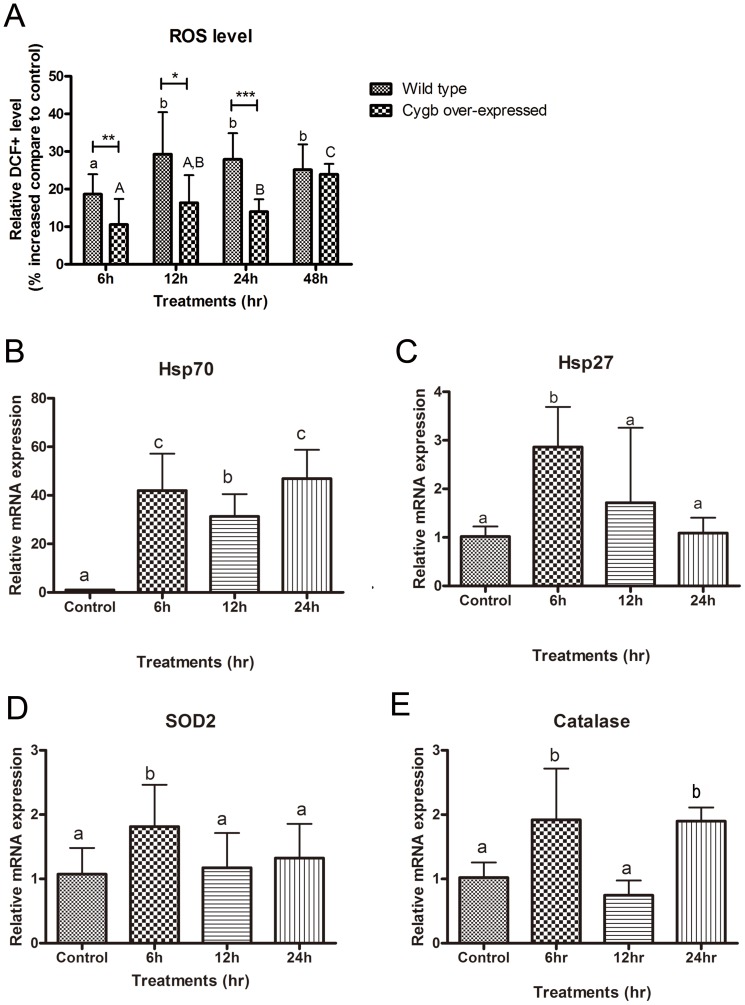
Induction of oxidative stress in BEAS-2B cells by ZEA. (A) Levels of ROS in wild type and CYGB over-expressed cells detected by DCFH-DA probe using flow cytometry. Relative DCF+ levels (equivalent to intracellular ROS levels) were expressed as percentage increased compare to DMSO control. Bars with a and b denote significant differences in wild type whereas A,B and C reflect significant differences in CYGB over-expressed cells (One-way ANOVA, *p*<0.05). *, ** and *** represent *p*<0.05, *p*<0.01 and *p*<0.001 denoting significant differences from respective wild type values. The mRNA expression of oxidative stress related genes in BEAS-2B cells were quantified by real-time PCR. The mRNA expression of β–actin was used for normalization. (B) Heat shock protein 70 (Hsp70). (C) Heat shock protein 27 (Hsp27). (D) Superoxide dismutase 2 (SOD2). (E) Catalase. Results represent the mean± SD of at least 3 independent experiments. Bars with different alphabets are significant different (One-way ANOVA, *p*<0.05).

The generation of ROS after treatment with ZEA was detected by flow cytometry. The fluorescence intensity in wild type cells that were exposed to 40 µM ZEA for 6, 12, 24 and 48 h increased in a time-dependent manner by 18.4, 29.3, 28.0 and 25.1%, respectively (Figure 4A).

Concurred with the induction of ROS levels, the expressions of selected oxidative stress-related genes were up-regulated (Figure 4B-E). The up-regulation of heat shock protein 27 (Hsp27), superoxide dismutase (SOD2) and catalase were more pronounced at 6 h and their expression were increased by 2.86, 1.81 and 1.92 folds, respectively. Moreover, the expression of heat shock protein 70 (Hsp70) was dramatically increased by 41.98 folds and the up-regulation was maintained after 12 and 24 h treatment.

### Over-expression of CYGB reduced ZEA-induced ROS generation and apoptosis in BEAS-2B cells

Over-expression of CYGB, a free radical scavenger, was used to study the role of oxidative stress upon ZEA-induced cytotoxicity. Unlike the wild type BEAS-2B cells, the detection of significantly increased level of ROS was delayed to after 24 h and 48 h ZEA exposure. In addition, the level of induction was lowered to 14.0 and 23.9%, respectively (Figure 4A). These results suggested that over-expression of CYGB can attenuate and postpone the increase of ROS levels induced by ZEA.

**Figure 5:**
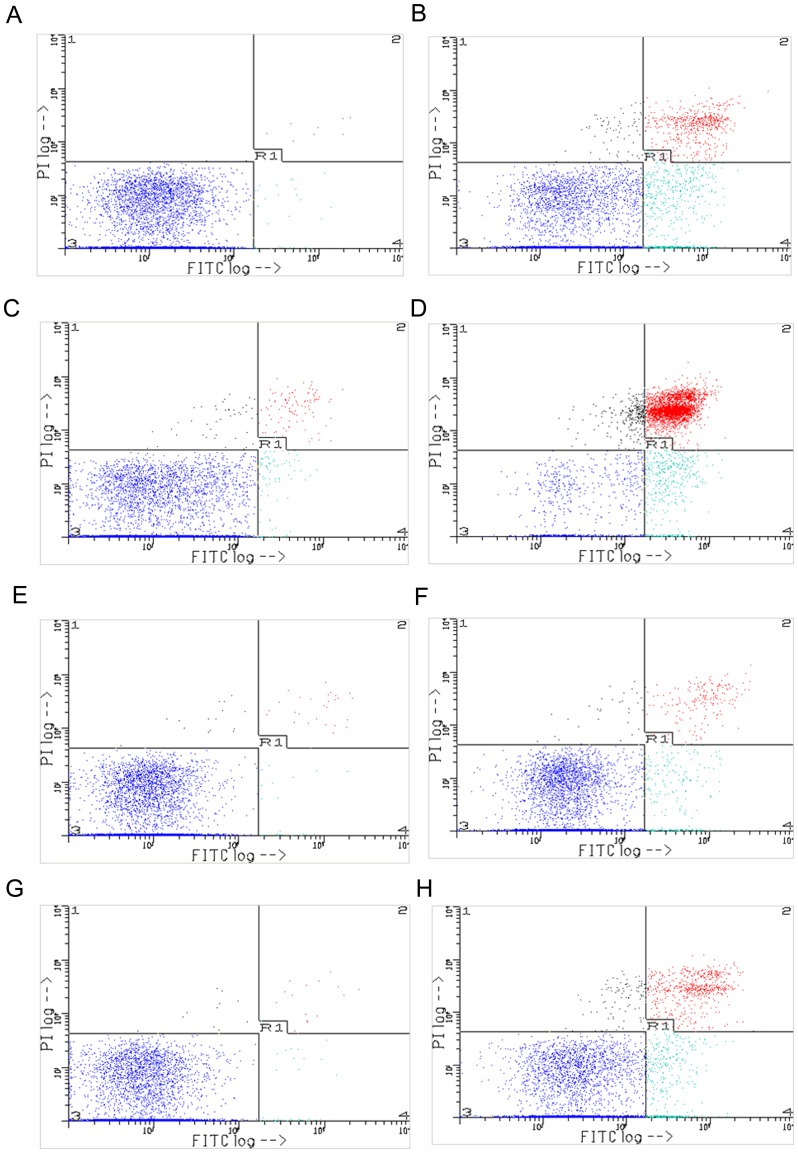
Induction of apoptosis in BEAS-2B cells by ZEA. Early apoptotic cells stained with Annexin V are shown in lower right quadrant. Late apoptotic or necrotic cells are stained with both Annexin V and PI and are shown in upper right quadrant. Living cells are not stained and are shown in lower left quadrant. (A) 24 h wild type control. (B) 24 h wild type ZEA-treated. (C) 48 h wild type control. (D) 48 h wild type ZEA-treated. (E) 24 h CYGB over-expressed control. (F) 24 h CYGB over-expressed ZEA-treated. (G) 48 h CYGB over-expressed control. (H) 48 h CYGB over-expressed ZEA-treated.

The detection of apoptotic cells was done by flow cytometry. Apoptotic cells were found in both wild type and CYGB-overexpressing cells after 24 and 48 h ZEA treatment (Figure 5). Over-expression of the endogenous free radcial scavenger, CYGB, conferred protective effects against ZEA-induced cell death. After ZEA exposure for 48 h, 13.68±2.96% of wild type whilst 74.55±0.78% of CYGB-overexpressing cells were still alive. On the other hand, 72.57±2.61% of wild type whilst 13.95±1.15% of CYGB-overexpressing cells underwent apoptosis and were at late apoptotic states.

### Validation of differentially expressed genes by qPCR

**Figure 6:**
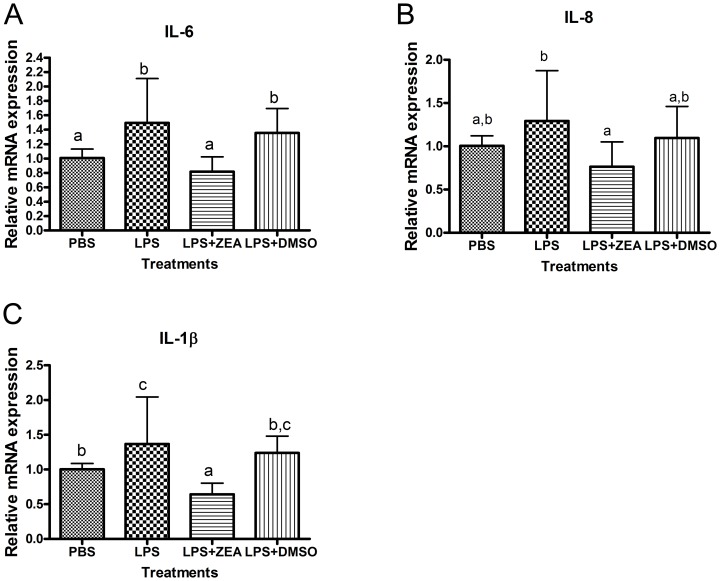
Quantitative PCR showing mRNA expression of inflammatory cytokines and chemokines in LPS stimulated BEAS-2B cells. The mRNA expression of β–actin was used for normalization. (A) Interleukin 6 (IL-6). (B) Interleukin 8 (IL-8). (C) Interleukin 1, beta (IL-1β). Results represent the mean± SD of at least 3 independent experiments and bars with different alphabets show significant differences (One-way ANOVA, *p*<0.05).

Disturbingly, many of the pro-inflammatory responsive genes were down-regulated while those anti-inflammatory genes were up-regulated (Table 7). The anti-inflammatory effects of ZEA were further revealed in its ability to reduce lipopolysaccharide (LPS)-induced release of pro-inflammatory cytokines. After 6 h treatment, LPS alone induced the expressions of IL-6, IL-8 and IL-1β by 1.49, 1.37 and 1.29 folds, respectively. However, the inductions of these cytokines were significantly suppressed by ZEA (Figure 6).

**Table 7:** Differentially expressed genes related to inflammatory responses.

Gene symbol	Gene description	Fold change*	
		6 h	24 h
ADAMTS1	ADAM metallopeptidase with thrombospondin type 1 motif, 1	−2.29	−3.32
BCL3	B-cell CLL/lymphoma 3	−1.32	−1.79
BMPER	BMP binding endothelial regulator	−1.49	−2.21
CBLB	Cas-Br-M (murine) ecotropic retroviral transforming sequence b		−1.56
CCL2	Chemokine (C-C motif) ligand 2		−1.57
CD24	CD24 molecule		−2.18
CT45A5, CT45A2, CT45A3, CT45A6, CT45A4, CT45A1	Cancer/testis antigen family 45, member A5, cancer/testis antigen family 45, member A2, cancer/testis antigen family 45, member A3, cancer/testis antigen family 45, member A6, cancer/testis antigen family 45, member A4, cancer/testis antigen family 45, member A1	1.54	
CXCR7	Chemokine (C-X-C motif) receptor 7	−1.28	−1.68
DKK1	Dickkopf WNT signaling pathway inhibitor 1		−2.9
DUSP22	Dual specificity phosphatase 22	−1.19	−1.56
EDN1	Endothelin 1	−2.57	−1.53
ELF4	E74-like factor 4 (ets domain transcription factor)	−1.54	−1.54
F2RL2	Coagulation factor II (thrombin) receptor-like 2	−1.73	−3.92
F3	Coagulation factor III (thromboplastin, tissue factor)	−1.56	−2.8
FCER1G	Fc fragment of ige, high affinity I, receptor for; gamma polypeptide		1.77
FSTL3	Follistatin-like 3 (secreted glycoprotein)	−1.46	−1.87
IFI16	Interferon, gamma-inducible protein 16	−1.15	−2.01
IFIT1	Interferon-induced protein with tetratricopeptide repeats 1		2.29
IFITM1	Interferon induced transmembrane protein 1 (9–27)		−1.94
IGHV4-31, IGHG1, IGHA1, IGH@, IGHJ2	Immunoglobulin heavy variable 4–31, immunoglobulin heavy constant gamma 1 (G1m marker), immunoglobulin heavy constant alpha 1, immunoglobulin heavy locus, immunoglobulin heavy joining 2	1.64	1.17
IGSF23	Immunoglobulin superfamily, member 23	−1.12	−1.66
IGSF3	Immunoglobulin superfamily, member 3	−1.26	−1.62
IKBKE	Inhibitor of kappa light polypeptide gene enhancer in B-cells, kinase epsilon	−1.5	−1.85
IL11	Interleukin 11		2.1
IL1R1	Interleukin 1 receptor, type I		−1.53
IL27RA	Interleukin 27 receptor, alpha	−1.45	−1.68
IL31RA	Interleukin 31 receptor A	−1.18	−1.52
IL37	Interleukin 37	1.53	1.77
IL6R	Interleukin 6 receptor	−1.23	−1.55
IL7R	Interleukin 7 receptor	−1.5	−2.52
IL8	Interleukin 8	−2.22	−1.7
IRAK4	Interleukin-1 receptor-associated kinase 4	−1.13	−1.69
ITGB3	Integrin, beta 3 (platelet glycoprotein iiia, antigen CD61)		1.95
JUN	Jun proto-oncogene	−1.35	−2.67
KLF10	Kruppel-like factor 10	−2.24	−2.1
LAMC2	Laminin, gamma 2	−1.34	−1.7
LRIG3	Leucine-rich repeats and immunoglobulin-like domains 3	1.19	−1.75
LY6K	Lymphocyte antigen 6 complex, locus K	−1.25	−2.02
NCR3LG1	Natural killer cell cytotoxicity receptor 3 ligand 1	−1.23	−1.94
NOG	Noggin	−1.95	−2.35
OAS3	2'-5'-oligoadenylate synthetase 3, 100 kda	−1.2	−1.65
PLAU	Plasminogen activator, urokinase	−2.79	−1.6
SEMA3C	Sema domain, immunoglobulin domain (Ig), short basic domain, secreted, (semaphorin) 3C		−1.59
SEMA3D	Sema domain, immunoglobulin domain (Ig), short basic domain, secreted, (semaphorin) 3D	−1.18	−1.65
SMAD6	SMAD family member 6	−2	−2.4
SMAD7	SMAD family member 7	−2.24	−2.51
SMAD9	SMAD family member 9	−1.5	−1.58
TGFB2	Transforming growth factor, beta 2	−1.2	−1.75
TGFBR1	Transforming growth factor, beta receptor 1		−1.51
TNFAIP8L1	Tumor necrosis factor, alpha-induced protein 8-like 1		1.88

*Only the fold change with p<0.05 are shown.

**Figure 7:**
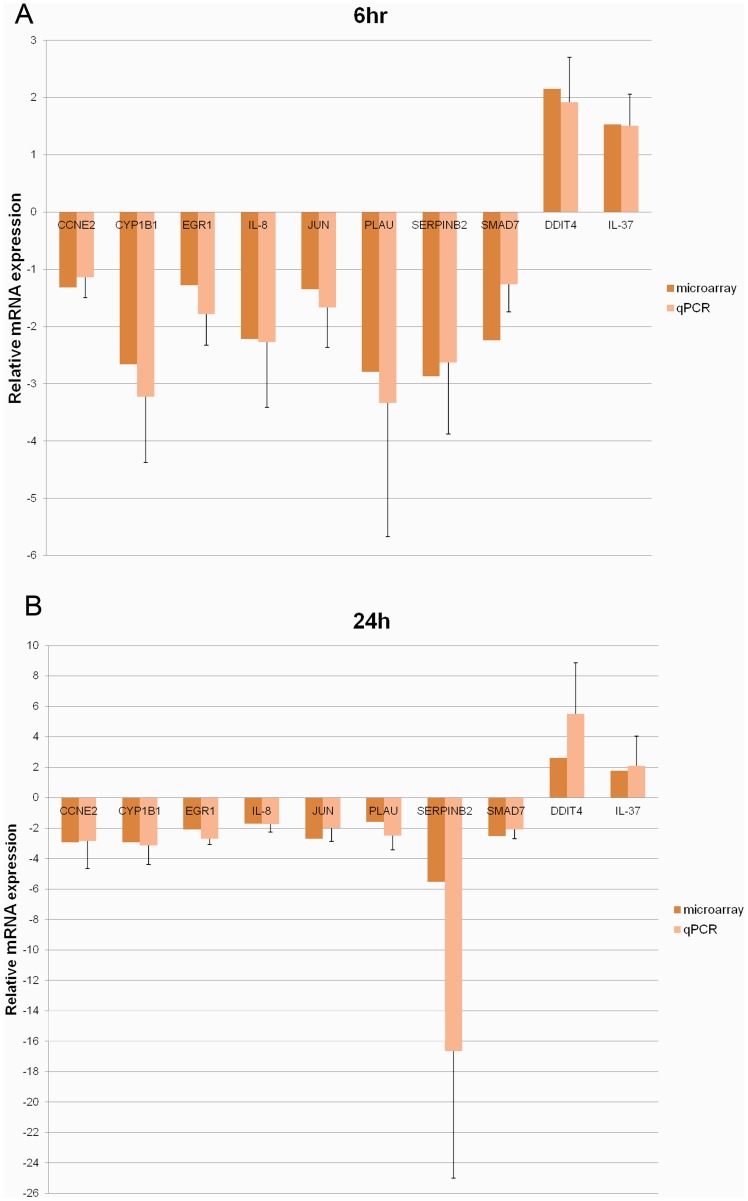
Validation of the expressions of selected genes by real-time PCR. (A) 6 hr. (B) 24 hr. Real-time PCR results are represented as mean± SD of at least 3 independent experiments

After analyzing the microarray data, the expression changes of 10 selected genes including SERPINB2 and PLAU (apoptosis), CYP1B1 (aryl hydrocarbon receptor signaling), SMAD7 (TGF-β signaling), IL-8 and IL-37 (inflammatory response), JUN and EGR1 (regulation of transcription), CCNE2 (progression of cell cycle) and DDIT4 (response to DNA damage) were verified by quantitative real-time PCR (qPCR). The expression pattern (direction of regulation) showed a good agreement between the data of microarray and qPCR, although the fold-changes detected by qPCR appeared to be more pronounced (Figure 7).

### ZEA caused global DNA hypomethylation in BEAS-2B cells

**Figure 8:**
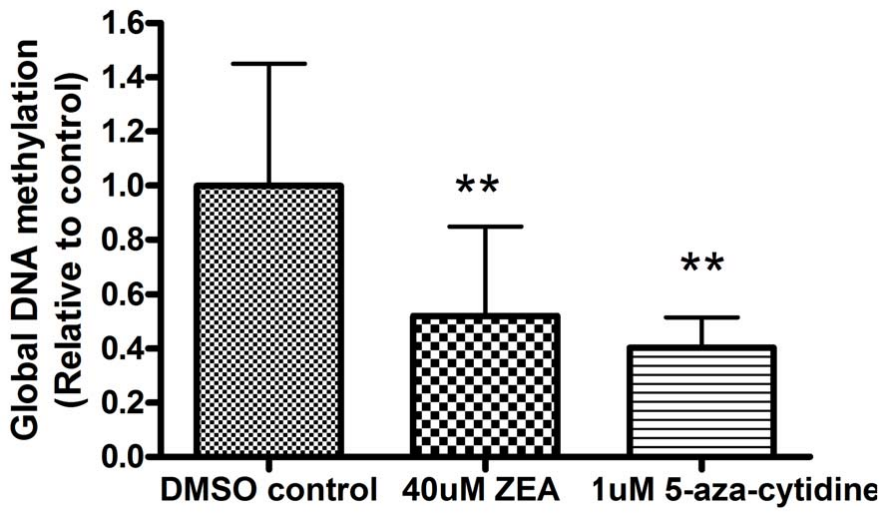
Induction of global DNA demethylation in BEAS-2B cells by exposure to ZEA for 24 h. Results represent the mean± SD of at least 3 independent experiments and ** represents p<0.01 significantly different from DMSO control values as assessed by t-test.

As shown in Figure 8, the level of global DNA methylation was significantly lowered to 40.3±11.1% (*p*<0.01) and 53.09±33.75% (*p*<0.01) relative to control in cells treated with 1 µM 5-aza-cytidine or 40 µM ZEA respectively. These results suggest that global DNA demethylation occurs when BEAS-2B cells are exposed to ZEA.

## Discussion

The aim of this study is to decipher the toxic effects and molecular mechanisms induced by ZEA in human bronchial epithelial cells (BEAS-2B). Our experiments and biological interpretation of the genome-wide transcriptome analysis indicated that induction of oxidative stress, arrest of cell cycle progression, initiation of apoptosis, suppression of inflammatory responses and changes of epigenetic marks are the consequences of exposure to ZEA.

### Induction of oxidative stress by ZEA

The broad range of toxic events provoked by ZEA is believed not solely due to the interaction with estrogen receptors but also through the induction of oxidative stress. Our results indicated that the Keap1-Nrf2 pathway was suppressed (Table 3), as the levels of intracellular ROS was increased (Figure 4A) and the expression of free radical scavengers, SOD2 and catalase, were up-regulated (Figure 4D and E) after ZEA treatment. Nrf2 is a transcription factor that binds to the antioxidant responsive element at the regulatory region of target genes that confer protection against oxidative stress [23]. Nrf2 is normally sequestered and inhibited at the cytoplasm by Keap1. However, under stress conditions, Nrf2 dissociates from Keap1 and translocates to the nucleus to control gene expression [23]. The expression of SOD2 and catalase are up-regulated under oxidative stress by the binding of Nrf2 to their promoters [24]. In addition, our results show dramatic up-regulation of heat shock proteins, which are early markers of oxidative stress [25], [26].

On the other hand, the generation of ROS (Figure 4A) and the induction of apoptosis (Figure 5) were significantly attenuated by the over expression of CYGB, a potent free radical scavenger. Collectively, our results suggest that ZEA is a strong inducer of ROS and oxidative stress is the underlying mediator of ZEA-induced cytotoxicity. These observations substantiate early reports showing that exogenous antioxidants including vitamin E and Tunisian radish protect against ZEA-induced oxidative damage and subsequent apoptosis [27], [28].

The mechanism by which ZEA induces production of ROS is ambiguous. It is known that upon metabolism of ZEA, 3α-/3β-hydroxysteroid dehydrogenases catalyze the formation of two major reductive metabolites, α– and β-Zearalenol [29]. Pfeiffer et al [30] identified two highly unstable oxidative metabolites of ZEA, 13-hydroxy-ZEA and 15-hydroxy ZEA, which are demonstrated to possess the same potency for causing oxidative DNA damage (as measured by the level of 8-oxo-2′-deoxyguanosine of DNA) as catechols of estradiols [31]. Therefore, we believe that ROS could be generated during the formation of these metabolites. In addition, ROS may be generated due to the alteration of mitochondrial NADPH-oxidase which functions to generate superoxide anions (O_2_^−^) from normal oxygen [32]. Our array results support this supposition as the expression of NADPH-oxidase (NOX5) is up-regulated by 1.32 and 1.6 fold upon 6 and 24 h exposure to ZEA (Table S2 and S3).

### DNA damages and inhibition of DNA repair by ZEA

Excessive generation of ROS can oxidize cellular macromolecules including DNA, protein and lipids. The ability of ZEA to cause oxidative DNA damage including DNA fragmentation, single- and double stranded breakage as well as formation of 8-oxoguanine were reported [9], [27], [33]. Our microarray results also signified the suppression of DNA repair and induction of DNA damage (Table 3 and 5). The expression of breast cancer 1, early onset (BRCA1), RAD51, bloom syndrome, RecQ helicase-like (BLM), flap-endonuclease 1 (FEN1), uracil-DNA glycosylase (UNG), damage-specific DNA binding protein 2 (DDB2) and exonuclease 1 (EXO1) were significantly suppressed after 24 h ZEA treatment (Table 5).

BRCA1 functions in response to the signal of DNA damage and transcriptionally control downstream effectors [34], [35]. RAD51 and BLM are involved in the repair of DNA double-strand break through homologous recombination [36], [37]. BRCA1 and RAD51 also interact to control recombination and maintain the integrity of the genome [38]. FEN1 [39], UNG [40], DDB2 [41] and EXO1 [42] are involved in DNA base-excision repair, DNA mismatch repair and homologous recombination. On the other hand, DNA-damage-inducible transcript 4 (DDIT4) and never in mitosis gene a-related kinase 2 (NEK2) which are involved in controlling DNA damage checkpoint and proper DNA repair, are significantly induced by 2.62 and 2.17 folds respectively after 24 h ZEA treatment (Table 5). Our transcriptome analysis suggested that ZEA inhibited DNA repair and induced DNA damages.

Our microarray data also revealed that the progression of cell cycle and replication of DNA in BEAS-2B cells was suppressed by ZEA (Table 4 and 5). The expressions of cyclin E (CCNE1, CCNE2), which are essential for S phase progression, are significantly down-regulated by 2.11 and 2.93 folds respectively while p21 activated kinase 3 (PAK3), a known inhibitor of Cdk4, is simultaneously induced by 1.91 folds after 24 h of ZEA treatment. These observations suggest that cyclinD/cdk4 and cyclinE/cdk2 complexes which are essential for G1/S cell cycle progression have been suppressed.

### Arrest of cell cycle by ZEA

In addition, many genes related to DNA replication, which are also essential for S phase progression, are down-regulated after 24 h of ZEA treatment (Table 5). The essential components of the pre-replicative complex (pre-RC) including origin recognition complex, subunit 1 (ORC1), cell division cycle 6 (CDC6) and most member of minichromosome maintenance protein (MCM 2–7) show decreased levels of expression (Table 5). In addition, the assembly and activation complex comprising MCM 10, CDC45, replication factor C (activator 1) 2 (RFC2) and interaction with subunits of replicative polymerase, including polymerase (DNA directed), alpha 1 (POLA1), polymerase (DNA directed), epsilon 2 (POLE2) and primase, DNA, polypeptide 1 (PRIM1), are also down-regulated (Table 5). DNA helicase B (HELB), which is involved in DNA synthesis, is down-regulated by 1.78 folds (Table 5). From the above results, it can be concluded that ZEA directly affects the machinery for DNA replication and synthesis in BEAS-2B cells.

**Figure 9:**
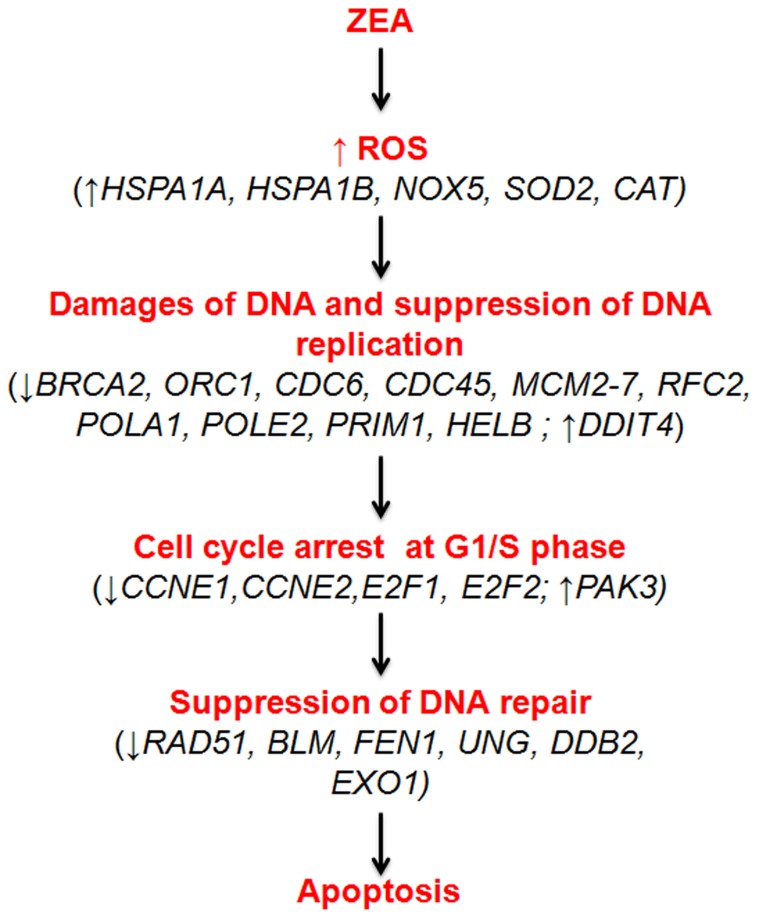
Proposed mechanisms of apoptosis induced by ZEA in BEAS-2B cells. The expression changes of genes associated with the biological processes are indicated.

Taken together, it is tempting to speculate that ZEA induces DNA damage and halts cell cycle at G1/S phase as attempts to repair the damage are unsuccessful. These findings are in agreement with earlier studies which reported that ZEA induced DNA fragmentation and cell cycle arrest [27], [33]. The presence of irreparable DNA lesions may lead to the occurrence of subsequent apoptosis in the affected cells (Figure 9).

### Down-regulation of inflammatory responses by ZEA

Due to the large surface structure of the lung, its innate immune response is essential as the first line of defense to act against environmental particles, allergens and invasion of pathogens. The responses often include production of cytokines and chemokines to recruit inflammatory effector cells such as macrophages, neutrophils, eosinophils and lymphocytes [43]. Our results indicated that ZEA weakened the innate immune response to bacterial pathogens (LPS) in bronchial epithelial cells through down-regulating the expression of various inflammatory genes (Figure 6 and Table 7). The mechanism of this down-regulation is possibly through the inhibition of the toll-like receptor (TLR) signaling pathway (Table 3). TLR is a cell surface member of the pattern recognition receptors (PRRs) which are essential as first line defense of the host's responses to allergens and pathogens [44], [45]. IL-1 receptor-associated kinase 4 (IRAK4) is an intrinsic kinase which mediates inhibition of IL-1/TLR induced NF-κB activation [44]. Upon activation and stimulation of TLRs, association of MyD88 recruits IRAK4 which in turn induces the phosphorylation of IRAK1 and triggers downstream activation of NF-κB [44]. In our microarray results, the expression of both MyD88 and IRAK4 are respectively down-regulated by 1.45 and 1.69 folds, pointing to the upstream suppression of TLR signaling and subsequent activation of NF-κB.

Both suppressive and inductive effects of ZEA on inflammatory responses have been reported [46], [47], [48] albeit with different models, dosage and duration of incubation with ZEA. Oxidative stress could trigger inflammation signals through activation of transcription regulators, NF-κB and AP-1 [49]. In our results, however, ZEA induced ROS generations but suppressed inflammatory responses. These observations could be explained by the suppression of NF-κB activation through inhibition of the TLR signaling Myd88-dependent pathway as aforementioned. The finding is consistent with earlier proteomic study in H295R cells showing possible suppression of NF-κB pathway after ZEA exposure [50]. Importantly, our results also indicate that the expressions of JUN, FOSL1 and ATF3 which form the AP-1 transcription complex are significantly decreased (Table 7) suggesting that the AP-1 activation is possibly inhibited by ZEA.

These observations suggested that exposure to air-borne ZEA may increase susceptibility of bronchial epithelial cells to infections due to down-regulation of the expression of inflammatory cytokines and chemokines.

### Potential epigenetic changes by ZEA

On the other hand, GSEA of the array results indicated that the histone deacetylation pathway is altered (Figure 3). Histone deacetlyation is associated with pathogenesis of lung diseases. For example, in COPD, the progressive reduction of HDAC activity is linked to the severity of the disease [51], [52], [53]. Methylated promoters are often coupled with regional histone deacetylation and contribute to transcriptional inactivation. Global hypomethylation was also observed in BEAS-2B cells incubated with ZEA (Figure 8). Possibly, the presence of 8-OHdG [54], [55] and O^6^-methylguanine formed during ROS-induced DNA damage prevented the methylation of adjacent cytosine residues [56], [57], [58]. Disturbingly, global DNA hypomethylation is a feature of tumorigenesis [59], [60]. The role of DNA hypomethylation in the development of cancer is still a paradox. Demethylation of DNA usually occurs at intragenic regions and at repetitive DNA sequences. Three mechanisms including causing instability of chromosome, reactivation of transposable elements and loss of imprinting are proposed [60]. More importantly, altered DNA methylation levels can be stably inherited during DNA replication and disturb subsequent generations. Our results raised the concern on long-lasting effect of ZEA to lung cells which required further investigation.

## Conclusions

Our results clearly pointed out the diverse biological responses that ensued when BEAS-2B lung epithelial cells are exposure to ZEA. It also gives us an insight into the molecular mechanisms underlying the adverse consequences of air-borne ZEA. Disturbingly, our results suggested that exposure to ZEA may increase susceptibility of bronchial epithelial cells to diseases through i) the down-regulation of inflammatory cytokines and ii) demethylation of DNA which is a feature of lung carcinogenesis.

## Supporting Information

Table S1 **Primers used for real-time quantitative PCR.**
(DOCX)

Table S2 **The complete list of differentially expressed genes (fold change >1.5, ANOVA p-value <0.05) in BEAS-2B cells after 6 h treatment.**
(DOCX)

Table S3 **The complete list of differentially expressed genes (fold change >1.5, ANOVA p-value <0.05) in BEAS-2B cells after 24 h treatment.**
(DOCX)

Table S4 **Summary of the enriched gene ontology (GO) terms in BEAS-2B cells after 6 h treatment with ZEA.**
(DOCX)

Table S5 **Summary of the enriched gene ontology (GO) terms in BEAS-2B cells after 24 h treatment with ZEA.**
(DOCX)

Table S6 **Pathways enriched in BEAS-2B cells after 24 h treatment with ZEA.**
(DOC)
