# Supplementary material for: Gene Expression Profile and Toxic Effects in Human Bronchial Epithelial Cells Exposed to Zearalenone
Source: PLoS One. 2014 May 2;9(5):e96404. doi: 10.1371/journal.pone.0096404 (PMC4008614; doi:10.1371/journal.pone.0096404)
Supplement: Table S1 — Primers used for real-time quantitative PCR. (DOCX) [file pone.0096404.s001.docx]

Table S1. Primers used for real-time quantitative PCR

| **Gene*** | **Sequence (5’→3’)** | | **Amplicon (bp)** | **Accession no.** |
| --- | --- | --- | --- | --- |
|  |  |  |  |  |
| ACTB | F | CATGTACGTTGCTATCCAGGC | 250 | NM_001101.3 |
|  | R | CTCCTTAATGTCACGCACGAT |  |  |
| CAT | F | GCCAACTACCAGCGTGAC | 187 | NM_001753.3 |
|  | R | ATGCCCGCACCTGAGTAA |  |  |
| CCNE2 | F | ATTGCTTCCAAACTTGAG | 158 | NM_057749.2 |
|  | R | AGCCAGGAGATGATTGTT |  |  |
| CYGB | F | TGGCCATCCTGGTGAGGTT | 121 | NM_134268.3 |
|  | R | CAGGCGTGCTTCCGCAGCT |  |  |
| CYP1B1 | F | TGGCTGCTCCTCCTCTTC | 354 | NM_000104.3 |
|  | R | GCTGGTCAGGTCCTTGTT |  |  |
| DDIT4 | F | GTCGTCCACCTCCTCTTC | 243 | NM_019058.2 |
|  | R | TGTTCATCCTCAGGGTCAC |  |  |
| EGR1 | F | CAGCAGTCCCATTTACTCAG | 345 | NM_001964.2 |
|  | R | GACTGGTAGCTGGTATTG |  |  |
| HSP27 | F | TGCTTCACGCGGAAATACA | 148 | NM_001540.3 |
|  | R | CGAAGGTGACTGGGATGGT |  |  |
| HSP70 | F | GAACAAGCGAGCCGTGAG | 177 | NM_005345.5 |
|  | R | AGGGTGCTTCGGAACAGG |  |  |
| IL-1β | F | TACAGCAAGGGCTTCAGG | 192 | NM_000576.2 |
|  | R | TCGTACAGGTGCATCGTG |  |  |
| IL-37 | F | GAAGAACTTAAACCCGAAGA | 248 | NM_014439.3 |
|  | R | GCTGAAGGGATGGATGAC |  |  |
| IL-6 | F | ATGGATGCTTCCAATCTG | 127 | NM_000600.3 |
|  | R | CTGGCTTGTTCCTCACTAC |  |  |
| IL-8 | F | AGACATACTCCAAACCTT | 111 | NM_000584 |
|  | R | CTCTTCCATCAGAAAGCT |  |  |
| JUN | F | CAGGTGGCACAGCTTAAACA | 80 | NM_002228 |
|  | R | GTT TGCAACTGCTGCGTT AG |  |  |
| PLAU | F | GTGAGATCACTGGCTTTGG | 218 | NM_001145031 |
|  | R | TTGGAGGGAACAGACGAG |  |  |
| SERPINB2 | F | TGAGAAGTCTGCGAGCTT | 147 | NM_001143818.1 |
|  | R | TTGCCTTTGGTTTGAGTC |  |  |
| SOD2 | F | CTTTGGCTACTTTGAGGT | 246 | NM_000636.2 |
|  | R | GATGGAAACAATATGGGAT |  |  |
| SMAD6 | F | GAAACGGAGGCTACCAAC | 148 | NM_001142861 |
|  | R | CGTACACCGCATAGAGGC |  |  |

*The abbreviations of genes are: ACTB, β-actin; CAT, catalase; CCNE2, cyclin E2; CYGB, cytoglobin; CYP1B1, cytochrome P450, family 1, subfamily B, polypeptide 1; DDIT4,DNA damage inducible transcript 4; EGR1, early growth response 1; HSP, heat shock protein; IL, interleukin; PLAU, plasminogen activator, urokinase; SERPINB2, serpin peptidase inhibitor, clade B (ovalbumin), member 2; SOD2, superoxide dismutase 2.
