# Supplementary material for: Gene Expression Profile and Toxic Effects in Human Bronchial Epithelial Cells Exposed to Zearalenone
Source: PLoS One. 2014 May 2;9(5):e96404. doi: 10.1371/journal.pone.0096404 (PMC4008614; doi:10.1371/journal.pone.0096404)
Supplement: Table S2 — The complete list of differentially expressed genes (fold change >1.5, ANOVA p-value <0.05) in BEAS-2B cells after 6 h treatment. (DOCX) [file pone.0096404.s002.docx]

Table S2. The complete list of differentially expressed genes (fold change >1.5, ANOVA p-value <0.05) in BEAS-2B cells after 6h treatment with ZEA

| **Gene symbol** | **Gene description** | **Fold change** | **ANOVA p-value** | **FDR p-value** |
| --- | --- | --- | --- | --- |
| ***Down-regulated genes*** | | | | |
| ADAM1 | ADAM metallopeptidase domain 1, pseudogene | -1.64 | 0.02442 | 0.283708 |
| ADAMTS1 | ADAM metallopeptidase with thrombospondin type 1 motif, 1 | -2.29 | 0.000624 | 0.075125 |
| ADAMTS15 | ADAM metallopeptidase with thrombospondin type 1 motif, 15 | -1.6 | 0.001198 | 0.091615 |
| ADRB2 | Adrenoceptor beta 2, surface | -1.96 | 0.000763 | 0.081379 |
| ANKRD1 | Ankyrin repeat domain 1 (cardiac muscle) | -1.87 | 0.001326 | 0.093842 |
| ATAD3A | Atpase family, AAA domain containing 3A | -1.64 | 0.002156 | 0.113318 |
| ATOH8 | Atonal homolog 8 (Drosophila) | -1.61 | 0.001034 | 0.08889 |
| BIRC3 | Baculoviral IAP repeat containing 3 | -1.58 | 0.025666 | 0.289747 |
| BLID | BH3-like motif containing, cell death inducer | -1.76 | 0.009505 | 0.200248 |
| C1QTNF2 | C1q and tumor necrosis factor related protein 2 | -1.57 | 0.002331 | 0.115679 |
| C6orf48, SNORD52 | Chromosome 6 open reading frame 48, small nucleolar RNA, C/D box 52 | -1.52 | 0.00545 | 0.161932 |
| CD44 | CD44 molecule (Indian blood group) | -1.59 | 0.000455 | 0.067232 |
| CDK15 | Cyclin-dependent kinase 15 | -1.63 | 0.000217 | 0.053751 |
| CEACAMP6, CEACAMP7, CEACAMP9, LOC284344, CEACAMP11 | Carcinoembryonic antigen-related cell adhesion molecule pseudogene 6, carcinoembryonic antigen-related cell adhesion molecule pseudogene 7, carcinoembryonic antigen-related cell adhesion molecule pseudogene 9, uncharacterized LOC284344, carcinoembryonic antigen-related cell adhesion molecule pseudogene 11 | -1.71 | 0.008466 | 0.191216 |
| CLDN12, CDK14 | Claudin 12, cyclin-dependent kinase 14 | -1.66 | 0.03608 | 0.32775 |
| CLTC-IT1 | CLTC intronic transcript 1 (non-protein coding) | -1.78 | 0.037522 | 0.331548 |
| CYP1B1 | Cytochrome P450, family 1, subfamily B, polypeptide 1 | -2.66 | 0.000007 | 0.018228 |
| DIO2 | Deiodinase, iodothyronine, type II | -1.67 | 0.000055 | 0.039062 |
| DIP2A-IT1 | DIP2A intronic transcript 1 (non-protein coding) | -1.53 | 0.001022 | 0.08889 |
| DLX1 | Distal-less homeobox 1 | -1.71 | 0.001723 | 0.103251 |
| DLX2 | Distal-less homeobox 2 | -2.63 | 0.00007 | 0.039062 |
| DOCK11 | Dedicator of cytokinesis 11 | -1.62 | 0.002198 | 0.114068 |
| DOCK2 | Dedicator of cytokinesis 2 | -1.51 | 0.000004 | 0.015992 |
| EDN1 | Endothelin 1 | -2.57 | 0.000069 | 0.039062 |
| ELF4 | E74-like factor 4 (ets domain transcription factor) | -1.54 | 0.000146 | 0.047505 |
| EPHA2 | EPH receptor A2 | -1.52 | 0.009098 | 0.196162 |
| EREG | Epiregulin | -1.98 | 0.001303 | 0.093821 |
| F2RL2 | Coagulation factor II (thrombin) receptor-like 2 | -1.73 | 0.000141 | 0.047283 |
| F3 | Coagulation factor III (thromboplastin, tissue factor) | -1.56 | 0.000615 | 0.074997 |
| FAM83G | Family with sequence similarity 83, member G | -1.97 | 0.000147 | 0.047505 |
| FBXO32 | F-box protein 32 | -1.75 | 0.012958 | 0.227974 |
| FLJ22447 | Uncharacterized LOC400221 | -1.62 | 0.004095 | 0.144489 |
| FLJ35946 | Uncharacterized protein FLJ35946 | -1.57 | 0.000538 | 0.071867 |
| FLJ38717 | FLJ38717 protein | -1.8 | 0.029212 | 0.303512 |
| FOSL1 | FOS-like antigen 1 | -2.44 | 0.000011 | 0.022705 |
| FOXC2 | Forkhead box C2 (MFH-1, mesenchyme forkhead 1) | -1.67 | 0.002207 | 0.1142 |
| HES1 | Hairy and enhancer of split 1, (Drosophila) | -1.64 | 0.002115 | 0.112395 |
| HIVEP1 | Human immunodeficiency virus type I enhancer binding protein 1 | -1.74 | 0.004481 | 0.149658 |
| HLF | Hepatic leukemia factor | -1.65 | 0.000656 | 0.076367 |
| HMGA2 | High mobility group AT-hook 2 | -1.62 | 0.004537 | 0.150536 |
| HTR7 | 5-hydroxytryptamine (serotonin) receptor 7, adenylate cyclase-coupled | -1.53 | 0.029855 | 0.306675 |
| IER3 | Immediate early response 3 | -1.83 | 0.000047 | 0.03752 |
| IL8 | Interleukin 8 | -2.22 | 0.0107 | 0.209812 |
| KIAA1644 | Kiaa1644 | -1.62 | 0.000081 | 0.039331 |
| KLF10 | Kruppel-like factor 10 | -2.24 | 0.00018 | 0.050844 |
| KLF2 | Kruppel-like factor 2 (lung) | -1.58 | 0.000711 | 0.077977 |
| KLF9 | Kruppel-like factor 9 | -1.95 | 0.000025 | 0.031824 |
| KRT80 | Keratin 80 | -2.11 | 0.000156 | 0.048316 |
| KRTAP4-8 | Keratin associated protein 4-8 | -1.54 | 0.046267 | 0.359836 |
| L3MBTL2 | L(3)mbt-like 2 (Drosophila) | -1.51 | 0.005741 | 0.16477 |
| LIF | Leukemia inhibitory factor | -1.69 | 0.005446 | 0.161932 |
| LOC100128002 | Uncharacterized LOC100128002 | -1.81 | 0.025527 | 0.288998 |
| LOC100128979 | Uncharacterized LOC100128979 | -1.52 | 0.016696 | 0.246818 |
| LOC100129940 | Uncharacterized LOC100129940 | -1.79 | 0.000676 | 0.076367 |
| LOC100131234 | Familial acute myelogenous leukemia related factor | -2.04 | 0.000084 | 0.03948 |
| LOC100133106 | Vcew9374 | -1.88 | 0.000015 | 0.026264 |
| LOC100289650 | Uncharacterized LOC100289650 | -1.96 | 0.001758 | 0.103801 |
| LOC100505701 | Uncharacterized LOC100505701 | -1.87 | 0.00087 | 0.085407 |
| LOC100506303, LOC400879 | Uncharacterized LOC100506303, uncharacterized LOC400879 | -1.68 | 0.00115 | 0.089851 |
| LOC100506303, LOC400879, LOC100653149 | Uncharacterized LOC100506303, uncharacterized LOC400879, uncharacterized LOC100653149 | -1.75 | 0.001064 | 0.08889 |
| LOC100506870 | Uncharacterized LOC100506870 | -1.52 | 0.000371 | 0.065767 |
| LOC100507460 | Uncharacterized LOC100507460 | -1.56 | 0.006875 | 0.177982 |
| LOC100507507 | Uncharacterized LOC100507507 | -1.55 | 0.004426 | 0.148747 |
| LOC100507516 | Uncharacterized LOC100507516 | -1.67 | 0.002591 | 0.120363 |
| LOC100509976 | Uncharacterized LOC100509976 | -1.7 | 0.008672 | 0.192943 |
| LOC100653008, LOC100652914 | Uncharacterized LOC100653008, uncharacterized LOC100652914 | -1.66 | 0.010689 | 0.209802 |
| LOC145694 | Uncharacterized LOC145694 | -1.94 | 0.001542 | 0.099275 |
| LOC283140 | Uncharacterized LOC283140 | -1.74 | 0.002185 | 0.113854 |
| LOC284926 | Uncharacterized LOC284926 | -1.52 | 0.008015 | 0.187579 |
| LOC400684 | Uncharacterized LOC400684 | -1.53 | 0.02363 | 0.280556 |
| LOC441956 | Uncharacterized LOC441956 | -1.56 | 0.005184 | 0.159766 |
| LOC644649 | Apolipoprotein O pseudogene | -1.55 | 0.012717 | 0.22545 |
| LOC730755, KRTAP2-4 | Keratin associated protein 2-4-like, keratin associated protein 2-4 | -3.28 | 0.000077 | 0.039331 |
| MARCH4 | Membrane-associated ring finger (C3HC4) 4, E3 ubiquitin protein ligase | -1.58 | 0.002814 | 0.125451 |
| MFAP3 | Microfibrillar-associated protein 3 | -1.52 | 0.000645 | 0.07598 |
| MIR1305 | Microrna 1305 | -1.52 | 0.034421 | 0.323158 |
| MIR181B1 | Microrna 181b-1 | -1.89 | 0.000021 | 0.030243 |
| MIR221 | Microrna 221 | -1.68 | 0.000356 | 0.064833 |
| MIR24-2, LOC284454 | Microrna 24-2, uncharacterized LOC284454 | -1.78 | 0.00086 | 0.085407 |
| MIR27B, C9orf3 | Microrna 27b, chromosome 9 open reading frame 3 | -1.74 | 0.000059 | 0.039062 |
| MIR3167 | Microrna 3167 | -1.65 | 0.038161 | 0.334265 |
| MIR378D2 | Microrna 378d-2 | -1.58 | 0.044391 | 0.355317 |
| MIR4653 | Microrna 4653 | -1.55 | 0.003185 | 0.130933 |
| MIRLET7F2 | Microrna let-7f-2 | -1.62 | 0.010268 | 0.206235 |
| MN1 | Meningioma (disrupted in balanced translocation) 1 | -1.93 | 0.000506 | 0.069867 |
| MPP4 | Membrane protein, palmitoylated 4 (MAGUK p55 subfamily member 4) | -1.76 | 0.040044 | 0.340375 |
| NAG20 | Nag20 | -1.67 | 0.027393 | 0.297038 |
| NAV3 | Neuron navigator 3 | -1.52 | 0.001464 | 0.097409 |
| NCKAP5-IT1 | NCKAP5 intronic transcript 1 (non-protein coding) | -1.7 | 0.002033 | 0.110773 |
| NOG | Noggin | -1.95 | 0.00047 | 0.06844 |
| NOL8 | Nucleolar protein 8 | -1.74 | 0.000076 | 0.039331 |
| OR5B17 | Olfactory receptor, family 5, subfamily B, member 17 | -1.76 | 0.028114 | 0.299689 |
| OR5K2 | Olfactory receptor, family 5, subfamily K, member 2 | -1.81 | 0.008826 | 0.194658 |
| OSBPL10 | Oxysterol binding protein-like 10 | -1.53 | 0.010058 | 0.205061 |
| PA2G4P4 | Proliferation-associated 2G4 pseudogene 4 | -1.64 | 0.016907 | 0.247884 |
| PLAU | Plasminogen activator, urokinase | -2.79 | 0.000042 | 0.036741 |
| PLEKHG4B | Pleckstrin homology domain containing, family G (with rhogef domain) member 4B | -1.51 | 0.00095 | 0.087807 |
| PRKXP1 | Protein kinase, X-linked, pseudogene 1 | -1.6 | 0.002547 | 0.11979 |
| PSG2 | Pregnancy specific beta-1-glycoprotein 2 | -1.68 | 0.045003 | 0.35668 |
| PSG5 | Pregnancy specific beta-1-glycoprotein 5 | -1.53 | 0.007263 | 0.181459 |
| RFFL | Ring finger and FYVE-like domain containing E3 ubiquitin protein ligase | -1.54 | 0.000442 | 0.0668 |
| RIPK4 | Receptor-interacting serine-threonine kinase 4 | -1.54 | 0.00173 | 0.103251 |
| RN5S60 | RNA, 5S ribosomal 60 | -1.71 | 0.008098 | 0.18853 |
| RRS1 | RRS1 ribosome biogenesis regulator homolog (S. Cerevisiae) | -1.51 | 0.000678 | 0.076367 |
| RUNX1-IT1 | RUNX1 intronic transcript 1 (non-protein coding) | -1.71 | 0.000666 | 0.076367 |
| RUNX2 | Runt-related transcription factor 2 | -2.05 | 0.000026 | 0.031824 |
| SERPINB2, SERPINB10 | Serpin peptidase inhibitor, clade B (ovalbumin), member 2, serpin peptidase inhibitor, clade B (ovalbumin), member 10 | -2.87 | 0.000004 | 0.014639 |
| SHISA2 | Shisa homolog 2 (Xenopus laevis) | -2.72 | 0.00001 | 0.022705 |
| SKIL | SKI-like oncogene | -1.6 | 0.000839 | 0.084231 |
| SLC25A53 | Solute carrier family 25, member 53 | -1.56 | 0.01334 | 0.230327 |
| SLC7A6 | Solute carrier family 7 (amino acid transporter light chain, y+L system), member 6 | -1.55 | 0.000007 | 0.018228 |
| SLITRK6 | SLIT and NTRK-like family, member 6 | -1.65 | 0.010416 | 0.207063 |
| SMAD6 | SMAD family member 6 | -2 | 0.000071 | 0.039062 |
| SMAD7 | SMAD family member 7 | -2.24 | 0.00039 | 0.066035 |
| SMYD3-IT1 | SMYD3 intronic transcript 1 (non-protein coding) | -1.56 | 0.002265 | 0.114735 |
| SNAI2 | Snail homolog 2 (Drosophila) | -1.59 | 0.000053 | 0.039062 |
| SNORA55 | Small nucleolar RNA, H/ACA box 55 | -1.51 | 0.006036 | 0.168394 |
| SNORA71B, LOC388796 | Small nucleolar RNA, H/ACA box 71B, uncharacterized LOC388796 | -1.69 | 0.047522 | 0.363268 |
| SNORD114-31 | Small nucleolar RNA, C/D box 114-31 | -1.7 | 0.047841 | 0.363946 |
| SNORD52, C6orf48 | Small nucleolar RNA, C/D box 52, chromosome 6 open reading frame 48 | -1.51 | 0.003972 | 0.143222 |
| SNRPN, LOC100506948, SNORD116-28, SNORD115-26, SNORD115-13, SNORD115-7, SNORD107 | Small nuclear ribonucleoprotein polypeptide N, uncharacterized LOC100506948, small nucleolar RNA, C/D box 116-28, small nucleolar RNA, C/D box 115-26, small nucleolar RNA, C/D box 115-13, small nucleolar RNA, C/D box 115-7, small nucleolar RNA, C/D box 107 | -1.52 | 0.005048 | 0.158306 |
| STC2 | Stanniocalcin 2 | -1.69 | 0.000084 | 0.03948 |
| TFRC | Transferrin receptor (p90, CD71) | -1.58 | 0.000166 | 0.048851 |
| TIPARP | TCDD-inducible poly(ADP-ribose) polymerase | -2.03 | 0.000007 | 0.018327 |
| TIPIN | TIMELESS interacting protein | -1.58 | 0.007766 | 0.18557 |
| TMEM156 | Transmembrane protein 156 | -1.61 | 0.006675 | 0.175961 |
| TNFSF15 | Tumor necrosis factor (ligand) superfamily, member 15 | -1.56 | 0.007861 | 0.18654 |
| TRMT6 | Trna methyltransferase 6 homolog (S. Cerevisiae) | -1.81 | 0.000065 | 0.039062 |
| TRNAI6 | Transfer RNA isoleucine 6 (anticodon UAU) | -1.99 | 0.000917 | 0.086395 |
| YWHAH | Tyrosine 3-monooxygenase/tryptophan 5-monooxygenase activation protein, eta polypeptide | -1.54 | 0.00164 | 0.10135 |
| ZSWIM4 | Zinc finger, SWIM-type containing 4 | -1.88 | 0.004442 | 0.149054 |
| ***Up-regulated genes*** | | | | |
| ADAM20P1 | ADAM metallopeptidase domain 20 pseudogene 1 | 1.61 | 0.002329 | 0.115679 |
| ADAM32 | ADAM metallopeptidase domain 32 | 1.58 | 0.011572 | 0.215963 |
| AGBL5-AS1 | AGBL5 antisense RNA 1 (non-protein coding) | 2.68 | 0.002676 | 0.122494 |
| AHSA1 | AHA1, activator of heat shock 90kda protein atpase homolog 1 (yeast) | 1.52 | 0.000283 | 0.059647 |
| AKAP13 | A kinase (PRKA) anchor protein 13 | 1.86 | 0.000049 | 0.038344 |
| ALOX12P2 | Arachidonate 12-lipoxygenase pseudogene 2 | 1.86 | 0.000914 | 0.086395 |
| ANKUB1 | Ankyrin repeat and ubiquitin domain containing 1 | 1.83 | 0.001731 | 0.103251 |
| AQPEP | Laeverin | 1.65 | 0.01378 | 0.232486 |
| ARL17A, ARL17B, LOC100294341, LOC100506214 | ADP-ribosylation factor-like 17A, ADP-ribosylation factor-like 17B, ADP-ribosylation factor-like protein 17-like, uncharacterized LOC100506214 | 2.13 | 9.25E-07 | 0.0062 |
| BAG3 | BCL2-associated athanogene 3 | 2.12 | 0.000021 | 0.030243 |
| BMP4 | Bone morphogenetic protein 4 | 1.51 | 0.000047 | 0.03752 |
| BRI3 | Brain protein I3 | 1.72 | 0.004041 | 0.144164 |
| C14orf178 | Chromosome 14 open reading frame 178 | 1.73 | 0.016529 | 0.245766 |
| C16orf46 | Chromosome 16 open reading frame 46 | 1.59 | 0.021922 | 0.272424 |
| C17orf67 | Chromosome 17 open reading frame 67 | 1.56 | 0.001961 | 0.108295 |
| C6orf130 | Chromosome 6 open reading frame 130 | 1.51 | 0.016836 | 0.247524 |
| C7orf53 | Chromosome 7 open reading frame 53 | 1.54 | 0.002526 | 0.119179 |
| CALML4 | Calmodulin-like 4 | 1.89 | 0.00112 | 0.089547 |
| CAMTA1-IT1 | CAMTA1 intronic transcript 1 (non-protein coding) | 1.63 | 0.01454 | 0.236247 |
| CATSPER3, PCBD2 | Cation channel, sperm associated 3, pterin-4 alpha-carbinolamine dehydratase/dimerization cofactor of hepatocyte nuclear factor 1 alpha (TCF1) 2 | 1.72 | 0.013833 | 0.232572 |
| CCDC146 | Coiled-coil domain containing 146 | 1.76 | 0.00628 | 0.171186 |
| CEBPD | CCAAT/enhancer binding protein (C/EBP), delta | 1.66 | 0.000116 | 0.044031 |
| CHORDC1 | Cysteine and histidine-rich domain (CHORD) containing 1 | 2.02 | 0.000118 | 0.044031 |
| COPG2IT1 | COPG2 imprinted transcript 1 (non-protein coding) | 2.56 | 0.000868 | 0.085407 |
| CRYBB2P1 | Crystallin, beta B2 pseudogene 1 | 1.79 | 0.006906 | 0.178238 |
| CT45A5, CT45A2, CT45A3, CT45A6, CT45A4, CT45A1 | Cancer/testis antigen family 45, member A5, cancer/testis antigen family 45, member A2, cancer/testis antigen family 45, member A3, cancer/testis antigen family 45, member A6, cancer/testis antigen family 45, member A4, cancer/testis antigen family 45, member A1 | 1.54 | 0.021915 | 0.272424 |
| CUTC | Cutc copper transporter homolog (E. Coli) | 1.58 | 0.025384 | 0.288419 |
| DDIT4 | DNA-damage-inducible transcript 4 | 2.15 | 0.00044 | 0.0668 |
| DEDD2 | Death effector domain containing 2 | 1.64 | 0.000177 | 0.050844 |
| DIRAS3 | DIRAS family, GTP-binding RAS-like 3 | 1.53 | 0.007302 | 0.181728 |
| DNAJA4 | Dnaj (Hsp40) homolog, subfamily A, member 4 | 2.29 | 0.000082 | 0.039331 |
| DNAJB1 | Dnaj (Hsp40) homolog, subfamily B, member 1 | 1.95 | 0.000006 | 0.017829 |
| DRP2 | Dystrophin related protein 2 | 2.03 | 0.005808 | 0.165325 |
| DSEL | Dermatan sulfate epimerase-like | 1.79 | 0.000156 | 0.048316 |
| EDEM2, MT1P3 | ER degradation enhancer, mannosidase alpha-like 2, metallothionein 1 pseudogene 3 | 1.51 | 0.002326 | 0.115668 |
| FAM182A | Family with sequence similarity 182, member A | 1.9 | 0.000122 | 0.044372 |
| FAM182B | Family with sequence similarity 182, member B | 1.83 | 0.000146 | 0.047505 |
| FAM72C | Family with sequence similarity 72, member C | 2.22 | 0.000296 | 0.060797 |
| FBXO36 | F-box protein 36 | 1.82 | 0.000659 | 0.076367 |
| FCGR1B, FCGR1C | Fc fragment of igg, high affinity Ib, receptor (CD64), Fc fragment of igg, high affinity Ic, receptor (CD64), pseudogene | 1.66 | 0.037157 | 0.330506 |
| GATS | GATS, stromal antigen 3 opposite strand | 1.52 | 0.000479 | 0.068546 |
| GCNT4 | Glucosaminyl (N-acetyl) transferase 4, core 2 | 1.63 | 0.002929 | 0.12704 |
| GLYATL2 | Glycine-N-acyltransferase-like 2 | 1.86 | 0.005845 | 0.165816 |
| GSDMB | Gasdermin B | 1.66 | 0.001402 | 0.095855 |
| GTF2IRD2P1 | GTF2I repeat domain containing 2 pseudogene 1 | 1.51 | 0.002259 | 0.114735 |
| H2BFXP | H2B histone family, member X, pseudogene | 1.6 | 0.013663 | 0.231541 |
| H6PD | Hexose-6-phosphate dehydrogenase (glucose 1-dehydrogenase) | 1.58 | 0.005261 | 0.159983 |
| HCG27 | HLA complex group 27 (non-protein coding) | 1.78 | 0.029322 | 0.304089 |
| HSP90AB3P | Heat shock protein 90kda alpha (cytosolic), class B member 3, pseudogene | 1.62 | 0.002589 | 0.120363 |
| HSPA1B, HSPA1A | Heat shock 70kda protein 1B, heat shock 70kda protein 1A | 3.78 | 7.87E-07 | 0.006028 |
| HSPA1L | Heat shock 70kda protein 1-like | 1.52 | 0.015554 | 0.241502 |
| HSPA4L | Heat shock 70kda protein 4-like | 2.17 | 0.000017 | 0.026829 |
| HSPH1 | Heat shock 105kda/110kda protein 1 | 1.71 | 0.000029 | 0.032556 |
| IGHV4-31, IGHG1, IGHA1, IGH@, IGHJ2 | Immunoglobulin heavy variable 4-31, immunoglobulin heavy constant gamma 1 (G1m marker), immunoglobulin heavy constant alpha 1, immunoglobulin heavy locus, immunoglobulin heavy joining 2 | 1.64 | 0.001064 | 0.08889 |
| IL37 | Interleukin 37 | 1.53 | 0.008126 | 0.18886 |
| JAG1 | Jagged 1 | 1.51 | 0.000132 | 0.04663 |
| KIRREL3 | Kin of IRRE like 3 (Drosophila) | 1.66 | 0.000066 | 0.039062 |
| KLHDC1 | Kelch domain containing 1 | 1.59 | 0.000095 | 0.041936 |
| KRTAP6-3 | Keratin associated protein 6-3 | 1.71 | 0.006078 | 0.168512 |
| LHPP | Phospholysine phosphohistidine inorganic pyrophosphate phosphatase | 1.59 | 0.000143 | 0.047482 |
| LINC00310 | Long intergenic non-protein coding RNA 310 | 1.98 | 0.000069 | 0.039062 |
| LINC00473 | Long intergenic non-protein coding RNA 473 | 2.29 | 0.000064 | 0.039062 |
| LMOD1 | Leiomodin 1 (smooth muscle) | 1.51 | 0.010945 | 0.211566 |
| LOC100129033 | Qiqn5815 | 1.59 | 0.00783 | 0.186506 |
| LOC100132167 | Uncharacterized LOC100132167 | 1.97 | 0.000221 | 0.054688 |
| LOC100289187, ZNF655 | Transmembrane protein 225-like, zinc finger protein 655 | 1.51 | 0.0045 | 0.14985 |
| LOC100293962 | Uncharacterized LOC100293962 | 1.83 | 0.000901 | 0.086165 |
| LOC100506124, TTC21B | Uncharacterized LOC100506124, tetratricopeptide repeat domain 21B | 1.67 | 0.01877 | 0.256827 |
| LOC100506136 | Uncharacterized LOC100506136 | 1.83 | 0.012027 | 0.219409 |
| LOC100506252 | Uncharacterized LOC100506252 | 2.22 | 0.010567 | 0.208532 |
| LOC100506314 | Uncharacterized LOC100506314 | 1.52 | 0.010195 | 0.205877 |
| LOC100506380, C7orf13 | Uncharacterized LOC100506380, chromosome 7 open reading frame 13 | 1.66 | 0.041591 | 0.345463 |
| LOC100506714 | Uncharacterized LOC100506714 | 2.01 | 0.003887 | 0.142318 |
| LOC100507322, LOC645513 | Uncharacterized LOC100507322, uncharacterized LOC645513 | 1.74 | 0.015345 | 0.240225 |
| LOC100507424, ITFG2 | Uncharacterized LOC100507424, integrin alpha FG-GAP repeat containing 2 | 1.54 | 0.012774 | 0.226033 |
| LOC100508633 | Uncharacterized LOC100508633 | 1.92 | 0.010673 | 0.209704 |
| LOC100652943 | Putative uncharacterized protein FLJ38264-like | 1.59 | 0.034385 | 0.322987 |
| LOC100653017, MIR612, NEAT1 | Uncharacterized LOC100653017, microrna 612, nuclear paraspeckle assembly transcript 1 (non-protein coding) | 1.52 | 0.005156 | 0.159438 |
| LOC143188 | Uncharacterized LOC143188 | 1.51 | 0.003874 | 0.142318 |
| LOC283788 | FSHD region gene 1 pseudogene | 1.67 | 0.007708 | 0.184974 |
| LOC401324 | Uncharacterized LOC401324 | 1.55 | 0.039073 | 0.337051 |
| LOC730098 | Uncharacterized LOC730098 | 1.86 | 0.003185 | 0.130933 |
| LPIN2, LOC727896, CHORDC1 | Lipin 2, cysteine and histidine-rich domain (CHORD) containing 1 pseudogene, cysteine and histidine-rich domain (CHORD) containing 1 | 1.68 | 0.000006 | 0.017829 |
| LRRC39 | Leucine rich repeat containing 39 | 1.81 | 0.002979 | 0.128354 |
| MGC39372 | Serpin peptidase inhibitor, clade B (ovalbumin), member 9 pseudogene | 1.54 | 0.033361 | 0.319271 |
| MINOS1P1 | Mitochondrial inner membrane organizing system 1 pseudogene 1 | 1.93 | 0.005215 | 0.159826 |
| MIR188 | Microrna 188 | 1.86 | 0.03453 | 0.323497 |
| MIR1972-1, MIR1972-2 | Microrna 1972-1, microrna 1972-2 | 1.64 | 0.004747 | 0.153288 |
| MIR3132 | Microrna 3132 | 1.69 | 0.002555 | 0.12002 |
| MIR3619 | Microrna 3619 | 1.68 | 0.003873 | 0.142318 |
| MIR548B | Microrna 548b | 1.54 | 0.00825 | 0.189754 |
| MT1F | Metallothionein 1F | 2.05 | 0.000387 | 0.066013 |
| MT1P3 | Metallothionein 1 pseudogene 3 | 1.73 | 0.000289 | 0.060146 |
| N4BP2L1 | NEDD4 binding protein 2-like 1 | 1.54 | 0.011571 | 0.215963 |
| NOXRED1 | NADP-dependent oxidoreductase domain containing 1 | 1.61 | 0.022478 | 0.275206 |
| NR4A3 | Nuclear receptor subfamily 4, group A, member 3 | 1.62 | 0.001356 | 0.095061 |
| PAK3 | P21 protein (Cdc42/Rac)-activated kinase 3 | 1.63 | 0.005879 | 0.166217 |
| PARD6G-AS1 | PARD6G antisense RNA 1 (non-protein coding) | 2.25 | 0.000072 | 0.039062 |
| PER1 | Period homolog 1 (Drosophila) | 1.69 | 0.005493 | 0.16253 |
| PEX5 | Peroxisomal biogenesis factor 5 | 1.74 | 0.000202 | 0.052429 |
| PHEX | Phosphate regulating endopeptidase homolog, X-linked | 1.58 | 0.000114 | 0.043991 |
| PIGZ | Phosphatidylinositol glycan anchor biosynthesis, class Z | 1.57 | 0.000507 | 0.069867 |
| POLN | Polymerase (DNA directed) nu | 1.85 | 0.000875 | 0.085407 |
| POM121L6P | POM121 transmembrane nucleoporin-like 6 pseudogene | 1.6 | 0.037294 | 0.330848 |
| RAB28 | RAB28, member RAS oncogene family | 1.58 | 0.018585 | 0.256626 |
| RABGEF1 | RAB guanine nucleotide exchange factor (GEF) 1 | 1.51 | 0.003087 | 0.130029 |
| RASA4B, RASA4 | RAS p21 protein activator 4B, RAS p21 protein activator 4 | 1.72 | 0.002506 | 0.119031 |
| RCN1, DKFZp686K1684, TPT1-AS1 | Reticulocalbin 1, EF-hand calcium binding domain, uncharacterized LOC440034, TPT1 antisense RNA 1 (non-protein coding) | 1.58 | 0.00014 | 0.047283 |
| RECK | Reversion-inducing-cysteine-rich protein with kazal motifs | 1.59 | 0.000468 | 0.068309 |
| RN5S153 | RNA, 5S ribosomal 153 | 1.64 | 0.004345 | 0.147998 |
| RN5S511 | RNA, 5S ribosomal 511 | 1.76 | 0.022242 | 0.274216 |
| RNU7-24P | RNA, U7 small nuclear 24 pseudogene | 1.56 | 0.00713 | 0.180401 |
| SAT1 | Spermidine/spermine N1-acetyltransferase 1 | 1.78 | 0.000433 | 0.0668 |
| SDIM1 | Stress responsive DNAJB4 interacting membrane protein 1 | 2.63 | 0.000408 | 0.0668 |
| SEC31B | SEC31 homolog B (S. Cerevisiae) | 1.57 | 0.002855 | 0.125927 |
| SLC15A2 | Solute carrier family 15 (H+/peptide transporter), member 2 | 1.58 | 0.026862 | 0.294229 |
| SLFN5 | Schlafen family member 5 | 1.69 | 0.000049 | 0.038281 |
| SMAD9-AS1 | SMAD9 antisense RNA 1 (non-protein coding) | 1.81 | 0.034121 | 0.321921 |
| SMOX | Spermine oxidase | 1.59 | 0.000067 | 0.039062 |
| SNORD14E | Small nucleolar RNA, C/D box 14E | 1.6 | 0.005529 | 0.162834 |
| STL | Six-twelve leukemia | 1.55 | 0.020806 | 0.266498 |
| THOC6 | THO complex 6 homolog (Drosophila) | 1.58 | 0.000418 | 0.0668 |
| TMEM187 | Transmembrane protein 187 | 1.54 | 0.000275 | 0.05876 |
| TPT1-AS1 | TPT1 antisense RNA 1 (non-protein coding) | 1.58 | 0.000504 | 0.069867 |
| TPTE2P6 | Transmembrane phosphoinositide 3-phosphatase and tensin homolog 2 pseudogene 6 | 1.55 | 0.019276 | 0.257862 |
| TRANK1 | Tetratricopeptide repeat and ankyrin repeat containing 1 | 1.62 | 0.007993 | 0.187579 |
| ZFAND2A | Zinc finger, AN1-type domain 2A | 1.54 | 0.00037 | 0.065767 |
| ZNF117 | Zinc finger protein 117 | 1.52 | 0.003153 | 0.130684 |
| ZNF554 | Zinc finger protein 554 | 1.68 | 0.048674 | 0.366171 |
| ZNF710 | Zinc finger protein 710 | 1.62 | 0.03171 | 0.312943 |
