# Supplementary material for: Gene Expression Profile and Toxic Effects in Human Bronchial Epithelial Cells Exposed to Zearalenone
Source: PLoS One. 2014 May 2;9(5):e96404. doi: 10.1371/journal.pone.0096404 (PMC4008614; doi:10.1371/journal.pone.0096404)
Supplement: Table S3 — The complete list of differentially expressed genes (fold change >1.5, ANOVA p-value <0.05) in BEAS-2B cells after 24 h treatment. (DOCX) [file pone.0096404.s003.docx]

Table S3. The complete list of differentially expressed genes (fold change >1.5, ANOVA p-value <0.05) in BEAS-2B cells after 24h treatment with ZEA.

| **Gene Symbol** | **Gene name** | **Fold change** | **ANOVA p-value** | **FDR p-value** |
| --- | --- | --- | --- | --- |
| ***Up-regulated genes*** | | | | |
| ACBD7 | Acyl-coa binding domain containing 7 | 1.57 | 0.003534 | 0.065726 |
| ACSF2 | Acyl-coa synthetase family member 2 | 1.75 | 0.001003 | 0.035727 |
| ACSS3 | Acyl-coa synthetase short-chain family member 3 | 1.59 | 0.004915 | 0.078543 |
| ACTR3B | ARP3 actin-related protein 3 homolog B (yeast) | 1.55 | 0.001634 | 0.04479 |
| ADAM20P1 | ADAM metallopeptidase domain 20 pseudogene 1 | 1.61 | 0.002182 | 0.051846 |
| ADC | Arginine decarboxylase | 2.22 | 0.001297 | 0.040037 |
| ADHFE1 | Alcohol dehydrogenase, iron containing, 1 | 1.58 | 0.009021 | 0.106973 |
| AGBL5-AS1 | AGBL5 antisense RNA 1 (non-protein coding) | 4.11 | 0.000101 | 0.014213 |
| AHSA1 | AHA1, activator of heat shock 90kda protein atpase homolog 1 (yeast) | 2.47 | 0.000024 | 0.009862 |
| AKAP13 | A kinase (PRKA) anchor protein 13 | 1.82 | 0.005051 | 0.07953 |
| ALDH1L2 | Aldehyde dehydrogenase 1 family, member L2 | 2.55 | 0.000111 | 0.014264 |
| ALDH2 | Aldehyde dehydrogenase 2 family (mitochondrial) | 1.8 | 0.000554 | 0.027481 |
| ALDH6A1 | Aldehyde dehydrogenase 6 family, member A1 | 1.52 | 0.000646 | 0.029387 |
| ALOX12P2 | Arachidonate 12-lipoxygenase pseudogene 2 | 2.03 | 0.007136 | 0.094429 |
| ANAPC16 | Anaphase promoting complex subunit 16 | 1.6 | 0.000144 | 0.015601 |
| AOC2 | Amine oxidase, copper containing 2 (retina-specific) | 1.59 | 0.028866 | 0.202631 |
| APOBEC3D | Apolipoprotein B mrna editing enzyme, catalytic polypeptide-like 3D | 1.51 | 0.037012 | 0.23205 |
| APOLD1 | Apolipoprotein L domain containing 1 | 1.93 | 0.000105 | 0.014264 |
| ARL17A, ARL17B, LOC100294341, LOC100506214 | ADP-ribosylation factor-like 17A, ADP-ribosylation factor-like 17B, ADP-ribosylation factor-like protein 17-like, uncharacterized LOC100506214 | 2.13 | 0.00065 | 0.029387 |
| ASNS | Asparagine synthetase (glutamine-hydrolyzing) | 2.35 | 0.000109 | 0.014264 |
| ASNS, ASNSP1 | Asparagine synthetase (glutamine-hydrolyzing), asparagine synthetase pseudogene 1 | 1.94 | 0.000107 | 0.014264 |
| ASNS, ASNSP4 | Asparagine synthetase (glutamine-hydrolyzing), asparagine synthetase pseudogene 4 | 1.7 | 0.011758 | 0.124415 |
| ATE1 | Arginyltransferase 1 | 1.87 | 0.006807 | 0.091937 |
| ATP2C1 | Atpase, Ca++ transporting, type 2C, member 1 | 1.51 | 0.000076 | 0.013336 |
| AURKA | Aurora kinase A | 1.62 | 0.000088 | 0.013711 |
| BAG3 | BCL2-associated athanogene 3 | 1.91 | 0.000318 | 0.021942 |
| BCO2 | Beta-carotene oxygenase 2 | 2.42 | 0.000564 | 0.027655 |
| BFSP1 | Beaded filament structural protein 1, filensin | 1.68 | 0.021596 | 0.172484 |
| BIRC5 | Baculoviral IAP repeat containing 5 | 1.59 | 0.001291 | 0.040035 |
| BORA | Bora, aurora kinase A activator | 1.71 | 0.000205 | 0.018481 |
| BRI3 | Brain protein I3 | 1.61 | 0.005306 | 0.08133 |
| BST2 | Bone marrow stromal cell antigen 2 | 1.54 | 0.001362 | 0.040801 |
| BUB1 | Budding uninhibited by benzimidazoles 1 homolog (yeast) | 1.69 | 0.000029 | 0.009862 |
| C14orf45 | Chromosome 14 open reading frame 45 | 2.02 | 0.014895 | 0.140599 |
| C15orf23 | Chromosome 15 open reading frame 23 | 1.53 | 0.000227 | 0.019396 |
| C15orf48, MIR147B | Chromosome 15 open reading frame 48, microrna 147b | 1.59 | 0.004022 | 0.07075 |
| C16orf46 | Chromosome 16 open reading frame 46 | 1.63 | 0.008751 | 0.105389 |
| C17orf51, FAM27L | Chromosome 17 open reading frame 51, family with sequence similarity 27-like | 1.59 | 0.000235 | 0.019468 |
| C20orf132 | Chromosome 20 open reading frame 132 | 1.65 | 0.003131 | 0.061641 |
| C2orf15, MRPL30 | Chromosome 2 open reading frame 15, mitochondrial ribosomal protein L30 | 2.02 | 0.009684 | 0.111444 |
| C2orf48 | Chromosome 2 open reading frame 48 | 1.85 | 0.013524 | 0.133764 |
| C2orf63 | Chromosome 2 open reading frame 63 | 1.51 | 0.00964 | 0.111281 |
| C4orf22 | Chromosome 4 open reading frame 22 | 1.74 | 0.0275 | 0.197067 |
| C5orf27 | Chromosome 5 open reading frame 27 | 1.61 | 0.002422 | 0.054143 |
| C6orf130 | Chromosome 6 open reading frame 130 | 1.54 | 0.000904 | 0.034182 |
| C7orf53 | Chromosome 7 open reading frame 53 | 1.61 | 0.002539 | 0.05527 |
| C9orf100 | Chromosome 9 open reading frame 100 | 1.67 | 0.000319 | 0.022006 |
| C9orf153 | Chromosome 9 open reading frame 153 | 1.56 | 0.016028 | 0.146705 |
| CACYBP | Calcyclin binding protein | 1.56 | 0.000129 | 0.015018 |
| CASC1 | Cancer susceptibility candidate 1 | 1.66 | 0.012054 | 0.12623 |
| CCDC146 | Coiled-coil domain containing 146 | 2.52 | 0.000401 | 0.023863 |
| CCDC147 | Coiled-coil domain containing 147 | 2.02 | 0.002486 | 0.054762 |
| CCDC148 | Coiled-coil domain containing 148 | 1.87 | 0.003653 | 0.067175 |
| CCDC169-SOHLH2, SOHLH2, CCDC169 | CCDC169-SOHLH2 readthrough, spermatogenesis and oogenesis specific basic helix-loop-helix 2, coiled-coil domain containing 169 | 1.51 | 0.01953 | 0.163802 |
| CCDC81 | Coiled-coil domain containing 81 | 1.71 | 0.004097 | 0.071333 |
| CCDC87 | Coiled-coil domain containing 87 | 1.51 | 0.035792 | 0.227861 |
| CCL26 | Chemokine (C-C motif) ligand 26 | 1.7 | 0.002883 | 0.059066 |
| CCNB1 | Cyclin B1 | 1.6 | 0.000355 | 0.022958 |
| CDC20 | Cell division cycle 20 homolog (S. Cerevisiae) | 1.53 | 0.000027 | 0.009862 |
| CDCA2 | Cell division cycle associated 2 | 1.57 | 0.000561 | 0.027654 |
| CDCA3 | Cell division cycle associated 3 | 1.72 | 0.000704 | 0.030116 |
| CENPE | Centromere protein E, 312kda | 1.54 | 0.000084 | 0.013636 |
| CENPF | Centromere protein F, 350/400kda (mitosin) | 1.74 | 6.45E-07 | 0.003839 |
| CEP112 | Centrosomal protein 112kda | 1.54 | 0.005587 | 0.083532 |
| CHAC1 | Chac, cation transport regulator homolog 1 (E. Coli) | 1.92 | 0.001405 | 0.041505 |
| CHORDC1 | Cysteine and histidine-rich domain (CHORD) containing 1 | 2.96 | 0.000029 | 0.009862 |
| CKAP5 | Cytoskeleton associated protein 5 | 1.55 | 0.000009 | 0.008368 |
| CKS2 | CDC28 protein kinase regulatory subunit 2 | 1.57 | 0.000011 | 0.009308 |
| CLDN12 | Claudin 12 | 1.58 | 0.002602 | 0.055874 |
| CLDN12, CDK14 | Claudin 12, cyclin-dependent kinase 14 | 5.25 | 0.000738 | 0.030888 |
| CLDN15 | Claudin 15 | 1.6 | 0.000998 | 0.035638 |
| CLK3 | CDC-like kinase 3 | 1.68 | 0.000328 | 0.022252 |
| COX6B2 | Cytochrome c oxidase subunit vib polypeptide 2 (testis) | 1.81 | 0.002963 | 0.059695 |
| CPB2-AS1 | CPB2 antisense RNA 1 (non-protein coding) | 1.64 | 0.004728 | 0.076987 |
| CRAT | Carnitine O-acetyltransferase | 1.55 | 0.000283 | 0.020835 |
| CRYBB2P1 | Crystallin, beta B2 pseudogene 1 | 1.71 | 0.003046 | 0.060762 |
| CSNK1E | Casein kinase 1, epsilon | 1.71 | 0.034581 | 0.22309 |
| CTBS | Chitobiase, di-N-acetyl- | 1.54 | 0.00016 | 0.016621 |
| CTGF | Connective tissue growth factor | 1.51 | 0.001501 | 0.042819 |
| DAB1 | Disabled homolog 1 (Drosophila) | 1.59 | 0.025608 | 0.189641 |
| DBF4B | DBF4 homolog B (S. Cerevisiae) | 1.51 | 0.000037 | 0.010337 |
| DCAKD | Dephospho-coa kinase domain containing | 1.58 | 0.002254 | 0.052477 |
| DDIT4 | DNA-damage-inducible transcript 4 | 2.62 | 0.000017 | 0.009534 |
| DEDD2 | Death effector domain containing 2 | 1.89 | 0.000013 | 0.009534 |
| DEPDC1 | DEP domain containing 1 | 1.72 | 0.000108 | 0.014264 |
| DIRAS3 | DIRAS family, GTP-binding RAS-like 3 | 1.57 | 0.04138 | 0.246713 |
| DLGAP5 | Discs, large (Drosophila) homolog-associated protein 5 | 1.52 | 0.000095 | 0.013972 |
| DNAJA1 | Dnaj (Hsp40) homolog, subfamily A, member 1 | 1.55 | 0.000378 | 0.023599 |
| DNAJA4 | Dnaj (Hsp40) homolog, subfamily A, member 4 | 3.32 | 0.000045 | 0.011216 |
| DNAJB1 | Dnaj (Hsp40) homolog, subfamily B, member 1 | 2.25 | 0.000017 | 0.009534 |
| DNHD1, FXC1 | Dynein heavy chain domain 1, fracture callus 1 homolog (rat) | 1.66 | 0.001899 | 0.048662 |
| DOCK6 | Dedicator of cytokinesis 6 | 1.71 | 0.001391 | 0.041401 |
| DUSP5 | Dual specificity phosphatase 5 | 1.66 | 0.002983 | 0.060045 |
| DZIP3 | DAZ interacting protein 3, zinc finger | 2.09 | 0.000015 | 0.009534 |
| E2F5 | E2F transcription factor 5, p130-binding | 2 | 0.00065 | 0.029387 |
| EARS2 | Glutamyl-trna synthetase 2, mitochondrial (putative) | 1.81 | 0.00042 | 0.024326 |
| EDEM2, MT1P3 | ER degradation enhancer, mannosidase alpha-like 2, metallothionein 1 pseudogene 3 | 1.91 | 0.002513 | 0.055023 |
| EPPK1 | Epiplakin 1 | 1.53 | 0.012775 | 0.129946 |
| ERLEC1 | Endoplasmic reticulum lectin 1 | 1.59 | 0.000095 | 0.013933 |
| ERV3-1, ZNF117 | Endogenous retrovirus group 3, member 1, zinc finger protein 117 | 1.77 | 0.001091 | 0.03699 |
| ETV5 | Ets variant 5 | 1.86 | 0.000018 | 0.009534 |
| EVI2B | Ecotropic viral integration site 2B | 2.25 | 0.004708 | 0.076726 |
| FAM182A | Family with sequence similarity 182, member A | 1.72 | 0.001231 | 0.039077 |
| FAM182B | Family with sequence similarity 182, member B | 1.61 | 0.000254 | 0.019889 |
| FAM227A | Family with sequence similarity 227, member A | 1.88 | 0.000092 | 0.01372 |
| FAM72C | Family with sequence similarity 72, member C | 2.24 | 0.000095 | 0.013972 |
| FAM72D | Family with sequence similarity 72, member D | 2.33 | 0.00011 | 0.014264 |
| FBLIM1 | Filamin binding LIM protein 1 | 1.81 | 0.000362 | 0.023103 |
| FBXO17, SARS2 | F-box protein 17, seryl-trna synthetase 2, mitochondrial | 1.58 | 0.002099 | 0.050937 |
| FBXO36 | F-box protein 36 | 1.67 | 0.00256 | 0.05536 |
| FBXO44 | F-box protein 44 | 1.67 | 0.001528 | 0.042991 |
| FBXW8 | F-box and WD repeat domain containing 8 | 1.59 | 0.00007 | 0.013149 |
| FCER1G | Fc fragment of ige, high affinity I, receptor for; gamma polypeptide | 1.77 | 0.004196 | 0.072319 |
| FER1L4 | Fer-1-like 4 (C. Elegans) pseudogene | 2.09 | 0.000324 | 0.022134 |
| FERMT3 | Fermitin family member 3 | 1.59 | 0.009113 | 0.107668 |
| FKBP4 | FK506 binding protein 4, 59kda | 2.11 | 0.000067 | 0.012906 |
| FKBP7 | FK506 binding protein 7 | 1.57 | 0.001925 | 0.048896 |
| FLJ38668 | Uncharacterized LOC644903 | 2.07 | 0.002926 | 0.059377 |
| FLJ42627 | Uncharacterized LOC645644 | 1.63 | 0.014242 | 0.137418 |
| FLJ45513 | Uncharacterized LOC729220 | 1.68 | 0.003699 | 0.067549 |
| GAS2L3 | Growth arrest-specific 2 like 3 | 1.57 | 0.000039 | 0.010667 |
| GATS | GATS, stromal antigen 3 opposite strand | 1.51 | 0.010156 | 0.11449 |
| GCAT | Glycine C-acetyltransferase | 1.61 | 0.002105 | 0.050938 |
| GINS4 | GINS complex subunit 4 (Sld5 homolog) | 1.78 | 0.000749 | 0.031008 |
| GOSR2 | Golgi SNAP receptor complex member 2 | 1.52 | 0.000239 | 0.01947 |
| GPX8 | Glutathione peroxidase 8 (putative) | 1.71 | 0.000096 | 0.013984 |
| GSDMB | Gasdermin B | 1.91 | 0.000028 | 0.009862 |
| GTF3C3 | General transcription factor IIIC, polypeptide 3, 102kda | 1.69 | 0.000027 | 0.009862 |
| HIBCH | 3-hydroxyisobutyryl-coa hydrolase | 1.69 | 0.000029 | 0.009862 |
| HIST1H2BN | Histone cluster 1, h2bn | 2.2 | 0.005559 | 0.083408 |
| HMBS | Hydroxymethylbilane synthase | 1.89 | 0.000056 | 0.012016 |
| HMGN2P46 | High mobility group nucleosomal binding domain 2 pseudogene 46 | 1.59 | 0.004755 | 0.077238 |
| HMMR | Hyaluronan-mediated motility receptor (RHAMM) | 1.68 | 0.000439 | 0.024845 |
| HSP90AA1 | Heat shock protein 90kda alpha (cytosolic), class A member 1 | 2.12 | 0.000306 | 0.021489 |
| HSP90AB1 | Heat shock protein 90kda alpha (cytosolic), class B member 1 | 1.76 | 0.004953 | 0.078634 |
| HSP90AB3P | Heat shock protein 90kda alpha (cytosolic), class B member 3, pseudogene | 1.88 | 0.006985 | 0.093424 |
| HSPA1B, HSPA1A | Heat shock 70kda protein 1B, heat shock 70kda protein 1A | 6.54 | 3.64E-08 | 0.000619 |
| HSPA1L | Heat shock 70kda protein 1-like | 1.56 | 0.005084 | 0.079644 |
| HSPA4 | Heat shock 70kda protein 4 | 1.65 | 0.000002 | 0.003839 |
| HSPA4L | Heat shock 70kda protein 4-like | 2.75 | 0.000013 | 0.009534 |
| HSPD1, HSPD1P1 | Heat shock 60kda protein 1 (chaperonin), heat shock 60kda protein 1 (chaperonin) pseudogene 1 | 1.67 | 0.000075 | 0.013336 |
| HSPH1 | Heat shock 105kda/110kda protein 1 | 2.66 | 0.000063 | 0.012645 |
| HYLS1 | Hydrolethalus syndrome 1 | 1.9 | 0.003987 | 0.070408 |
| IDNK | Idnk, gluconokinase homolog (E. Coli) | 1.69 | 0.01169 | 0.124144 |
| IFIT1 | Interferon-induced protein with tetratricopeptide repeats 1 | 2.29 | 0.006302 | 0.088484 |
| IL11 | Interleukin 11 | 2.1 | 0.000276 | 0.020677 |
| IL37 | Interleukin 37 | 1.77 | 0.00075 | 0.031024 |
| INA | Internexin neuronal intermediate filament protein, alpha | 1.76 | 0.002818 | 0.058199 |
| INCENP | Inner centromere protein antigens 135/155kda | 1.54 | 0.000052 | 0.011748 |
| INTS2 | Integrator complex subunit 2 | 1.61 | 0.000209 | 0.018642 |
| IQCK | IQ motif containing K | 1.67 | 0.003104 | 0.061304 |
| ISYNA1 | Inositol-3-phosphate synthase 1 | 1.78 | 0.001894 | 0.048613 |
| ITGB3 | Integrin, beta 3 (platelet glycoprotein iiia, antigen CD61) | 1.95 | 0.000081 | 0.013636 |
| ITPRIP | Inositol 1,4,5-trisphosphate receptor interacting protein | 1.63 | 0.000248 | 0.019709 |
| IVD | Isovaleryl-coa dehydrogenase | 1.56 | 0.000115 | 0.014389 |
| JMJD6 | Jumonji domain containing 6 | 1.73 | 0.000016 | 0.009534 |
| KCNG1 | Potassium voltage-gated channel, subfamily G, member 1 | 1.6 | 0.013588 | 0.134099 |
| KGFLP2 | Keratinocyte growth factor-like protein 2 | 1.57 | 0.029318 | 0.204333 |
| KIAA1919 | Kiaa1919 | 1.7 | 0.000001 | 0.003839 |
| KIF14 | Kinesin family member 14 | 1.55 | 0.000046 | 0.011381 |
| KIF18A | Kinesin family member 18A | 1.59 | 0.000094 | 0.013857 |
| KIF2C | Kinesin family member 2C | 1.76 | 0.000131 | 0.015116 |
| KLHDC1 | Kelch domain containing 1 | 2 | 0.001028 | 0.035964 |
| KRCC1 | Lysine-rich coiled-coil 1 | 1.53 | 0.000191 | 0.017826 |
| LCORL | Ligand dependent nuclear receptor corepressor-like | 1.75 | 0.000016 | 0.009534 |
| LHPP | Phospholysine phosphohistidine inorganic pyrophosphate phosphatase | 1.91 | 0.001052 | 0.036399 |
| LINC00277 | Long intergenic non-protein coding RNA 277 | 1.81 | 0.007211 | 0.094891 |
| LINC00310 | Long intergenic non-protein coding RNA 310 | 1.71 | 0.000346 | 0.022814 |
| LINC00473 | Long intergenic non-protein coding RNA 473 | 2.04 | 0.000798 | 0.032024 |
| LMOD1 | Leiomodin 1 (smooth muscle) | 3.91 | 0.000049 | 0.011594 |
| LNP1 | Leukemia NUP98 fusion partner 1 | 1.97 | 0.005972 | 0.086356 |
| LOC100130157 | Uncharacterized LOC100130157 | 2.28 | 0.000406 | 0.023903 |
| LOC100130950 | Uncharacterized LOC100130950 | 1.53 | 0.001497 | 0.042703 |
| LOC100132057 | Phosphodiesterase 4D interacting protein pseudogene | 2.01 | 0.020319 | 0.167078 |
| LOC100132167 | Uncharacterized LOC100132167 | 2.01 | 0.000098 | 0.014099 |
| LOC100287765 | Uncharacterized LOC100287765 | 1.59 | 0.044821 | 0.257732 |
| LOC100288637 | OTU domain containing 7A pseudogene | 1.69 | 0.000683 | 0.029751 |
| LOC100289187, ZNF655 | Transmembrane protein 225-like, zinc finger protein 655 | 1.92 | 0.00048 | 0.025637 |
| LOC100293962 | Uncharacterized LOC100293962 | 4.01 | 0.000045 | 0.011216 |
| LOC100505536 | Uncharacterized LOC100505536 | 1.61 | 0.006935 | 0.092982 |
| LOC100505769 | Uncharacterized LOC100505769 | 1.67 | 0.002103 | 0.050937 |
| LOC100506338 | Uncharacterized LOC100506338 | 2.24 | 0.000562 | 0.027654 |
| LOC100506459 | Uncharacterized LOC100506459 | 1.52 | 0.023219 | 0.179538 |
| LOC100506901 | Uncharacterized LOC100506901 | 1.63 | 0.00008 | 0.013636 |
| LOC100506992 | Uncharacterized LOC100506992 | 2.36 | 0.007966 | 0.100244 |
| LOC100507032 | Uncharacterized LOC100507032 | 1.51 | 0.004947 | 0.078632 |
| LOC100507322, LOC645513 | Uncharacterized LOC100507322, uncharacterized LOC645513 | 1.92 | 0.015714 | 0.144944 |
| LOC100507367 | Uncharacterized LOC100507367 | 1.99 | 0.000064 | 0.012645 |
| LOC100507424, ITFG2 | Uncharacterized LOC100507424, integrin alpha FG-GAP repeat containing 2 | 1.69 | 0.004139 | 0.071779 |
| LOC100507539 | Uncharacterized LOC100507539 | 1.57 | 0.031428 | 0.2126 |
| LOC100652768, LOC100653185 | Uncharacterized LOC100652768, uncharacterized LOC100653185 | 1.59 | 0.003304 | 0.0634 |
| LOC100652943 | Putative uncharacterized protein FLJ38264-like | 1.77 | 0.025804 | 0.190336 |
| LOC143188 | Uncharacterized LOC143188 | 1.52 | 0.01573 | 0.14501 |
| LOC221272 | Uncharacterized LOC221272 | 1.75 | 0.000104 | 0.014213 |
| LOC338739 | Uncharacterized LOC338739 | 1.64 | 0.002632 | 0.056068 |
| LOC440993 | Uncharacterized LOC440993 | 1.96 | 0.0023 | 0.052942 |
| LOC646762 | Uncharacterized LOC646762 | 1.56 | 0.006012 | 0.086557 |
| LOC728228 | Uncharacterized LOC728228 | 1.69 | 0.000592 | 0.028347 |
| LOC728855, LOC728875, LOC100293748 | Uncharacterized LOC728855, uncharacterized LOC728875, putative neuroblastoma breakpoint family member 6-like protein-like | 1.59 | 0.024355 | 0.185148 |
| LOC730098 | Uncharacterized LOC730098 | 2.41 | 0.001309 | 0.040128 |
| LPIN2, LOC727896, CHORDC1 | Lipin 2, cysteine and histidine-rich domain (CHORD) containing 1 pseudogene, cysteine and histidine-rich domain (CHORD) containing 1 | 2.11 | 0.000053 | 0.011837 |
| LRP4-AS1 | LRP4 antisense RNA 1 (non-protein coding) | 2.95 | 0.00208 | 0.050775 |
| LRRC41 | Leucine rich repeat containing 41 | 1.61 | 0.000434 | 0.024665 |
| MBTPS2 | Membrane-bound transcription factor peptidase, site 2 | 1.55 | 0.000687 | 0.02985 |
| MEF2D | Myocyte enhancer factor 2D | 1.59 | 0.000341 | 0.022619 |
| MERTK | C-mer proto-oncogene tyrosine kinase | 1.79 | 0.001181 | 0.038301 |
| METTL10 | Methyltransferase like 10 | 1.56 | 0.000247 | 0.019709 |
| METTL12 | Methyltransferase like 12 | 2.08 | 0.001211 | 0.038778 |
| MFAP3, PCNXL2 | Microfibrillar-associated protein 3, pecanex-like 2 (Drosophila) | 1.64 | 0.006465 | 0.08962 |
| MFSD2A | Major facilitator superfamily domain containing 2A | 2 | 0.001606 | 0.044385 |
| MGC11082 | Uncharacterized LOC84777 | 1.71 | 0.017006 | 0.151895 |
| MGC39372 | Serpin peptidase inhibitor, clade B (ovalbumin), member 9 pseudogene | 1.61 | 0.002226 | 0.052102 |
| MIR3132 | Microrna 3132 | 2.24 | 0.00184 | 0.047907 |
| MIR3198-1 | Microrna 3198-1 | 1.54 | 0.027604 | 0.197485 |
| MIR4534 | Microrna 4534 | 1.97 | 0.002286 | 0.052855 |
| MIR613 | Microrna 613 | 2.02 | 0.003025 | 0.060538 |
| MKNK2 | MAP kinase interacting serine/threonine kinase 2 | 1.52 | 0.001968 | 0.04937 |
| MPZL2 | Myelin protein zero-like 2 | 1.71 | 0.001075 | 0.036852 |
| MSRB3 | Methionine sulfoxide reductase B3 | 1.6 | 0.002184 | 0.051854 |
| MT1F | Metallothionein 1F | 2.47 | 0.000019 | 0.009534 |
| MT1P3 | Metallothionein 1 pseudogene 3 | 2.12 | 0.000338 | 0.022619 |
| MYADM | Myeloid-associated differentiation marker | 1.87 | 0.004827 | 0.077886 |
| MYEF2 | Myelin expression factor 2 | 1.91 | 0.001284 | 0.04002 |
| NAT9 | N-acetyltransferase 9 (GCN5-related, putative) | 1.63 | 0.000563 | 0.027654 |
| NAV2 | Neuron navigator 2 | 1.69 | 0.000001 | 0.003839 |
| NAV2, NAV2-AS1 | Neuron navigator 2, NAV2 antisense RNA 1 (non-protein coding) | 1.85 | 0.009177 | 0.108199 |
| NEK2 | NIMA (never in mitosis gene a)-related kinase 2 | 2.17 | 0.000004 | 0.005162 |
| NEK9 | NIMA (never in mitosis gene a)- related kinase 9 | 1.52 | 0.0003 | 0.021228 |
| NIPAL1 | NIPA-like domain containing 1 | 1.73 | 0.00131 | 0.040128 |
| NOX5, SPESP1 | NADPH oxidase, EF-hand calcium binding domain 5, sperm equatorial segment protein 1 | 1.6 | 0.000136 | 0.0152 |
| NOXRED1 | NADP-dependent oxidoreductase domain containing 1 | 1.65 | 0.000327 | 0.022192 |
| NPAS4 | Neuronal PAS domain protein 4 | 1.71 | 0.012513 | 0.128489 |
| NR4A3 | Nuclear receptor subfamily 4, group A, member 3 | 5.79 | 0.000177 | 0.017322 |
| NRP2 | Neuropilin 2 | 1.59 | 0.002152 | 0.051591 |
| NUAK2 | NUAK family, SNF1-like kinase, 2 | 1.56 | 0.000082 | 0.013636 |
| NUF2 | NUF2, NDC80 kinetochore complex component, homolog (S. Cerevisiae) | 1.54 | 0.000002 | 0.003839 |
| ODF2 | Outer dense fiber of sperm tails 2 | 1.74 | 0.000042 | 0.010999 |
| OR2A7, OR2A4, LOC728377 | Olfactory receptor, family 2, subfamily A, member 7, olfactory receptor, family 2, subfamily A, member 4, Rho guanine nucleotide exchange factor (GEF) 5 pseudogene | 1.82 | 0.001496 | 0.042703 |
| OR2L2 | Olfactory receptor, family 2, subfamily L, member 2 | 1.7 | 0.015689 | 0.14485 |
| OR4F4, OR4F17 | Olfactory receptor, family 4, subfamily F, member 4, olfactory receptor, family 4, subfamily F, member 17 | 1.56 | 0.030485 | 0.20882 |
| OR8H2 | Olfactory receptor, family 8, subfamily H, member 2 | 1.53 | 0.006445 | 0.089453 |
| OSCP1 | Organic solute carrier partner 1 | 1.67 | 0.01632 | 0.148585 |
| P4HA2 | Prolyl 4-hydroxylase, alpha polypeptide II | 1.53 | 0.000026 | 0.009862 |
| PAK3 | P21 protein (Cdc42/Rac)-activated kinase 3 | 1.91 | 0.002338 | 0.053126 |
| PARD6G-AS1 | PARD6G antisense RNA 1 (non-protein coding) | 2.64 | 0.000303 | 0.021315 |
| PARPBP | PARP1 binding protein | 1.51 | 0.000153 | 0.016254 |
| PDE4D | Phosphodiesterase 4D, camp-specific | 1.81 | 0.000193 | 0.017845 |
| PDE7B | Phosphodiesterase 7B | 1.72 | 0.025147 | 0.187711 |
| PDGFRL | Platelet-derived growth factor receptor-like | 1.53 | 0.001912 | 0.048797 |
| PIM2 | Pim-2 oncogene | 1.58 | 0.000408 | 0.023956 |
| PLEKHA2 | Pleckstrin homology domain containing, family A (phosphoinositide binding specific) member 2 | 1.6 | 0.015176 | 0.141854 |
| PLOD1 | Procollagen-lysine, 2-oxoglutarate 5-dioxygenase 1 | 2.49 | 0.000645 | 0.029385 |
| PMCHL2 | Pro-melanin-concentrating hormone-like 2, pseudogene | 1.59 | 0.036876 | 0.231683 |
| POC5 | POC5 centriolar protein homolog (Chlamydomonas) | 1.6 | 0.001721 | 0.046062 |
| POLN | Polymerase (DNA directed) nu | 1.56 | 0.000372 | 0.023476 |
| POLR2J | Polymerase (RNA) II (DNA directed) polypeptide J, 13.3kda | 1.68 | 0.00047 | 0.025476 |
| POLR2J4 | Polymerase (RNA) II (DNA directed) polypeptide J4, pseudogene | 1.55 | 0.012428 | 0.127974 |
| POLR3E | Polymerase (RNA) III (DNA directed) polypeptide E (80kd) | 1.59 | 0.000382 | 0.023695 |
| PPID | Peptidylprolyl isomerase D | 1.93 | 0.000404 | 0.023895 |
| PPP1R32 | Protein phosphatase 1, regulatory subunit 32 | 1.54 | 0.009858 | 0.112271 |
| PRKD2 | Protein kinase D2 | 1.84 | 0.000086 | 0.013711 |
| PSAT1 | Phosphoserine aminotransferase 1 | 2.37 | 0.000002 | 0.003839 |
| PSMD7, LOC100506201 | Proteasome (prosome, macropain) 26S subunit, non-atpase, 7, uncharacterized LOC100506201 | 1.56 | 0.000809 | 0.032244 |
| PSRC1 | Proline/serine-rich coiled-coil 1 | 1.64 | 0.007129 | 0.094422 |
| PTGR2 | Prostaglandin reductase 2 | 1.56 | 0.013148 | 0.131798 |
| PTTG1 | Pituitary tumor-transforming 1 | 1.77 | 0.001175 | 0.038254 |
| RAB40A | RAB40A, member RAS oncogene family | 2.16 | 0.016799 | 0.151018 |
| RANGRF | RAN guanine nucleotide release factor | 1.56 | 0.000479 | 0.025637 |
| RASA4, RASA4CP, RASA4B, UPK3BL | RAS p21 protein activator 4, RAS p21 protein activator 4C, pseudogene, RAS p21 protein activator 4B, uroplakin 3B-like | 1.81 | 0.003789 | 0.068413 |
| RASA4B, RASA4 | RAS p21 protein activator 4B, RAS p21 protein activator 4 | 1.71 | 0.010012 | 0.113396 |
| RCN1, DKFZp686K1684, TPT1-AS1 | Reticulocalbin 1, EF-hand calcium binding domain, uncharacterized LOC440034, TPT1 antisense RNA 1 (non-protein coding) | 2.39 | 0.000058 | 0.012117 |
| RGS5 | Regulator of G-protein signaling 5 | 1.63 | 0.002318 | 0.052979 |
| RN5S153 | RNA, 5S ribosomal 153 | 1.77 | 0.017266 | 0.153168 |
| RN5S342, RN5S153, RN5S253, RN5S254 | RNA, 5S ribosomal 342, RNA, 5S ribosomal 153, RNA, 5S ribosomal 253, RNA, 5S ribosomal 254 | 2.19 | 0.036921 | 0.231803 |
| RNF26 | Ring finger protein 26 | 1.52 | 0.001337 | 0.040594 |
| RNFT2 | Ring finger protein, transmembrane 2 | 2.03 | 0.000272 | 0.020558 |
| RNU11 | RNA, U11 small nuclear | 1.82 | 0.003035 | 0.060614 |
| RNU4ATAC3P | RNA, u4atac small nuclear 3, pseudogene | 1.67 | 0.005195 | 0.080547 |
| RPGRIP1L | RPGRIP1-like | 1.62 | 0.000561 | 0.027654 |
| RPL28 | Ribosomal protein L28 | 1.6 | 0.000436 | 0.024665 |
| RPL31, TBC1D8 | Ribosomal protein L31, TBC1 domain family, member 8 (with GRAM domain) | 1.97 | 0.000314 | 0.021806 |
| RPL37, LOC100506548 | Ribosomal protein L37, uncharacterized LOC100506548 | 2.17 | 0.000024 | 0.009862 |
| RPL37A | Ribosomal protein l37a | 1.63 | 0.005906 | 0.085939 |
| RRP7B | Ribosomal RNA processing 7 homolog B (S. Cerevisiae) | 2.84 | 0.040061 | 0.242458 |
| RSBN1 | Round spermatid basic protein 1 | 1.55 | 0.006811 | 0.091945 |
| RUVBL2 | Ruvb-like 2 (E. Coli) | 1.54 | 0.000124 | 0.014763 |
| SDIM1 | Stress responsive DNAJB4 interacting membrane protein 1 | 2.79 | 0.000177 | 0.017322 |
| SEC24D | SEC24 family, member D (S. Cerevisiae) | 1.53 | 0.001849 | 0.047985 |
| SEC31B | SEC31 homolog B (S. Cerevisiae) | 1.73 | 0.001068 | 0.036789 |
| SERPINH1 | Serpin peptidase inhibitor, clade H (heat shock protein 47), member 1, (collagen binding protein 1) | 1.82 | 0.000896 | 0.034123 |
| SGOL1 | Shugoshin-like 1 (S. Pombe) | 1.56 | 0.001013 | 0.035859 |
| SIX4 | SIX homeobox 4 | 1.52 | 0.003523 | 0.06564 |
| SLC13A3 | Solute carrier family 13 (sodium-dependent dicarboxylate transporter), member 3 | 1.77 | 0.000667 | 0.029519 |
| SLC25A33 | Solute carrier family 25 (pyrimidine nucleotide carrier), member 33 | 1.51 | 0.001871 | 0.048264 |
| SLC25A4 | Solute carrier family 25 (mitochondrial carrier; adenine nucleotide translocator), member 4 | 1.59 | 0.000135 | 0.015156 |
| SLC6A14 | Solute carrier family 6 (amino acid transporter), member 14 | 1.52 | 0.008035 | 0.100721 |
| SLC7A11 | Solute carrier family 7 (anionic amino acid transporter light chain, xc- system), member 11 | 2.39 | 0.000001 | 0.003839 |
| SMOX | Spermine oxidase | 2.06 | 0.000026 | 0.009862 |
| SNORA74A | Small nucleolar RNA, H/ACA box 74A | 2.26 | 0.001581 | 0.043983 |
| SNORD12C | Small nucleolar RNA, C/D box 12C | 1.51 | 0.001161 | 0.038021 |
| SNORD14D, SNORD14C, HSPA8 | Small nucleolar RNA, C/D box 14D, small nucleolar RNA, C/D box 14C, heat shock 70kda protein 8 | 1.88 | 0.000929 | 0.034548 |
| SNORD14E | Small nucleolar RNA, C/D box 14E | 2.54 | 0.001536 | 0.043133 |
| SNORD18A, SNORD18C, SNORD18B, SNORD16, RPL4 | Small nucleolar RNA, C/D box 18A, small nucleolar RNA, C/D box 18C, small nucleolar RNA, C/D box 18B, small nucleolar RNA, C/D box 16, ribosomal protein L4 | 1.6 | 0.014537 | 0.138957 |
| SNORD38B, SNORD55, RPS8 | Small nucleolar RNA, C/D box 38B, small nucleolar RNA, C/D box 55, ribosomal protein S8 | 1.65 | 0.015451 | 0.143659 |
| SNORD92 | Small nucleolar RNA, C/D box 92 | 1.91 | 0.045979 | 0.261069 |
| SPA17 | Sperm autoantigenic protein 17 | 1.7 | 0.000613 | 0.02868 |
| SPPL2B | Signal peptide peptidase like 2B | 1.81 | 0.003459 | 0.065042 |
| SPR | Sepiapterin reductase (7,8-dihydrobiopterin:NADP+ oxidoreductase) | 2.21 | 0.000104 | 0.014213 |
| SPRY2 | Sprouty homolog 2 (Drosophila) | 1.8 | 0.000207 | 0.01853 |
| SRPX2 | Sushi-repeat containing protein, X-linked 2 | 1.62 | 0.000744 | 0.030888 |
| ST13 | Suppression of tumorigenicity 13 (colon carcinoma) (Hsp70 interacting protein) | 1.62 | 0.000045 | 0.011265 |
| ST13, LOC100653138 | Suppression of tumorigenicity 13 (colon carcinoma) (Hsp70 interacting protein), uncharacterized LOC100653138 | 1.67 | 0.012673 | 0.129432 |
| ST3GAL3 | ST3 beta-galactoside alpha-2,3-sialyltransferase 3 | 1.55 | 0.017747 | 0.155593 |
| STAG3L1 | Stromal antigen 3-like 1 | 1.52 | 0.020952 | 0.169596 |
| STIP1 | Stress-induced-phosphoprotein 1 | 1.81 | 0.000074 | 0.01331 |
| TAS2R3 | Taste receptor, type 2, member 3 | 1.72 | 0.001642 | 0.044845 |
| TAS2R4 | Taste receptor, type 2, member 4 | 2 | 0.004771 | 0.077334 |
| TAS2R5 | Taste receptor, type 2, member 5 | 2.35 | 0.000278 | 0.020687 |
| TFPI2 | Tissue factor pathway inhibitor 2 | 1.52 | 0.004086 | 0.071234 |
| TGM2 | Transglutaminase 2 (C polypeptide, protein-glutamine-gamma-glutamyltransferase) | 1.99 | 0.000732 | 0.030717 |
| TMEM106A | Transmembrane protein 106A | 1.57 | 0.009477 | 0.110196 |
| TMEM19 | Transmembrane protein 19 | 1.52 | 0.000081 | 0.013636 |
| TMEM91 | Transmembrane protein 91 | 1.87 | 0.000236 | 0.019468 |
| TNFAIP8L1 | Tumor necrosis factor, alpha-induced protein 8-like 1 | 1.88 | 0.000714 | 0.03039 |
| TPCN1 | Two pore segment channel 1 | 1.73 | 0.000269 | 0.020521 |
| TPT1-AS1 | TPT1 antisense RNA 1 (non-protein coding) | 2.21 | 0.000141 | 0.01546 |
| TRIM49B | Tripartite motif containing 49B | 1.56 | 0.020357 | 0.167276 |
| TRIM73 | Tripartite motif containing 73 | 1.71 | 0.001232 | 0.039077 |
| TRIM74, TRIM73 | Tripartite motif containing 74, tripartite motif containing 73 | 1.87 | 0.009398 | 0.109801 |
| TROAP | Trophinin associated protein (tastin) | 1.58 | 0.001241 | 0.039321 |
| TSPAN2 | Tetraspanin 2 | 1.85 | 0.002765 | 0.057594 |
| UBB | Ubiquitin B | 1.69 | 0.002126 | 0.051161 |
| UBE2C | Ubiquitin-conjugating enzyme E2C | 1.77 | 0.000163 | 0.016813 |
| UBE2S | Ubiquitin-conjugating enzyme E2S | 1.62 | 0.00529 | 0.081173 |
| UNC119 | Unc-119 homolog (C. Elegans) | 1.69 | 0.000442 | 0.02487 |
| USP11 | Ubiquitin specific peptidase 11 | 1.58 | 0.000171 | 0.017023 |
| VAMP4 | Vesicle-associated membrane protein 4 | 1.81 | 0.000039 | 0.010667 |
| WDR78 | WD repeat domain 78 | 1.81 | 0.000046 | 0.011271 |
| XPNPEP3 | X-prolyl aminopeptidase (aminopeptidase P) 3, putative | 1.79 | 0.000045 | 0.011216 |
| ZFAND2A | Zinc finger, AN1-type domain 2A | 1.74 | 0.000057 | 0.012117 |
| ZMYM1 | Zinc finger, MYM-type 1 | 1.53 | 0.00016 | 0.016621 |
| ZNF117 | Zinc finger protein 117 | 1.79 | 0.001173 | 0.038254 |
| ZNF473 | Zinc finger protein 473 | 1.77 | 0.000463 | 0.025367 |
| ZNF528 | Zinc finger protein 528 | 1.51 | 0.004254 | 0.072824 |
| ZNF554 | Zinc finger protein 554 | 1.51 | 0.022879 | 0.177915 |
| ZNF581 | Zinc finger protein 581 | 1.52 | 0.00411 | 0.071462 |
| ZNF710 | Zinc finger protein 710 | 2.01 | 0.00364 | 0.067015 |
| ZNF714 | Zinc finger protein 714 | 2.02 | 0.001189 | 0.038407 |
| ZNF737 | Zinc finger protein 737 | 1.82 | 0.007602 | 0.097844 |
| ZNF75A | Zinc finger protein 75a | 1.6 | 0.000359 | 0.023066 |
| ZNF773 | Zinc finger protein 773 | 1.55 | 0.001303 | 0.040048 |
| ZNF778 | Zinc finger protein 778 | 1.57 | 0.006798 | 0.091921 |
| ZNF785 | Zinc finger protein 785 | 1.51 | 0.008686 | 0.105086 |
| ZNF839 | Zinc finger protein 839 | 1.6 | 0.004878 | 0.078382 |
| ZNF850 | Zinc finger protein 850 | 2.39 | 0.000323 | 0.022134 |
| ZP3 | Zona pellucida glycoprotein 3 (sperm receptor) | 1.75 | 0.001213 | 0.038831 |
| ZRANB3 | Zinc finger, RAN-binding domain containing 3 | 1.63 | 0.001027 | 0.035964 |
| ***Down-regulated genes*** | | | | |
| ABCA1 | ATP-binding cassette, sub-family A (ABC1), member 1 | -1.89 | 0.000125 | 0.014763 |
| ABCA5 | ATP-binding cassette, sub-family A (ABC1), member 5 | -1.88 | 0.000883 | 0.033928 |
| ABCG2 | ATP-binding cassette, sub-family G (WHITE), member 2 | -1.99 | 0.000905 | 0.034182 |
| ABI3BP | ABI family, member 3 (NESH) binding protein | -1.88 | 0.000092 | 0.01372 |
| ACAA2 | Acetyl-coa acyltransferase 2 | -1.6 | 0.000035 | 0.010065 |
| ACAT2 | Acetyl-coa acetyltransferase 2 | -1.72 | 0.000266 | 0.020458 |
| ACSL5 | Acyl-coa synthetase long-chain family member 5 | -1.55 | 0.009261 | 0.108824 |
| ACYP1 | Acylphosphatase 1, erythrocyte (common) type | -1.62 | 0.000869 | 0.033537 |
| ADA | Adenosine deaminase | -1.78 | 0.000002 | 0.004166 |
| ADAM12 | ADAM metallopeptidase domain 12 | -1.96 | 0.000005 | 0.006491 |
| ADAMTS1 | ADAM metallopeptidase with thrombospondin type 1 motif, 1 | -3.32 | 0.000958 | 0.035051 |
| ADAMTS5 | ADAM metallopeptidase with thrombospondin type 1 motif, 5 | -2.16 | 0.005561 | 0.083408 |
| ADAMTS6 | ADAM metallopeptidase with thrombospondin type 1 motif, 6 | -1.7 | 0.002621 | 0.056024 |
| ADAMTSL1 | ADAMTS-like 1 | -1.9 | 0.000051 | 0.011748 |
| ADAT2 | Adenosine deaminase, trna-specific 2 | -1.52 | 0.01691 | 0.151423 |
| AGAP2 | Arfgap with gtpase domain, ankyrin repeat and PH domain 2 | -1.67 | 0.005895 | 0.085852 |
| AGTR1 | Angiotensin II receptor, type 1 | -1.53 | 0.00136 | 0.040801 |
| AHNAK2 | AHNAK nucleoprotein 2 | -2.18 | 0.000446 | 0.024904 |
| AHR | Aryl hydrocarbon receptor | -1.52 | 0.000051 | 0.011748 |
| AIM1 | Absent in melanoma 1 | -1.56 | 0.00195 | 0.049139 |
| AKAP1 | A kinase (PRKA) anchor protein 1 | -1.92 | 0.000178 | 0.017322 |
| ALPK2 | Alpha-kinase 2 | -1.78 | 0.002879 | 0.059023 |
| AMPD3 | Adenosine monophosphate deaminase 3 | -1.53 | 0.046098 | 0.261439 |
| ANKRD1 | Ankyrin repeat domain 1 (cardiac muscle) | -2.14 | 0.00232 | 0.052991 |
| ANKRD20A3, ANKRD20A4, ANKRD20A2, ANKRD20A1, ANKRD20A11P, ANKRD20A5P | Ankyrin repeat domain 20 family, member A3, ankyrin repeat domain 20 family, member A4, ankyrin repeat domain 20 family, member A2, ankyrin repeat domain 20 family, member A1, ankyrin repeat domain 20 family, member A11, pseudogene, ankyrin repeat domain 20 family, member A5, pseudogene | -1.6 | 0.011414 | 0.122805 |
| ANKRD20A5P | Ankyrin repeat domain 20 family, member A5, pseudogene | -1.65 | 0.000299 | 0.021228 |
| ANKRD30B | Ankyrin repeat domain 30B | -1.84 | 0.001658 | 0.04505 |
| ANKRD36B | Ankyrin repeat domain 36B | -1.57 | 0.000446 | 0.024904 |
| ANKRD44 | Ankyrin repeat domain 44 | -1.59 | 0.002038 | 0.050236 |
| ANXA10 | Annexin A10 | -1.75 | 0.000554 | 0.027481 |
| ANXA2 | Annexin A2 | -1.56 | 0.000455 | 0.025147 |
| ANXA2R | Annexin A2 receptor | -1.51 | 0.000201 | 0.018367 |
| ANXA8, ANXA8L1, ANXA8L2, LOC100652762 | Annexin A8, annexin A8-like 1, annexin A8-like 2, uncharacterized LOC100652762 | -1.6 | 0.000895 | 0.034123 |
| ANXA8L1, ANXA8, ANXA8L2 | Annexin A8-like 1, annexin A8, annexin A8-like 2 | -1.74 | 0.00214 | 0.05138 |
| ANXA8L2, ANXA8, ANXA8L1, FAM25B, FAM25C, FAM25G, FAM25A, FAM25HP, LOC100652762 | Annexin A8-like 2, annexin A8, annexin A8-like 1, family with sequence similarity 25, member B, family with sequence similarity 25, member C, family with sequence similarity 25, member G, family with sequence similarity 25, member A, family with sequence similarity 25, member H pseudogene, uncharacterized LOC100652762 | -1.54 | 0.002066 | 0.050518 |
| AOX1 | Aldehyde oxidase 1 | -1.83 | 0.000105 | 0.014264 |
| ARHGAP20 | Rho gtpase activating protein 20 | -1.67 | 0.004809 | 0.077738 |
| ARHGAP24 | Rho gtpase activating protein 24 | -1.54 | 0.000593 | 0.028347 |
| ARID5B | AT rich interactive domain 5B (MRF1-like) | -1.59 | 0.000042 | 0.010996 |
| ARMC4 | Armadillo repeat containing 4 | -1.8 | 0.00154 | 0.04315 |
| ATF3 | Activating transcription factor 3 | -1.63 | 0.009201 | 0.108373 |
| ATOH8 | Atonal homolog 8 (Drosophila) | -1.87 | 0.006094 | 0.087175 |
| ATP2B4 | Atpase, Ca++ transporting, plasma membrane 4 | -1.95 | 0.000025 | 0.009862 |
| B3GNT4 | UDP-glcnac:betagal beta-1,3-N-acetylglucosaminyltransferase 4 | -1.68 | 0.000548 | 0.02742 |
| B4GALNT1 | Beta-1,4-N-acetyl-galactosaminyl transferase 1 | -1.6 | 0.000815 | 0.03235 |
| BACH2 | BTB and CNC homology 1, basic leucine zipper transcription factor 2 | -1.6 | 0.006858 | 0.092311 |
| BCAR3 | Breast cancer anti-estrogen resistance 3 | -1.53 | 0.00197 | 0.049384 |
| BCL3 | B-cell CLL/lymphoma 3 | -1.79 | 0.000447 | 0.024904 |
| BCL7C | B-cell CLL/lymphoma 7C | -1.53 | 0.000062 | 0.012629 |
| BDKRB1 | Bradykinin receptor B1 | -1.67 | 0.000236 | 0.019468 |
| BEX1 | Brain expressed, X-linked 1 | -2.37 | 0.000018 | 0.009534 |
| BHLHE40 | Basic helix-loop-helix family, member e40 | -1.67 | 0.005229 | 0.080723 |
| BICC1 | Bicaudal C homolog 1 (Drosophila) | -1.57 | 0.000161 | 0.016637 |
| BLID | BH3-like motif containing, cell death inducer | -2.2 | 0.000135 | 0.015156 |
| BLM | Bloom syndrome, recq helicase-like | -1.66 | 0.000197 | 0.018215 |
| BMPER | BMP binding endothelial regulator | -2.21 | 0.000131 | 0.015116 |
| BNC2 | Basonuclin 2 | -1.54 | 0.002274 | 0.052699 |
| BRCA1, LOC100505899 | Breast cancer 1, early onset, uncharacterized LOC100505899 | -1.53 | 0.002298 | 0.05293 |
| C10orf122 | Chromosome 10 open reading frame 122 | -1.65 | 0.003508 | 0.065558 |
| C10orf136 | Chromosome 10 open reading frame 136 | -2.53 | 0.001151 | 0.037903 |
| C10orf55, PLAU | Chromosome 10 open reading frame 55, plasminogen activator, urokinase | -1.52 | 0.005405 | 0.082143 |
| C10orf67 | Chromosome 10 open reading frame 67 | -1.55 | 0.001909 | 0.048754 |
| C10orf68, CCDC7 | Chromosome 10 open reading frame 68, coiled-coil domain containing 7 | -1.59 | 0.008964 | 0.106705 |
| C13orf33 | Chromosome 13 open reading frame 33 | -1.92 | 0.000984 | 0.035437 |
| C19orf73 | Chromosome 19 open reading frame 73 | -1.61 | 0.002026 | 0.050129 |
| C1orf233 | Chromosome 1 open reading frame 233 | -1.88 | 0.012198 | 0.127065 |
| C1orf51 | Chromosome 1 open reading frame 51 | -1.64 | 0.0004 | 0.023831 |
| C1QL4 | Complement component 1, q subcomponent-like 4 | -1.94 | 0.002097 | 0.050937 |
| C1QTNF2 | C1q and tumor necrosis factor related protein 2 | -2.51 | 0.001843 | 0.047938 |
| C1QTNF9B-AS1 | C1QTNF9B antisense RNA 1 (non-protein coding) | -1.88 | 0.01152 | 0.123367 |
| C2orf27A | Chromosome 2 open reading frame 27A | -1.61 | 0.004945 | 0.078632 |
| C3orf67 | Chromosome 3 open reading frame 67 | -1.66 | 0.002271 | 0.052683 |
| C5orf4 | Chromosome 5 open reading frame 4 | -1.76 | 0.000441 | 0.02487 |
| C6orf132 | Chromosome 6 open reading frame 132 | -1.81 | 0.0004 | 0.023831 |
| C7orf29 | Chromosome 7 open reading frame 29 | -1.54 | 0.0061 | 0.087201 |
| C7orf41 | Chromosome 7 open reading frame 41 | -1.75 | 0.001243 | 0.039352 |
| C7orf69 | Chromosome 7 open reading frame 69 | -1.91 | 0.003681 | 0.067446 |
| C8orf37 | Chromosome 8 open reading frame 37 | -1.55 | 0.020627 | 0.168365 |
| CA9 | Carbonic anhydrase IX | -1.74 | 0.006189 | 0.08769 |
| CAHM | Colon adenocarcinoma hypermethylated (non-protein coding) | -1.64 | 0.007567 | 0.097665 |
| CAMK1D, LOC283070 | Calcium/calmodulin-dependent protein kinase ID, uncharacterized LOC283070 | -1.84 | 0.012891 | 0.130366 |
| CARD6 | Caspase recruitment domain family, member 6 | -2.17 | 0.00011 | 0.014264 |
| CBLB | Cbl proto-oncogene, E3 ubiquitin protein ligase B | -1.56 | 0.000089 | 0.013713 |
| CC2D2A | Coiled-coil and C2 domain containing 2A | -1.56 | 0.00168 | 0.045424 |
| CCBE1 | Collagen and calcium binding EGF domains 1 | -2.27 | 0.000002 | 0.003839 |
| CCDC109B | Coiled-coil domain containing 109B | -1.7 | 0.000182 | 0.017355 |
| CCDC112 | Coiled-coil domain containing 112 | -1.58 | 0.021579 | 0.172453 |
| CCDC80 | Coiled-coil domain containing 80 | -2.41 | 0.000005 | 0.005961 |
| CCL2 | Chemokine (C-C motif) ligand 2 | -1.57 | 0.004148 | 0.07184 |
| CCL28 | Chemokine (C-C motif) ligand 28 | -2.19 | 0.001693 | 0.045641 |
| CCNE1 | Cyclin E1 | -2.11 | 0.000071 | 0.013178 |
| CCNE2 | Cyclin E2 | -2.93 | 0.000067 | 0.012906 |
| CD109 | CD109 molecule | -1.96 | 0.000024 | 0.009862 |
| CD177 | CD177 molecule | -1.52 | 0.028954 | 0.202933 |
| CD24 | CD24 molecule | -2.18 | 0.000061 | 0.012588 |
| CD44 | CD44 molecule (Indian blood group) | -1.6 | 0.000668 | 0.029519 |
| CDA | Cytidine deaminase | -1.57 | 0.001082 | 0.036927 |
| CDC45 | Cell division cycle 45 homolog (S. Cerevisiae) | -1.87 | 0.00027 | 0.020552 |
| CDC6 | Cell division cycle 6 homolog (S. Cerevisiae) | -1.69 | 0.000276 | 0.020677 |
| CDCA7 | Cell division cycle associated 7 | -1.88 | 0.000084 | 0.013636 |
| CDCA7L | Cell division cycle associated 7-like | -1.56 | 0.000036 | 0.010179 |
| CDKL2 | Cyclin-dependent kinase-like 2 (CDC2-related kinase) | -1.58 | 0.009744 | 0.111664 |
| CDKN2B | Cyclin-dependent kinase inhibitor 2B (p15, inhibits CDK4) | -1.52 | 0.002195 | 0.051878 |
| CDON | Cdon homolog (mouse) | -1.85 | 0.002986 | 0.060065 |
| CEACAMP6, CEACAMP7, CEACAMP9, LOC284344, CEACAMP11 | Carcinoembryonic antigen-related cell adhesion molecule pseudogene 6, carcinoembryonic antigen-related cell adhesion molecule pseudogene 7, carcinoembryonic antigen-related cell adhesion molecule pseudogene 9, uncharacterized LOC284344, carcinoembryonic antigen-related cell adhesion molecule pseudogene 11 | -2.54 | 0.001701 | 0.045707 |
| CELF2 | CUGBP, Elav-like family member 2 | -1.54 | 0.002225 | 0.052102 |
| CEP250 | Centrosomal protein 250kda | -1.53 | 0.000272 | 0.020558 |
| CFC1B | Cripto, FRL-1, cryptic family 1B | -1.67 | 0.036502 | 0.230334 |
| CGB8, CGB1, CGB5, CGB | Chorionic gonadotropin, beta polypeptide 8, chorionic gonadotropin, beta polypeptide 1, chorionic gonadotropin, beta polypeptide 5, chorionic gonadotropin, beta polypeptide | -1.68 | 0.019766 | 0.164745 |
| CHML | Choroideremia-like (Rab escort protein 2) | -1.92 | 0.000023 | 0.009862 |
| CHRDL1 | Chordin-like 1 | -1.73 | 0.002423 | 0.054143 |
| CLDN1 | Claudin 1 | -1.56 | 0.000341 | 0.022619 |
| CLMP | CXADR-like membrane protein | -2.03 | 0.000014 | 0.009534 |
| CLSPN | Claspin | -1.84 | 0.001964 | 0.049362 |
| CNIH2 | Cornichon homolog 2 (Drosophila) | -1.66 | 0.002761 | 0.057594 |
| CNPY2 | Canopy 2 homolog (zebrafish) | -1.95 | 0.001975 | 0.049415 |
| CNTNAP1 | Contactin associated protein 1 | -1.77 | 0.000659 | 0.029396 |
| CPA3 | Carboxypeptidase A3 (mast cell) | -1.6 | 0.029479 | 0.2049 |
| CPA4 | Carboxypeptidase A4 | -3.23 | 0.00001 | 0.008694 |
| CREB5, LOC401317 | Camp responsive element binding protein 5, uncharacterized LOC401317 | -2.14 | 0.000015 | 0.009534 |
| CREBRF | CREB3 regulatory factor | -1.54 | 0.000015 | 0.009534 |
| CRISPLD2 | Cysteine-rich secretory protein LCCL domain containing 2 | -1.87 | 0.000282 | 0.020811 |
| CSNK1G1 | Casein kinase 1, gamma 1 | -1.67 | 0.000159 | 0.016603 |
| CSRP2BP | CSRP2 binding protein | -1.55 | 0.008511 | 0.103983 |
| CTAGE11P | CTAGE family, member 11, pseudogene | -2.78 | 0.006211 | 0.08785 |
| CXADR, BTG3 | Coxsackie virus and adenovirus receptor, BTG family, member 3 | -2.85 | 0.003241 | 0.062877 |
| CXCR7 | Chemokine (C-X-C motif) receptor 7 | -1.68 | 0.000872 | 0.033599 |
| CYP1B1 | Cytochrome P450, family 1, subfamily B, polypeptide 1 | -2.94 | 0.000003 | 0.004785 |
| CYP24A1 | Cytochrome P450, family 24, subfamily A, polypeptide 1 | -1.61 | 0.00049 | 0.025827 |
| CYP26B1 | Cytochrome P450, family 26, subfamily B, polypeptide 1 | -1.65 | 0.003497 | 0.065446 |
| CYP51A1, LRRD1 | Cytochrome P450, family 51, subfamily A, polypeptide 1, leucine-rich repeats and death domain containing 1 | -1.72 | 0.000363 | 0.023103 |
| DACT1 | Dapper, antagonist of beta-catenin, homolog 1 (Xenopus laevis) | -1.57 | 0.00009 | 0.013713 |
| DBP | D site of albumin promoter (albumin D-box) binding protein | -1.65 | 0.000742 | 0.030888 |
| DCBLD1 | Discoidin, CUB and LCCL domain containing 1 | -2.06 | 0.000024 | 0.009862 |
| DCLRE1A | DNA cross-link repair 1A | -1.56 | 0.001034 | 0.036151 |
| DCTPP1 | Dctp pyrophosphatase 1 | -1.52 | 0.000387 | 0.023729 |
| DDB2 | Damage-specific DNA binding protein 2, 48kda | -1.73 | 0.001112 | 0.037267 |
| DDX26B | DEAD/H (Asp-Glu-Ala-Asp/His) box polypeptide 26B | -1.67 | 0.003725 | 0.067882 |
| DDX60L | DEAD (Asp-Glu-Ala-Asp) box polypeptide 60-like | -1.67 | 0.000728 | 0.030677 |
| DGKI | Diacylglycerol kinase, iota | -2.86 | 0.000612 | 0.02868 |
| DHCR7 | 7-dehydrocholesterol reductase | -1.59 | 0.000125 | 0.014763 |
| DHRS4L2, DHRS4L1 | Dehydrogenase/reductase (SDR family) member 4 like 2, dehydrogenase/reductase (SDR family) member 4 like 1 | -1.63 | 0.000339 | 0.022619 |
| DIO2 | Deiodinase, iodothyronine, type II | -5.45 | 0.000012 | 0.009534 |
| DIP2A-IT1 | DIP2A intronic transcript 1 (non-protein coding) | -1.57 | 0.008971 | 0.106718 |
| DKK1 | Dickkopf 1 homolog (Xenopus laevis) | -2.9 | 0.000469 | 0.02547 |
| DMRTA1 | DMRT-like family A1 | -1.91 | 0.008721 | 0.105198 |
| DOCK11 | Dedicator of cytokinesis 11 | -1.86 | 0.001675 | 0.045358 |
| DPYD-IT1 | DPYD intronic transcript 1 (non-protein coding) | -1.62 | 0.023338 | 0.179948 |
| DRAM1 | DNA-damage regulated autophagy modulator 1 | -1.54 | 0.000792 | 0.031942 |
| DSN1 | DSN1, MIND kinetochore complex component, homolog (S. Cerevisiae) | -1.65 | 0.000082 | 0.013636 |
| DTL | Denticleless E3 ubiquitin protein ligase homolog (Drosophila) | -1.9 | 1.31E-08 | 0.000351 |
| DTNA | Dystrobrevin, alpha | -1.69 | 0.003254 | 0.062996 |
| DUSP22, LOC100653247 | Dual specificity phosphatase 22, dual specificity protein phosphatase 22-like | -1.56 | 0.000136 | 0.015179 |
| DUSP4 | Dual specificity phosphatase 4 | -1.55 | 0.007275 | 0.095251 |
| DUT | Deoxyuridine triphosphatase | -1.65 | 0.007766 | 0.098986 |
| DUXAP10, FLJ39632, LOC100506303, LOC400879, LOC100653149 | Double homeobox A pseudogene 10, uncharacterized LOC642477, uncharacterized LOC100506303, uncharacterized LOC400879, uncharacterized LOC100653149 | -1.82 | 0.003372 | 0.064018 |
| E2F1 | E2F transcription factor 1 | -1.61 | 0.000413 | 0.02411 |
| E2F2 | E2F transcription factor 2 | -2.19 | 0.000423 | 0.024333 |
| E2F7 | E2F transcription factor 7 | -1.84 | 0.000081 | 0.013636 |
| EBP | Emopamil binding protein (sterol isomerase) | -1.57 | 0.00083 | 0.032671 |
| EDN1 | Endothelin 1 | -1.53 | 0.005008 | 0.079183 |
| EGR1 | Early growth response 1 | -2.09 | 0.001806 | 0.047346 |
| EIF4E3 | Eukaryotic translation initiation factor 4E family member 3 | -1.71 | 0.007494 | 0.096885 |
| ELF4 | E74-like factor 4 (ets domain transcription factor) | -1.54 | 0.000832 | 0.032688 |
| ELOVL2 | ELOVL fatty acid elongase 2 | -1.85 | 0.000336 | 0.022564 |
| ELOVL6 | ELOVL fatty acid elongase 6 | -1.53 | 0.00026 | 0.020233 |
| EMP2 | Epithelial membrane protein 2 | -1.54 | 0.001711 | 0.045917 |
| ENO1-IT1 | ENO1 intronic transcript 1 (non-protein coding) | -1.51 | 0.003148 | 0.061805 |
| ENOSF1 | Enolase superfamily member 1 | -1.56 | 0.000104 | 0.014213 |
| ENPP2 | Ectonucleotide pyrophosphatase/phosphodiesterase 2 | -1.8 | 0.005436 | 0.082381 |
| ENTPD4 | Ectonucleoside triphosphate diphosphohydrolase 4 | -2.03 | 0.001333 | 0.040512 |
| EPGN | Epithelial mitogen homolog (mouse) | -3.06 | 0.005465 | 0.082634 |
| EPHA4 | EPH receptor A4 | -1.68 | 0.001442 | 0.042024 |
| EPHB2 | EPH receptor B2 | -1.53 | 0.000695 | 0.029882 |
| ERVK-7 | Endogenous retrovirus group K, member 7 | -2.45 | 0.008495 | 0.103852 |
| EXO1 | Exonuclease 1 | -2.25 | 0.000014 | 0.009534 |
| EXOSC5 | Exosome component 5 | -1.98 | 0.000395 | 0.023776 |
| F2RL1 | Coagulation factor II (thrombin) receptor-like 1 | -1.63 | 0.002716 | 0.057104 |
| F2RL2 | Coagulation factor II (thrombin) receptor-like 2 | -3.92 | 0.000069 | 0.013076 |
| F3 | Coagulation factor III (thromboplastin, tissue factor) | -2.8 | 0.000019 | 0.009534 |
| FAM101B | Family with sequence similarity 101, member B | -1.66 | 0.000124 | 0.014763 |
| FAM111B | Family with sequence similarity 111, member B | -2.59 | 0.000012 | 0.009534 |
| FAM133CP | Family with sequence similarity 133, member C, pseudogene | -1.65 | 0.012497 | 0.128457 |
| FAM13A-AS1 | FAM13A antisense RNA 1 (non-protein coding) | -1.85 | 0.000509 | 0.026203 |
| FAM198B | Family with sequence similarity 198, member B | -1.61 | 0.011562 | 0.123591 |
| FAM20C | Family with sequence similarity 20, member C | -1.59 | 0.000102 | 0.014213 |
| FAM219A | Family with sequence similarity 219, member A | -1.62 | 0.018789 | 0.160727 |
| FAM57A | Family with sequence similarity 57, member A | -1.54 | 0.000668 | 0.029519 |
| FAM65B | Family with sequence similarity 65, member B | -1.56 | 0.012207 | 0.127097 |
| FAM70A | Family with sequence similarity 70, member A | -1.77 | 0.000091 | 0.013715 |
| FASN | Fatty acid synthase | -1.53 | 0.000723 | 0.030586 |
| FAT4 | FAT tumor suppressor homolog 4 (Drosophila) | -2.19 | 0.000308 | 0.021559 |
| FAXC | Failed axon connections homolog (Drosophila) | -1.78 | 0.005185 | 0.080455 |
| FBXO32 | F-box protein 32 | -3.06 | 0.000458 | 0.025272 |
| FEN1 | Flap structure-specific endonuclease 1 | -1.67 | 0.004393 | 0.074024 |
| FER | Fer (fps/fes related) tyrosine kinase | -1.58 | 0.000169 | 0.016947 |
| FGD4 | FYVE, rhogef and PH domain containing 4 | -1.84 | 0.001295 | 0.040037 |
| FILIP1L | Filamin A interacting protein 1-like | -1.67 | 0.000434 | 0.024665 |
| FJX1 | Four jointed box 1 (Drosophila) | -1.51 | 0.003431 | 0.064703 |
| FLG-AS1 | FLG antisense RNA 1 (non-protein coding) | -1.53 | 0.00022 | 0.019095 |
| FLJ22447 | Uncharacterized LOC400221 | -2.98 | 0.000122 | 0.014737 |
| FLJ27352 | Uncharacterized LOC145788 | -1.53 | 0.0042 | 0.072319 |
| FLJ35282 | Uncharacterized LOC441389 | -1.62 | 0.010751 | 0.118565 |
| FLJ35946 | Uncharacterized protein FLJ35946 | -2.35 | 0.000292 | 0.021099 |
| FLJ38717 | FLJ38717 protein | -1.56 | 0.002807 | 0.0581 |
| FLJ44342 | Uncharacterized LOC645460 | -1.65 | 0.004293 | 0.072996 |
| FLRT2, LOC100506718 | Fibronectin leucine rich transmembrane protein 2, uncharacterized LOC100506718 | -2.05 | 0.000237 | 0.019468 |
| FNIP2 | Folliculin interacting protein 2 | -1.54 | 0.001669 | 0.045276 |
| FOLR3 | Folate receptor 3 (gamma) | -1.52 | 0.002367 | 0.053465 |
| FRMD4A | FERM domain containing 4A | -1.52 | 0.000941 | 0.034714 |
| FRMD6 | FERM domain containing 6 | -2.05 | 0.000154 | 0.016254 |
| FSTL3 | Follistatin-like 3 (secreted glycoprotein) | -1.87 | 0.001195 | 0.038508 |
| FTX | FTX transcript, XIST regulator (non-protein coding) | -1.57 | 0.000648 | 0.029387 |
| FUT11 | Fucosyltransferase 11 (alpha (1,3) fucosyltransferase) | -1.62 | 0.000164 | 0.016817 |
| FZD4 | Frizzled family receptor 4 | -1.55 | 0.017314 | 0.153386 |
| GAB1 | GRB2-associated binding protein 1 | -1.62 | 0.004048 | 0.070918 |
| GABARAPL1 | GABA(A) receptor-associated protein like 1 | -1.76 | 0.00028 | 0.020788 |
| GABRE, MIR452, MIR224 | Gamma-aminobutyric acid (GABA) A receptor, epsilon, microrna 452, microrna 224 | -1.68 | 0.000141 | 0.01546 |
| GABRQ | Gamma-aminobutyric acid (GABA) A receptor, theta | -1.58 | 0.000544 | 0.027386 |
| GALNT12 | UDP-N-acetyl-alpha-D-galactosamine:polypeptide N-acetylgalactosaminyltransferase 12 (galnac-T12) | -1.51 | 0.028331 | 0.200616 |
| GALNT5 | UDP-N-acetyl-alpha-D-galactosamine:polypeptide N-acetylgalactosaminyltransferase 5 (galnac-T5) | -1.9 | 0.005586 | 0.083532 |
| GAS1 | Growth arrest-specific 1 | -2.05 | 0.00009 | 0.013713 |
| GBP3 | Guanylate binding protein 3 | -1.83 | 0.008599 | 0.104477 |
| GDNF | Glial cell derived neurotrophic factor | -1.65 | 0.00338 | 0.064121 |
| GINS2 | GINS complex subunit 2 (Psf2 homolog) | -2.15 | 0.000053 | 0.011896 |
| GINS3 | GINS complex subunit 3 (Psf3 homolog) | -1.65 | 0.00121 | 0.038778 |
| GLYATL1, LOC100287413 | Glycine-N-acyltransferase-like 1, uncharacterized LOC100287413 | -1.6 | 0.003906 | 0.069563 |
| GLYATL2 | Glycine-N-acyltransferase-like 2 | -1.64 | 0.015529 | 0.144102 |
| GMEB1 | Glucocorticoid modulatory element binding protein 1 | -1.71 | 0.000193 | 0.017845 |
| GNG2 | Guanine nucleotide binding protein (G protein), gamma 2 | -1.52 | 0.007428 | 0.096483 |
| GPR137C | G protein-coupled receptor 137C | -2.21 | 0.000079 | 0.013636 |
| GPR146, LOC100505568, LOC100505551 | G protein-coupled receptor 146, uncharacterized LOC100505568, uncharacterized LOC100505551 | -1.51 | 0.000755 | 0.031041 |
| GPR52 | G protein-coupled receptor 52 | -1.64 | 0.000042 | 0.010979 |
| GPR56 | G protein-coupled receptor 56 | -2.39 | 0.000172 | 0.017115 |
| GPR87 | G protein-coupled receptor 87 | -2.18 | 0.000083 | 0.013636 |
| GPRC5A | G protein-coupled receptor, family C, group 5, member A | -2.03 | 0.000371 | 0.023416 |
| GPRC5A, MIR614 | G protein-coupled receptor, family C, group 5, member A, microrna 614 | -2.54 | 0.000293 | 0.021107 |
| GSG1 | Germ cell associated 1 | -1.52 | 0.004488 | 0.074793 |
| GSTA2, GSTA1 | Glutathione S-transferase alpha 2, glutathione S-transferase alpha 1 | -2.65 | 0.005475 | 0.082698 |
| H1F0 | H1 histone family, member 0 | -1.9 | 0.000975 | 0.035345 |
| H1FX | H1 histone family, member X | -1.6 | 0.005107 | 0.079839 |
| HBEGF | Heparin-binding EGF-like growth factor | -1.99 | 0.004085 | 0.071234 |
| HCP5 | HLA complex P5 (non-protein coding) | -2.53 | 0.000239 | 0.01947 |
| HECW2 | HECT, C2 and WW domain containing E3 ubiquitin protein ligase 2 | -1.74 | 0.001151 | 0.037903 |
| HEG1 | HEG homolog 1 (zebrafish) | -2.06 | 0.000019 | 0.009534 |
| HELB | Helicase (DNA) B | -1.78 | 0.007181 | 0.094694 |
| HELLS | Helicase, lymphoid-specific | -1.97 | 0.000048 | 0.011574 |
| HERC3 | HECT and RLD domain containing E3 ubiquitin protein ligase 3 | -1.94 | 0.000071 | 0.013196 |
| HERC4 | HECT and RLD domain containing E3 ubiquitin protein ligase 4 | -1.55 | 0.000035 | 0.010065 |
| HIST1H1B | Histone cluster 1, h1b | -1.78 | 0.000642 | 0.029385 |
| HIST1H1C | Histone cluster 1, h1c | -1.77 | 0.000241 | 0.019499 |
| HIST1H2AB, HIST1H2AE | Histone cluster 1, h2ab, histone cluster 1, h2ae | -1.79 | 0.00798 | 0.100362 |
| HIST1H2AE, HIST1H2AB | Histone cluster 1, h2ae, histone cluster 1, h2ab | -1.53 | 0.010454 | 0.116459 |
| HIST1H2BB | Histone cluster 1, h2bb | -2.03 | 0.000221 | 0.019151 |
| HIST1H2BC, HIST1H2BI, HIST1H2BE, HIST1H2BF, HIST1H2BG | Histone cluster 1, h2bc, histone cluster 1, h2bi, histone cluster 1, h2be, histone cluster 1, h2bf, histone cluster 1, h2bg | -1.65 | 0.003905 | 0.069563 |
| HIST1H3J, HIST1H3F, HIST1H3B, HIST1H3H, HIST1H3G, HIST1H3I, HIST1H3E, HIST1H3C, HIST1H3D, HIST1H3A | Histone cluster 1, h3j, histone cluster 1, h3f, histone cluster 1, h3b, histone cluster 1, h3h, histone cluster 1, h3g, histone cluster 1, h3i, histone cluster 1, h3e, histone cluster 1, h3c, histone cluster 1, h3d, histone cluster 1, h3a | -1.66 | 0.011873 | 0.125137 |
| HIST2H2AC | Histone cluster 2, h2ac | -1.58 | 0.001772 | 0.046764 |
| HIST2H4B, HIST4H4, HIST2H4A, HIST1H4L, HIST1H4E, HIST1H4B, HIST1H4H, HIST1H4C, HIST1H4J, HIST1H4K, HIST1H4F, HIST1H4D, HIST1H4A, HIST1H4I | Histone cluster 2, h4b, histone cluster 4, H4, histone cluster 2, h4a, histone cluster 1, h4l, histone cluster 1, h4e, histone cluster 1, h4b, histone cluster 1, h4h, histone cluster 1, h4c, histone cluster 1, h4j, histone cluster 1, h4k, histone cluster 1, h4f, histone cluster 1, h4d, histone cluster 1, h4a, histone cluster 1, h4i | -1.58 | 0.003723 | 0.06788 |
| HIVEP1 | Human immunodeficiency virus type I enhancer binding protein 1 | -1.54 | 0.000777 | 0.031584 |
| HLF | Hepatic leukemia factor | -6.4 | 0.000018 | 0.009534 |
| HMGCS1 | 3-hydroxy-3-methylglutaryl-coa synthase 1 (soluble) | -2.04 | 0.000059 | 0.012266 |
| HMGN3 | High mobility group nucleosomal binding domain 3 | -1.69 | 0.009768 | 0.111813 |
| IFI16 | Interferon, gamma-inducible protein 16 | -2.01 | 0.000023 | 0.009862 |
| IFITM1 | Interferon induced transmembrane protein 1 | -1.94 | 0.000097 | 0.014059 |
| IGANRP | Uncharacterized LOC100652933 | -1.75 | 0.014172 | 0.137036 |
| IGFBP3 | Insulin-like growth factor binding protein 3 | -2.62 | 0.000568 | 0.027718 |
| IGSF23 | Immunoglobulin superfamily, member 23 | -1.66 | 0.031716 | 0.213177 |
| IGSF3 | Immunoglobulin superfamily, member 3 | -1.62 | 0.013702 | 0.134702 |
| IKBKE | Inhibitor of kappa light polypeptide gene enhancer in B-cells, kinase epsilon | -1.85 | 0.003966 | 0.070209 |
| IL1R1 | Interleukin 1 receptor, type I | -1.53 | 0.004033 | 0.070829 |
| IL27RA | Interleukin 27 receptor, alpha | -1.68 | 0.021513 | 0.172032 |
| IL31RA | Interleukin 31 receptor A | -1.52 | 0.005304 | 0.08133 |
| IL6R | Interleukin 6 receptor | -1.55 | 0.001537 | 0.04315 |
| IL7R | Interleukin 7 receptor | -2.52 | 0.000014 | 0.009534 |
| IL8 | Interleukin 8 | -1.7 | 0.021747 | 0.173076 |
| INSIG1 | Insulin induced gene 1 | -2.27 | 0.000021 | 0.009644 |
| INSR | Insulin receptor | -1.53 | 0.002284 | 0.052855 |
| IPW | Imprinted in Prader-Willi syndrome (non-protein coding) | -1.85 | 0.000257 | 0.020004 |
| IPW, SNRPN, LOC100506948, SNORD116-28, SNORD115-26, SNORD115-13, SNORD115-7, SNORD107 | Imprinted in Prader-Willi syndrome (non-protein coding), small nuclear ribonucleoprotein polypeptide N, uncharacterized LOC100506948, small nucleolar RNA, C/D box 116-28, small nucleolar RNA, C/D box 115-26, small nucleolar RNA, C/D box 115-13, small nucleolar RNA, C/D box 115-7, small nucleolar RNA, C/D box 107 | -1.83 | 0.001799 | 0.047226 |
| IRAK4 | Interleukin-1 receptor-associated kinase 4 | -1.69 | 0.000524 | 0.026722 |
| ITGB4 | Integrin, beta 4 | -1.64 | 0.000673 | 0.029549 |
| ITGBL1 | Integrin, beta-like 1 (with EGF-like repeat domains) | -1.6 | 0.00013 | 0.015019 |
| ITPKB | Inositol-trisphosphate 3-kinase B | -1.61 | 0.015721 | 0.144951 |
| ITPR3 | Inositol 1,4,5-trisphosphate receptor, type 3 | -1.57 | 0.001074 | 0.036852 |
| JUN | Jun proto-oncogene | -2.67 | 0.000154 | 0.016254 |
| KC6 | Keratoconus gene 6 | -1.65 | 0.033871 | 0.22069 |
| KCNH1 | Potassium voltage-gated channel, subfamily H (eag-related), member 1 | -1.71 | 0.000936 | 0.034631 |
| KCNH1-IT1 | KCNH1 intronic transcript 1 (non-protein coding) | -1.68 | 0.043721 | 0.25469 |
| KCTD4 | Potassium channel tetramerisation domain containing 4 | -1.51 | 0.024197 | 0.184333 |
| KIAA1009 | Kiaa1009 | -1.58 | 0.009493 | 0.110339 |
| KIAA1199 | Kiaa1199 | -2.49 | 0.000051 | 0.011748 |
| KIAA1644 | Kiaa1644 | -1.85 | 0.003595 | 0.066519 |
| KIRREL3-AS2 | KIRREL3 antisense RNA 2 (non-protein coding) | -1.67 | 0.033986 | 0.221141 |
| KIT | V-kit Hardy-Zuckerman 4 feline sarcoma viral oncogene homolog | -1.59 | 0.000978 | 0.035378 |
| KLF10 | Kruppel-like factor 10 | -2.1 | 0.000135 | 0.015156 |
| KLF11 | Kruppel-like factor 11 | -1.69 | 0.00053 | 0.026895 |
| KLF6 | Kruppel-like factor 6 | -1.74 | 0.000194 | 0.017926 |
| KLF7-IT1 | KLF7 intronic transcript 1 (non-protein coding) | -1.76 | 0.000289 | 0.021036 |
| KLF9 | Kruppel-like factor 9 | -1.85 | 0.001475 | 0.042395 |
| KLHL24 | Kelch-like 24 (Drosophila) | -1.93 | 0.000238 | 0.019468 |
| KLHL4 | Kelch-like 4 (Drosophila) | -1.61 | 0.003876 | 0.069318 |
| KRT17P1 | Keratin 17 pseudogene 1 | -1.84 | 0.003292 | 0.063335 |
| KRT23 | Keratin 23 (histone deacetylase inducible) | -1.63 | 0.010832 | 0.118983 |
| KRT7 | Keratin 7 | -1.6 | 0.018411 | 0.158964 |
| KRT80 | Keratin 80 | -1.76 | 0.001771 | 0.046758 |
| L1CAM | L1 cell adhesion molecule | -1.52 | 0.000465 | 0.025441 |
| LACC1 | Laccase (multicopper oxidoreductase) domain containing 1 | -1.55 | 0.042972 | 0.251915 |
| LAMC2 | Laminin, gamma 2 | -1.7 | 0.002305 | 0.052968 |
| LIF | Leukemia inhibitory factor | -1.51 | 0.006343 | 0.088774 |
| LIG1 | Ligase I, DNA, ATP-dependent | -1.53 | 0.002916 | 0.059349 |
| LINC00173 | Long intergenic non-protein coding RNA 173 | -1.55 | 0.043845 | 0.255026 |
| LINC00342 | Long intergenic non-protein coding RNA 342 | -1.85 | 0.019886 | 0.165306 |
| LINC00478 | Long intergenic non-protein coding RNA 478 | -2.22 | 0.001494 | 0.042692 |
| LINC00511 | Long intergenic non-protein coding RNA 511 | -2.26 | 0.012183 | 0.127012 |
| LOC100128288 | Uncharacterized LOC100128288 | -1.75 | 0.012552 | 0.128758 |
| LOC100128822 | Uncharacterized LOC100128822 | -1.58 | 0.00017 | 0.016997 |
| LOC100129502 | Uncharacterized LOC100129502 | -1.99 | 0.000471 | 0.025494 |
| LOC100130976 | Uncharacterized LOC100130976 | -2.04 | 0.009304 | 0.109109 |
| LOC100131234 | Familial acute myelogenous leukemia related factor | -1.73 | 0.000728 | 0.030677 |
| LOC100133106 | Vcew9374 | -1.91 | 0.01499 | 0.141032 |
| LOC100289092 | Uncharacterized LOC100289092 | -1.63 | 0.005806 | 0.085168 |
| LOC100289627, SNORD96A, SNORD95, GNB2L1 | Uncharacterized LOC100289627, small nucleolar RNA, C/D box 96A, small nucleolar RNA, C/D box 95, guanine nucleotide binding protein (G protein), beta polypeptide 2-like 1 | -1.54 | 0.014375 | 0.138047 |
| LOC100289650 | Uncharacterized LOC100289650 | -4.22 | 0.001275 | 0.039887 |
| LOC100499467 | Uncharacterized LOC100499467 | -1.59 | 0.003665 | 0.067343 |
| LOC100505516 | Uncharacterized LOC100505516 | -1.81 | 0.000995 | 0.035582 |
| LOC100505666 | Uncharacterized LOC100505666 | -1.62 | 0.005396 | 0.082115 |
| LOC100505701 | Uncharacterized LOC100505701 | -6.21 | 0.000237 | 0.019468 |
| LOC100505944 | Uncharacterized LOC100505944 | -2.12 | 0.000038 | 0.010605 |
| LOC100506303, LOC400879 | Uncharacterized LOC100506303, uncharacterized LOC400879 | -2.16 | 0.000071 | 0.013178 |
| LOC100506303, LOC400879, LOC100653149 | Uncharacterized LOC100506303, uncharacterized LOC400879, uncharacterized LOC100653149 | -2.19 | 0.000085 | 0.013677 |
| LOC100506835 | Uncharacterized LOC100506835 | -2.16 | 0.0096 | 0.111098 |
| LOC100506870 | Uncharacterized LOC100506870 | -3.55 | 0.000037 | 0.010273 |
| LOC100506895 | Uncharacterized LOC100506895 | -1.86 | 0.000366 | 0.023204 |
| LOC100506939 | Uncharacterized LOC100506939 | -1.96 | 0.029352 | 0.204494 |
| LOC100506948, SNORD116-28, SNORD115-26, SNORD115-13, SNORD115-7, SNORD107 | Uncharacterized LOC100506948, small nucleolar RNA, C/D box 116-28, small nucleolar RNA, C/D box 115-26, small nucleolar RNA, C/D box 115-13, small nucleolar RNA, C/D box 115-7, small nucleolar RNA, C/D box 107 | -3.32 | 0.004586 | 0.075728 |
| LOC100506965 | Uncharacterized LOC100506965 | -1.51 | 0.004545 | 0.075351 |
| LOC100507136 | Uncharacterized LOC100507136 | -1.91 | 0.000467 | 0.025441 |
| LOC100507460 | Uncharacterized LOC100507460 | -2.3 | 0.000216 | 0.01888 |
| LOC100507487 | Uncharacterized LOC100507487 | -1.6 | 0.001319 | 0.040302 |
| LOC100507516 | Uncharacterized LOC100507516 | -2.38 | 0.0011 | 0.037139 |
| LOC100509445, OVOS, OVOS2 | Uncharacterized LOC100509445, ovostatin, ovostatin 2 | -1.67 | 0.015838 | 0.145612 |
| LOC100653008, LOC100652914 | Uncharacterized LOC100653008, uncharacterized LOC100652914 | -1.51 | 0.019496 | 0.163689 |
| LOC145474 | Uncharacterized LOC145474 | -1.68 | 0.001993 | 0.049709 |
| LOC152742 | Uncharacterized LOC152742 | -1.79 | 0.002685 | 0.056626 |
| LOC154761 | Family with sequence similarity 115, member C pseudogene | -1.82 | 0.008837 | 0.105859 |
| LOC283140 | Uncharacterized LOC283140 | -2.21 | 0.000821 | 0.032474 |
| LOC286367 | Fp944 | -1.55 | 0.01698 | 0.151768 |
| LOC399744 | Uncharacterized LOC399744 | -1.69 | 0.048598 | 0.269653 |
| LOC400590 | Uncharacterized LOC400590 | -1.67 | 0.016657 | 0.150431 |
| LOC440894 | Uncharacterized LOC440894 | -1.99 | 0.000154 | 0.016254 |
| LOC441268 | Uncharacterized LOC441268 | -1.58 | 0.040236 | 0.243096 |
| LOC441956 | Uncharacterized LOC441956 | -2.59 | 0.002327 | 0.053094 |
| LOC645638 | WDNM1-like pseudogene | -1.93 | 0.006804 | 0.091921 |
| LOC648570 | Uncharacterized LOC648570 | -1.54 | 0.009408 | 0.109846 |
| LOC728323 | Uncharacterized LOC728323 | -1.54 | 0.001474 | 0.042395 |
| LOC728537 | Uncharacterized LOC728537 | -1.57 | 0.014089 | 0.136638 |
| LOC728802, PDE4DIP | Myomegalin-like, phosphodiesterase 4D interacting protein | -1.56 | 0.001999 | 0.049767 |
| LOC728802, PDE4DIP, ESPNP | Myomegalin-like, phosphodiesterase 4D interacting protein, espin pseudogene | -1.65 | 0.000238 | 0.01947 |
| LOC730102, SEC16B | Quinone oxidoreductase-like protein 2 pseudogene, SEC16 homolog B (S. Cerevisiae) | -1.56 | 0.01134 | 0.122218 |
| LOC730183 | Uncharacterized LOC730183 | -1.68 | 0.001273 | 0.039887 |
| LOC730755, KRTAP2-4 | Keratin associated protein 2-4-like, keratin associated protein 2-4 | -2.72 | 0.003673 | 0.067361 |
| LONRF1 | LON peptidase N-terminal domain and ring finger 1 | -1.6 | 0.007619 | 0.097973 |
| LOXL1 | Lysyl oxidase-like 1 | -1.97 | 0.000091 | 0.013715 |
| LOXL1-AS1 | LOXL1 antisense RNA 1 (non-protein coding) | -1.86 | 0.00003 | 0.00997 |
| LPAR1 | Lysophosphatidic acid receptor 1 | -1.64 | 0.000504 | 0.026157 |
| LRFN4 | Leucine rich repeat and fibronectin type III domain containing 4 | -1.56 | 0.00206 | 0.050412 |
| LRIG3 | Leucine-rich repeats and immunoglobulin-like domains 3 | -1.75 | 0.000199 | 0.018295 |
| LRP8 | Low density lipoprotein receptor-related protein 8, apolipoprotein e receptor | -1.56 | 0.000612 | 0.02868 |
| LRRC15 | Leucine rich repeat containing 15 | -2.18 | 0.00222 | 0.052102 |
| LRRC17 | Leucine rich repeat containing 17 | -2.88 | 0.000461 | 0.025329 |
| LRRC26 | Leucine rich repeat containing 26 | -1.68 | 0.002482 | 0.054715 |
| LSS | Lanosterol synthase (2,3-oxidosqualene-lanosterol cyclase) | -1.87 | 0.000009 | 0.008687 |
| LURAP1L | Leucine rich adaptor protein 1-like | -2.71 | 0.000655 | 0.029394 |
| LY6K, LOC100288181 | Lymphocyte antigen 6 complex, locus K, uncharacterized LOC100288181 | -2.02 | 0.000932 | 0.034568 |
| LYPD1 | LY6/PLAUR domain containing 1 | -2.03 | 0.00023 | 0.019468 |
| MAGI2-IT1 | MAGI2 intronic transcript 1 (non-protein coding) | -1.69 | 0.003635 | 0.067009 |
| MAMLD1 | Mastermind-like domain containing 1 | -1.54 | 0.000468 | 0.02547 |
| MANSC1 | MANSC domain containing 1 | -1.55 | 0.014029 | 0.136452 |
| MAP3K6 | Mitogen-activated protein kinase kinase kinase 6 | -1.71 | 0.000569 | 0.027718 |
| MAP3K8 | Mitogen-activated protein kinase kinase kinase 8 | -1.78 | 0.001046 | 0.03634 |
| MARCH4 | Membrane-associated ring finger (C3HC4) 4, E3 ubiquitin protein ligase | -1.62 | 0.003866 | 0.069235 |
| MBOAT1 | Membrane bound O-acyltransferase domain containing 1 | -2.76 | 0.000002 | 0.004166 |
| MCC | Mutated in colorectal cancers | -1.54 | 0.002885 | 0.059067 |
| MCM10 | Minichromosome maintenance complex component 10 | -1.77 | 0.000075 | 0.01331 |
| MCM2 | Minichromosome maintenance complex component 2 | -2.2 | 0.000036 | 0.010179 |
| MCM3 | Minichromosome maintenance complex component 3 | -1.53 | 0.000018 | 0.009534 |
| MCM4 | Minichromosome maintenance complex component 4 | -1.65 | 0.000015 | 0.009534 |
| MCM5 | Minichromosome maintenance complex component 5 | -2.29 | 0.000027 | 0.009862 |
| MCM6 | Minichromosome maintenance complex component 6 | -1.82 | 0.000005 | 0.005804 |
| MCM7 | Minichromosome maintenance complex component 7 | -1.82 | 0.000011 | 0.009308 |
| MEGF6 | Multiple EGF-like-domains 6 | -1.57 | 0.000324 | 0.022134 |
| MEST | Mesoderm specific transcript homolog (mouse) | -1.86 | 0.000006 | 0.007135 |
| METTL7B | Methyltransferase like 7B | -1.68 | 0.003978 | 0.070353 |
| MGC24103 | Uncharacterized MGC24103 | -1.58 | 0.012698 | 0.129559 |
| MGC2889 | Uncharacterized protein MGC2889 | -1.53 | 0.007582 | 0.097703 |
| MIAT | Myocardial infarction associated transcript (non-protein coding) | -1.67 | 0.012679 | 0.12946 |
| MIR17HG, MIR92A1, MIR20A, MIR19A, MIR17, MIR18A, MIR19B1 | Mir-17-92 cluster host gene (non-protein coding), microrna 92a-1, microrna 20a, microrna 19a, microrna 17, microrna 18a, microrna 19b-1 | -2.01 | 0.00592 | 0.086075 |
| MIR181A2HG | MIR181A2 host gene (non-protein coding) | -1.54 | 0.02364 | 0.18141 |
| MIR181B1 | Microrna 181b-1 | -1.98 | 0.007175 | 0.094685 |
| MIR181B2 | Microrna 181b-2 | -1.58 | 0.016376 | 0.148941 |
| MIR206 | Microrna 206 | -2.01 | 0.012674 | 0.129432 |
| MIR2355 | Microrna 2355 | -1.58 | 0.011489 | 0.123156 |
| MIR24-2, LOC284454 | Microrna 24-2, uncharacterized LOC284454 | -1.55 | 0.00515 | 0.080112 |
| MIR27B, C9orf3 | Microrna 27b, chromosome 9 open reading frame 3 | -2.05 | 0.011091 | 0.120913 |
| MIR3122 | Microrna 3122 | -2.43 | 0.0013 | 0.040042 |
| MIR3139 | Microrna 3139 | -1.68 | 0.02327 | 0.179724 |
| MIR3152 | Microrna 3152 | -3.47 | 0.000231 | 0.019468 |
| MIR3167 | Microrna 3167 | -1.7 | 0.006869 | 0.092355 |
| MIR3671 | Microrna 3671 | -3.61 | 0.000743 | 0.030888 |
| MIR3684 | Microrna 3684 | -1.52 | 0.021172 | 0.170525 |
| MIR378D2 | Microrna 378d-2 | -2.45 | 0.042042 | 0.248861 |
| MIR421 | Microrna 421 | -1.59 | 0.000267 | 0.020501 |
| MIR424, MGC16121 | Microrna 424, uncharacterized protein MGC16121 | -2.45 | 0.000227 | 0.019396 |
| MIR4451 | Microrna 4451 | -1.69 | 0.041912 | 0.248331 |
| MIR4668 | Microrna 4668 | -2.68 | 0.00013 | 0.015026 |
| MIR503 | Microrna 503 | -1.57 | 0.009119 | 0.107668 |
| MIR5047, DDX5 | Microrna 5047, DEAD (Asp-Glu-Ala-Asp) box helicase 5 | -1.76 | 0.000801 | 0.032024 |
| MIR569 | Microrna 569 | -1.64 | 0.005196 | 0.080548 |
| MIR643 | Microrna 643 | -1.65 | 0.010822 | 0.118978 |
| MIR99A | Microrna 99a | -1.71 | 0.005892 | 0.085852 |
| MIRLET7A2 | Microrna let-7a-2 | -3.81 | 0.020932 | 0.169594 |
| MIRLET7C | Microrna let-7c | -3.22 | 0.022504 | 0.176117 |
| MMP15 | Matrix metallopeptidase 15 (membrane-inserted) | -1.83 | 0.000636 | 0.029314 |
| MMP19 | Matrix metallopeptidase 19 | -1.84 | 0.000233 | 0.019468 |
| MMP2 | Matrix metallopeptidase 2 (gelatinase A, 72kda gelatinase, 72kda type IV collagenase) | -2.88 | 0.000121 | 0.014709 |
| MMP24 | Matrix metallopeptidase 24 (membrane-inserted) | -3.05 | 0.00005 | 0.01166 |
| MN1 | Meningioma (disrupted in balanced translocation) 1 | -2.47 | 0.000505 | 0.026157 |
| MND1 | Meiotic nuclear divisions 1 homolog (S. Cerevisiae) | -1.72 | 0.008062 | 0.100752 |
| MSMO1 | Methylsterol monooxygenase 1 | -1.92 | 0.000628 | 0.029058 |
| MSS51 | MSS51 mitochondrial translational activator homolog (S. Cerevisiae) | -1.77 | 0.006793 | 0.091921 |
| MTFP1 | Mitochondrial fission process 1 | -1.74 | 0.000157 | 0.016496 |
| MTRNR2L2 | MT-RNR2-like 2 | -1.6 | 0.001392 | 0.041401 |
| MTSS1 | Metastasis suppressor 1 | -1.55 | 0.016876 | 0.151339 |
| MUC1 | Mucin 1, cell surface associated | -2.78 | 0.000141 | 0.01546 |
| MVK | Mevalonate kinase | -1.6 | 0.001755 | 0.046566 |
| MVP | Major vault protein | -1.6 | 0.004267 | 0.072858 |
| MXI1 | MAX interactor 1 | -1.6 | 0.00288 | 0.059023 |
| MYB | V-myb myeloblastosis viral oncogene homolog (avian) | -2.68 | 0.000026 | 0.009862 |
| MYBL2 | V-myb myeloblastosis viral oncogene homolog (avian)-like 2 | -1.56 | 0.000191 | 0.017826 |
| MYC | V-myc myelocytomatosis viral oncogene homolog (avian) | -1.63 | 0.002577 | 0.055547 |
| MYLK | Myosin light chain kinase | -1.71 | 0.000302 | 0.02129 |
| MYLK, MYLKP1 | Myosin light chain kinase, myosin light chain kinase pseudogene 1 | -1.6 | 0.00359 | 0.066469 |
| MYPN | Myopalladin | -2.65 | 0.000215 | 0.018846 |
| NAB1 | NGFI-A binding protein 1 (EGR1 binding protein 1) | -1.61 | 0.004059 | 0.070992 |
| NAG20 | Nag20 | -2.43 | 0.000822 | 0.032474 |
| NCAM2 | Neural cell adhesion molecule 2 | -1.82 | 0.004537 | 0.075292 |
| NCKAP5 | NCK-associated protein 5 | -1.82 | 0.000415 | 0.02419 |
| NCKAP5-IT1 | NCKAP5 intronic transcript 1 (non-protein coding) | -2.06 | 0.000703 | 0.03007 |
| NCOA7 | Nuclear receptor coactivator 7 | -1.56 | 0.000181 | 0.017355 |
| NCR3LG1 | Natural killer cell cytotoxicity receptor 3 ligand 1 | -1.94 | 0.001677 | 0.045376 |
| NDRG1 | N-myc downstream regulated 1 | -1.56 | 0.00406 | 0.070992 |
| NEK6 | NIMA (never in mitosis gene a)-related kinase 6 | -1.57 | 0.000027 | 0.009862 |
| NEXN | Nexilin (F actin binding protein) | -1.62 | 0.002199 | 0.051943 |
| NEXN-AS1 | NEXN antisense RNA 1 (non-protein coding) | -1.78 | 0.011754 | 0.124409 |
| NFE2L3 | Nuclear factor (erythroid-derived 2)-like 3 | -1.64 | 0.002465 | 0.054487 |
| NIPAL2 | NIPA-like domain containing 2 | -1.56 | 0.002015 | 0.049955 |
| NNMT | Nicotinamide N-methyltransferase | -2.15 | 0.000011 | 0.009308 |
| NOG | Noggin | -2.35 | 0.00008 | 0.013636 |
| NPR3 | Natriuretic peptide receptor C/guanylate cyclase C (atrionatriuretic peptide receptor C) | -1.78 | 0.000179 | 0.017322 |
| NR1D1, THRA | Nuclear receptor subfamily 1, group D, member 1, thyroid hormone receptor, alpha | -1.69 | 0.019904 | 0.165351 |
| NR1D2 | Nuclear receptor subfamily 1, group D, member 2 | -1.87 | 0.000212 | 0.018766 |
| NR2C2AP | Nuclear receptor 2C2-associated protein | -1.68 | 0.006334 | 0.088667 |
| NR2F2 | Nuclear receptor subfamily 2, group F, member 2 | -1.52 | 0.000285 | 0.020911 |
| NREP | Neuronal regeneration related protein homolog (rat) | -2.19 | 0.000101 | 0.014213 |
| NRP1 | Neuropilin 1 | -1.73 | 0.000027 | 0.009862 |
| NSAP11 | Nervous system abundant protein 11 | -2.26 | 0.003078 | 0.061138 |
| OAS3 | 2'-5'-oligoadenylate synthetase 3, 100kda | -1.65 | 0.00051 | 0.026203 |
| OLFML2A | Olfactomedin-like 2A | -1.54 | 0.007034 | 0.093746 |
| OLR1 | Oxidized low density lipoprotein (lectin-like) receptor 1 | -2.64 | 0.000423 | 0.024333 |
| ORC1 | Origin recognition complex, subunit 1 | -1.89 | 0.000281 | 0.020804 |
| OSBPL10 | Oxysterol binding protein-like 10 | -1.78 | 0.000018 | 0.009534 |
| OSR1 | Odd-skipped related 1 (Drosophila) | -1.59 | 0.002549 | 0.055304 |
| OTUD1 | OTU domain containing 1 | -1.65 | 0.002829 | 0.058319 |
| OVOS | Ovostatin | -1.73 | 0.000926 | 0.034495 |
| OVOS, LOC100509445, OVOS2 | Ovostatin, uncharacterized LOC100509445, ovostatin 2 | -1.85 | 0.007261 | 0.095119 |
| OXCT1 | 3-oxoacid coa transferase 1 | -1.63 | 0.000123 | 0.014737 |
| PA2G4P4 | Proliferation-associated 2G4 pseudogene 4 | -1.75 | 0.005202 | 0.080604 |
| PALMD | Palmdelphin | -4 | 0.000191 | 0.017826 |
| PAPPA | Pregnancy-associated plasma protein A, pappalysin 1 | -1.73 | 0.038256 | 0.236311 |
| PAQR4 | Progestin and adipoq receptor family member IV | -2.47 | 0.000027 | 0.009862 |
| PASK | PAS domain containing serine/threonine kinase | -1.6 | 0.000838 | 0.032762 |
| PCBP4 | Poly(rc) binding protein 4 | -1.51 | 0.003939 | 0.06984 |
| PCDHB13 | Protocadherin beta 13 | -1.9 | 0.019838 | 0.165085 |
| PCDHB14 | Protocadherin beta 14 | -2.07 | 0.003385 | 0.064155 |
| PCDHB18 | Protocadherin beta 18 pseudogene | -1.58 | 0.02927 | 0.204143 |
| PCDHB2 | Protocadherin beta 2 | -1.56 | 0.002296 | 0.052916 |
| PCDHB3 | Protocadherin beta 3 | -1.72 | 0.002354 | 0.053296 |
| PCDHB5 | Protocadherin beta 5 | -2.66 | 0.000375 | 0.023543 |
| PDCD4, MIR4680 | Programmed cell death 4 (neoplastic transformation inhibitor), microrna 4680 | -1.51 | 0.001245 | 0.039387 |
| PDE1C | Phosphodiesterase 1C, calmodulin-dependent 70kda | -2.38 | 0.000053 | 0.011876 |
| PDE4DIP | Phosphodiesterase 4D interacting protein | -1.76 | 0.011317 | 0.122046 |
| PDK1 | Pyruvate dehydrogenase kinase, isozyme 1 | -1.55 | 0.000044 | 0.011216 |
| PDP1 | Pyruvate dehyrogenase phosphatase catalytic subunit 1 | -1.76 | 0.000335 | 0.022564 |
| PER3 | Period homolog 3 (Drosophila) | -2.08 | 0.003162 | 0.062024 |
| PFN1P2, LOC653513 | Profilin 1 pseudogene 2, phosphodiesterase 4D interacting protein pseudogene | -1.78 | 0.00379 | 0.068413 |
| PGAM4 | Phosphoglycerate mutase family member 4 | -1.92 | 0.028897 | 0.202746 |
| PGM5, PGM5P1, PGM5P2 | Phosphoglucomutase 5, phosphoglucomutase 5 pseudogene 1, phosphoglucomutase 5 pseudogene 2 | -2.01 | 0.000024 | 0.009862 |
| PGM5-AS1 | PGM5 antisense RNA 1 (non-protein coding) | -1.9 | 0.00299 | 0.060066 |
| PIGW | Phosphatidylinositol glycan anchor biosynthesis, class W | -1.6 | 0.002892 | 0.059094 |
| PIK3IP1 | Phosphoinositide-3-kinase interacting protein 1 | -1.69 | 0.005081 | 0.079644 |
| PLA2G7 | Phospholipase A2, group VII (platelet-activating factor acetylhydrolase, plasma) | -1.56 | 0.024089 | 0.183644 |
| PLAC4 | Placenta-specific 4 | -1.65 | 0.003874 | 0.069318 |
| PLAC8 | Placenta-specific 8 | -1.99 | 0.000795 | 0.031972 |
| PLAU | Plasminogen activator, urokinase | -1.6 | 0.000695 | 0.029882 |
| PLCB4 | Phospholipase C, beta 4 | -1.51 | 0.000018 | 0.009534 |
| PLEK2 | Pleckstrin 2 | -1.85 | 0.000133 | 0.01515 |
| PLEKHA7 | Pleckstrin homology domain containing, family A member 7 | -1.59 | 0.000527 | 0.026804 |
| PLEKHG4 | Pleckstrin homology domain containing, family G (with rhogef domain) member 4 | -2.06 | 0.000859 | 0.033267 |
| PLEKHJ1 | Pleckstrin homology domain containing, family J member 1 | -1.52 | 0.001005 | 0.035747 |
| PLEKHS1 | Pleckstrin homology domain containing, family S member 1 | -1.54 | 0.00806 | 0.100752 |
| PLK2 | Polo-like kinase 2 | -1.64 | 0.000133 | 0.01515 |
| PLXNA2 | Plexin A2 | -1.87 | 0.000661 | 0.02946 |
| PM20D2 | Peptidase M20 domain containing 2 | -1.61 | 0.001285 | 0.040023 |
| PNPLA3 | Patatin-like phospholipase domain containing 3 | -1.57 | 0.001529 | 0.042991 |
| PODXL | Podocalyxin-like | -1.53 | 0.000069 | 0.013076 |
| POLA1 | Polymerase (DNA directed), alpha 1, catalytic subunit | -1.56 | 0.000088 | 0.013711 |
| POLE2 | Polymerase (DNA directed), epsilon 2, accessory subunit | -1.81 | 0.000057 | 0.012117 |
| POLR3G | Polymerase (RNA) III (DNA directed) polypeptide G (32kd) | -1.82 | 0.029416 | 0.204695 |
| POSTN | Periostin, osteoblast specific factor | -1.98 | 0.000254 | 0.019889 |
| PPAP2B | Phosphatidic acid phosphatase type 2B | -2.1 | 0.002374 | 0.053559 |
| PPAPDC1A | Phosphatidic acid phosphatase type 2 domain containing 1A | -1.6 | 0.003086 | 0.061186 |
| PPL | Periplakin | -1.61 | 0.000942 | 0.034739 |
| PPP3CB | Protein phosphatase 3, catalytic subunit, beta isozyme | -2.03 | 0.000034 | 0.010065 |
| PRIM1 | Primase, DNA, polypeptide 1 (49kda) | -1.8 | 0.000048 | 0.011574 |
| PRKD3 | Protein kinase D3 | -1.53 | 0.000192 | 0.017845 |
| PRKG2 | Protein kinase, cgmp-dependent, type II | -1.53 | 0.012189 | 0.12703 |
| PROS1, PROSP | Protein S (alpha), protein S pseudogene (beta) | -1.56 | 0.007145 | 0.094494 |
| PRR16 | Proline rich 16 | -2.58 | 0.000082 | 0.013636 |
| PRR5L | Proline rich 5 like | -1.98 | 0.000065 | 0.012722 |
| PRSS35 | Protease, serine, 35 | -1.6 | 0.004294 | 0.072996 |
| PSG1 | Pregnancy specific beta-1-glycoprotein 1 | -1.77 | 0.038028 | 0.235578 |
| PSG10P, PSG1 | Pregnancy specific beta-1-glycoprotein 10, pseudogene, pregnancy specific beta-1-glycoprotein 1 | -2.14 | 0.005996 | 0.086485 |
| PSG11 | Pregnancy specific beta-1-glycoprotein 11 | -1.53 | 0.021803 | 0.173241 |
| PSG2 | Pregnancy specific beta-1-glycoprotein 2 | -2.07 | 0.000354 | 0.022924 |
| PSG4 | Pregnancy specific beta-1-glycoprotein 4 | -2.55 | 0.000933 | 0.034568 |
| PSG5 | Pregnancy specific beta-1-glycoprotein 5 | -3.75 | 0.00002 | 0.009546 |
| PSG6 | Pregnancy specific beta-1-glycoprotein 6 | -1.81 | 0.003023 | 0.060521 |
| PSG9 | Pregnancy specific beta-1-glycoprotein 9 | -1.77 | 0.013323 | 0.132581 |
| PTCH1 | Patched 1 | -1.59 | 0.002948 | 0.05957 |
| PTGER2 | Prostaglandin E receptor 2 (subtype EP2), 53kda | -1.54 | 0.003204 | 0.062461 |
| PTPRB | Protein tyrosine phosphatase, receptor type, B | -1.79 | 0.000268 | 0.020521 |
| PYROXD1 | Pyridine nucleotide-disulphide oxidoreductase domain 1 | -1.68 | 0.002635 | 0.056068 |
| QPCT | Glutaminyl-peptide cyclotransferase | -2.14 | 0.001158 | 0.037981 |
| RAB27B | RAB27B, member RAS oncogene family | -2 | 0.000167 | 0.016825 |
| RAB32 | RAB32, member RAS oncogene family | -1.71 | 0.000035 | 0.010065 |
| RAB7B | RAB7B, member RAS oncogene family | -1.55 | 0.029266 | 0.204143 |
| RABGAP1L-IT1 | RABGAP1L intronic transcript 1 (non-protein coding) | -1.66 | 0.020454 | 0.167639 |
| RAD51 | RAD51 homolog (S. Cerevisiae) | -1.54 | 0.000323 | 0.022134 |
| RAP1GAP2 | RAP1 gtpase activating protein 2 | -1.52 | 0.001322 | 0.040348 |
| RASEF | RAS and EF-hand domain containing | -1.97 | 0.001232 | 0.039077 |
| REEP1 | Receptor accessory protein 1 | -1.63 | 0.002031 | 0.050129 |
| RFC2 | Replication factor C (activator 1) 2, 40kda | -1.57 | 0.000016 | 0.009534 |
| RFTN1 | Raftlin, lipid raft linker 1 | -1.86 | 0.000603 | 0.028408 |
| RGMB | RGM domain family, member B | -1.82 | 0.00081 | 0.032244 |
| RGS4 | Regulator of G-protein signaling 4 | -2.14 | 0.000049 | 0.011633 |
| RGS7 | Regulator of G-protein signaling 7 | -1.79 | 0.002329 | 0.053094 |
| RHEBL1 | Ras homolog enriched in brain like 1 | -1.57 | 0.015784 | 0.145258 |
| RHOB | Ras homolog family member B | -1.81 | 0.000082 | 0.013636 |
| RHOU, DUSP5P | Ras homolog family member U, dual specificity phosphatase 5 pseudogene | -1.54 | 0.006195 | 0.087729 |
| RIPK4 | Receptor-interacting serine-threonine kinase 4 | -2.5 | 0.000151 | 0.016181 |
| RMI2 | RMI2, recq mediated genome instability 2, homolog (S. Cerevisiae) | -1.64 | 0.01439 | 0.138125 |
| RN5-8S3 | RNA, 5.8S ribosomal 3 | -1.51 | 0.007506 | 0.097021 |
| RN5S22 | RNA, 5S ribosomal 22 | -1.54 | 0.045896 | 0.260788 |
| RN5S255 | RNA, 5S ribosomal 255 | -1.62 | 0.03151 | 0.212805 |
| RN5S328 | RNA, 5S ribosomal 328 | -1.52 | 0.017995 | 0.156962 |
| RN5S385 | RNA, 5S ribosomal 385 | -2.56 | 0.000033 | 0.00997 |
| RN5S447 | RNA, 5S ribosomal 447 | -1.71 | 0.049168 | 0.2713 |
| RN5S496 | RNA, 5S ribosomal 496 | -2.87 | 0.014677 | 0.139554 |
| RNASEL | Ribonuclease L (2',5'-oligoisoadenylate synthetase-dependent) | -2.26 | 0.014085 | 0.136638 |
| RNU6-14 | RNA, U6 small nuclear 14 | -1.68 | 0.040326 | 0.243349 |
| ROR1 | Receptor tyrosine kinase-like orphan receptor 1 | -1.78 | 0.002734 | 0.057344 |
| RPL23AP7, RPL23AP82, FAM41C, MGC2752, LOC100653267, LOC100653047, LOC100289034, LOC100287195 | Ribosomal protein L23a pseudogene 7, ribosomal protein L23a pseudogene 82, family with sequence similarity 41, member C, CENPB DNA-binding domains containing 1 pseudogene, uncharacterized LOC100653267, 60S ribosomal protein l23a-like, ribosomal protein L23a pseudogene | -1.59 | 0.002627 | 0.056066 |
| RPS6KA6 | Ribosomal protein S6 kinase, 90kda, polypeptide 6 | -1.52 | 0.01669 | 0.150481 |
| RRAGC | Ras-related GTP binding C | -1.51 | 0.000728 | 0.030677 |
| RRM2 | Ribonucleotide reductase M2 | -1.58 | 0.000014 | 0.009534 |
| RUNX2 | Runt-related transcription factor 2 | -1.8 | 0.000478 | 0.025637 |
| S100PBP | S100P binding protein | -1.55 | 0.00004 | 0.010928 |
| SAMD1 | Sterile alpha motif domain containing 1 | -1.55 | 0.005148 | 0.080112 |
| SAMD12 | Sterile alpha motif domain containing 12 | -2.01 | 0.000008 | 0.008058 |
| SAMD9L | Sterile alpha motif domain containing 9-like | -1.52 | 0.00264 | 0.056068 |
| SASH1 | SAM and SH3 domain containing 1 | -1.52 | 0.013213 | 0.132198 |
| SCARB1 | Scavenger receptor class B, member 1 | -1.6 | 0.000587 | 0.028298 |
| SDAD1P1 | SDA1 domain containing 1 pseudogene 1 | -1.74 | 0.014658 | 0.139508 |
| SDC4 | Syndecan 4 | -1.63 | 3.52E-07 | 0.002695 |
| SECTM1 | Secreted and transmembrane 1 | -2.8 | 0.000025 | 0.009862 |
| SEL1L3 | Sel-1 suppressor of lin-12-like 3 (C. Elegans) | -1.63 | 0.000919 | 0.034423 |
| SEMA3C | Sema domain, immunoglobulin domain (Ig), short basic domain, secreted, (semaphorin) 3C | -1.59 | 0.000199 | 0.018295 |
| SEMA3D | Sema domain, immunoglobulin domain (Ig), short basic domain, secreted, (semaphorin) 3D | -1.65 | 0.000355 | 0.022958 |
| SERPINB2, SERPINB10 | Serpin peptidase inhibitor, clade B (ovalbumin), member 2, serpin peptidase inhibitor, clade B (ovalbumin), member 10 | -5.52 | 0.000666 | 0.029519 |
| SERPINB3 | Serpin peptidase inhibitor, clade B (ovalbumin), member 3 | -1.56 | 0.027694 | 0.197821 |
| SERPINB7 | Serpin peptidase inhibitor, clade B (ovalbumin), member 7 | -1.83 | 0.009347 | 0.109425 |
| SERPINB8 | Serpin peptidase inhibitor, clade B (ovalbumin), member 8 | -1.52 | 0.018636 | 0.160046 |
| SERPINE1 | Serpin peptidase inhibitor, clade E (nexin, plasminogen activator inhibitor type 1), member 1 | -2.33 | 0.000787 | 0.031857 |
| SETBP1 | SET binding protein 1 | -2.04 | 0.002315 | 0.052979 |
| SFN | Stratifin | -2.78 | 0.000047 | 0.011453 |
| SFR1 | SWI5-dependent recombination repair 1 | -1.51 | 0.003517 | 0.065616 |
| SFXN3 | Sideroflexin 3 | -1.65 | 0.00102 | 0.035871 |
| SH3GL3 | SH3-domain GRB2-like 3 | -1.62 | 0.001402 | 0.041468 |
| SHISA2 | Shisa homolog 2 (Xenopus laevis) | -3.78 | 0.000085 | 0.013636 |
| SIPA1L2 | Signal-induced proliferation-associated 1 like 2 | -1.7 | 0.000406 | 0.023903 |
| SKAP2 | Src kinase associated phosphoprotein 2 | -1.52 | 0.000484 | 0.025691 |
| SLC10A7 | Solute carrier family 10 (sodium/bile acid cotransporter family), member 7 | -1.92 | 0.000387 | 0.023729 |
| SLC12A6 | Solute carrier family 12 (potassium/chloride transporters), member 6 | -1.56 | 0.033889 | 0.220756 |
| SLC16A13 | Solute carrier family 16, member 13 (monocarboxylic acid transporter 13) | -1.76 | 0.0034 | 0.06438 |
| SLC16A4 | Solute carrier family 16, member 4 (monocarboxylic acid transporter 5) | -1.57 | 0.00023 | 0.019468 |
| SLC16A7 | Solute carrier family 16, member 7 (monocarboxylic acid transporter 2) | -1.68 | 0.000761 | 0.031154 |
| SLC19A3 | Solute carrier family 19, member 3 | -1.51 | 0.036003 | 0.228558 |
| SLC22A4 | Solute carrier family 22 (organic cation/ergothioneine transporter), member 4 | -1.97 | 0.000624 | 0.029015 |
| SLC25A53 | Solute carrier family 25, member 53 | -1.54 | 0.007875 | 0.099827 |
| SLC27A2 | Solute carrier family 27 (fatty acid transporter), member 2 | -2.3 | 0.000393 | 0.023729 |
| SLC38A4 | Solute carrier family 38, member 4 | -2.16 | 0.000905 | 0.034182 |
| SLC38A5 | Solute carrier family 38, member 5 | -2.09 | 0.000325 | 0.022134 |
| SLC39A10 | Solute carrier family 39 (zinc transporter), member 10 | -2.3 | 0.000007 | 0.007702 |
| SLC9A7P1 | Solute carrier family 9, subfamily A (NHE7, cation proton antiporter 7), member 7 pseudogene 1 | -1.63 | 0.002591 | 0.055744 |
| SMAD6 | SMAD family member 6 | -2.4 | 0.000309 | 0.021582 |
| SMAD7 | SMAD family member 7 | -2.51 | 0.002763 | 0.057594 |
| SMAD9 | SMAD family member 9 | -1.58 | 0.000869 | 0.033532 |
| SMPDL3A | Sphingomyelin phosphodiesterase, acid-like 3A | -1.7 | 0.000435 | 0.024665 |
| SNORA70E | Small nucleolar RNA, H/ACA box 70E | -2.05 | 0.034655 | 0.223277 |
| SNORA71B, LOC388796 | Small nucleolar RNA, H/ACA box 71B, uncharacterized LOC388796 | -1.53 | 0.005362 | 0.081806 |
| SNORD109A, SNORD109B | Small nucleolar RNA, C/D box 109A, small nucleolar RNA, C/D box 109B | -1.79 | 0.005704 | 0.084267 |
| SNORD116-28, LOC100506948, SNORD115-26, SNORD115-13, SNORD115-7, SNORD107 | Small nucleolar RNA, C/D box 116-28, uncharacterized LOC100506948, small nucleolar RNA, C/D box 115-26, small nucleolar RNA, C/D box 115-13, small nucleolar RNA, C/D box 115-7, small nucleolar RNA, C/D box 107 | -1.79 | 0.006921 | 0.092944 |
| SNORD96B | Small nucleolar RNA, C/D box 96B | -1.57 | 0.00116 | 0.038016 |
| SNRPN, LOC100506948, SNORD116-28, SNORD115-26, SNORD115-13, SNORD115-7, SNORD107 | Small nuclear ribonucleoprotein polypeptide N, uncharacterized LOC100506948, small nucleolar RNA, C/D box 116-28, small nucleolar RNA, C/D box 115-26, small nucleolar RNA, C/D box 115-13, small nucleolar RNA, C/D box 115-7, small nucleolar RNA, C/D box 107 | -2.43 | 0.000003 | 0.004251 |
| SNTB1 | Syntrophin, beta 1 (dystrophin-associated protein A1, 59kda, basic component 1) | -1.95 | 0.00102 | 0.035871 |
| SNX30 | Sorting nexin family member 30 | -1.53 | 0.000426 | 0.024429 |
| SOGA3, KIAA0408 | SOGA family member 3, KIAA0408 | -1.85 | 0.00011 | 0.014264 |
| SOLH, LOC100507047 | Small optic lobes homolog (Drosophila), uncharacterized LOC100507047 | -1.97 | 0.017798 | 0.155863 |
| SOX4 | SRY (sex determining region Y)-box 4 | -1.53 | 0.001081 | 0.036927 |
| SP100 | SP100 nuclear antigen | -1.53 | 0.000303 | 0.021315 |
| SPC24 | SPC24, NDC80 kinetochore complex component, homolog (S. Cerevisiae) | -1.79 | 0.000447 | 0.024904 |
| SPRYD3 | SPRY domain containing 3 | -1.74 | 0.002378 | 0.053627 |
| SPTLC3 | Serine palmitoyltransferase, long chain base subunit 3 | -2 | 0.00007 | 0.013149 |
| SREBF2 | Sterol regulatory element binding transcription factor 2 | -1.58 | 0.000014 | 0.009534 |
| SSBP2 | Single-stranded DNA binding protein 2 | -1.9 | 0.000384 | 0.023729 |
| ST6GALNAC5 | ST6 (alpha-N-acetyl-neuraminyl-2,3-beta-galactosyl-1,3)-N-acetylgalactosaminide alpha-2,6-sialyltransferase 5 | -1.62 | 0.004397 | 0.074044 |
| STAC | SH3 and cysteine rich domain | -1.58 | 0.008692 | 0.105086 |
| STAG1 | Stromal antigen 1 | -1.54 | 0.000916 | 0.034368 |
| STARD4 | Star-related lipid transfer (START) domain containing 4 | -1.76 | 0.000026 | 0.009862 |
| STARD4-AS1 | STARD4 antisense RNA 1 (non-protein coding) | -1.89 | 0.006158 | 0.087447 |
| STC2 | Stanniocalcin 2 | -1.75 | 0.00028 | 0.020788 |
| STEAP3 | STEAP family member 3, metalloreductase | -1.69 | 0.00003 | 0.00997 |
| STON2 | Stonin 2 | -1.83 | 0.000253 | 0.019825 |
| SYNE2 | Spectrin repeat containing, nuclear envelope 2 | -1.69 | 0.001152 | 0.037921 |
| SYNGR3 | Synaptogyrin 3 | -1.51 | 0.005094 | 0.079693 |
| SYNJ2 | Synaptojanin 2 | -1.62 | 0.000209 | 0.018655 |
| SYTL2 | Synaptotagmin-like 2 | -1.56 | 0.007596 | 0.097812 |
| SYTL4 | Synaptotagmin-like 4 | -1.56 | 0.000403 | 0.023895 |
| TACSTD2 | Tumor-associated calcium signal transducer 2 | -3.65 | 0.000054 | 0.012016 |
| TAF4B | TAF4b RNA polymerase II, TATA box binding protein (TBP)-associated factor, 105kda | -1.6 | 0.018102 | 0.157405 |
| TBC1D2 | TBC1 domain family, member 2 | -1.72 | 0.000106 | 0.014264 |
| TCF19 | Transcription factor 19 | -1.93 | 0.000034 | 0.00997 |
| TFAP4 | Transcription factor AP-4 (activating enhancer binding protein 4) | -1.7 | 0.002917 | 0.059349 |
| TGFB2 | Transforming growth factor, beta 2 | -1.75 | 0.000048 | 0.011574 |
| TGFBR1 | Transforming growth factor, beta receptor 1 | -1.51 | 0.000053 | 0.011837 |
| TGFBR3 | Transforming growth factor, beta receptor III | -1.57 | 0.001857 | 0.048125 |
| THBS2 | Thrombospondin 2 | -2.27 | 0.000026 | 0.009862 |
| THSD4 | Thrombospondin, type I, domain containing 4 | -1.86 | 0.000861 | 0.033324 |
| TIPIN | TIMELESS interacting protein | -1.64 | 0.002032 | 0.050129 |
| TK2 | Thymidine kinase 2, mitochondrial | -1.84 | 0.000104 | 0.014253 |
| TLE1 | Transducin-like enhancer of split 1 (E(sp1) homolog, Drosophila) | -1.66 | 0.001104 | 0.037191 |
| TMCO6 | Transmembrane and coiled-coil domains 6 | -1.56 | 0.000081 | 0.013636 |
| TMEM154 | Transmembrane protein 154 | -2.26 | 0.0002 | 0.018305 |
| TMEM158 | Transmembrane protein 158 (gene/pseudogene) | -2.58 | 0.001761 | 0.046614 |
| TMEM17 | Transmembrane protein 17 | -1.58 | 0.001442 | 0.042024 |
| TMEM198B | Transmembrane protein 198B, pseudogene | -1.63 | 0.00349 | 0.065356 |
| TMEM233 | Transmembrane protein 233 | -1.63 | 0.000783 | 0.031726 |
| TMEM238 | Transmembrane protein 238 | -1.61 | 0.012397 | 0.127755 |
| TMEM40 | Transmembrane protein 40 | -2.29 | 0.000272 | 0.020558 |
| TNC | Tenascin C | -1.82 | 0.000014 | 0.009534 |
| TNFAIP3 | Tumor necrosis factor, alpha-induced protein 3 | -1.68 | 0.003413 | 0.064534 |
| TNFRSF11B | Tumor necrosis factor receptor superfamily, member 11b | -1.71 | 0.048557 | 0.269569 |
| TNFRSF19 | Tumor necrosis factor receptor superfamily, member 19 | -1.57 | 0.007231 | 0.09493 |
| TNFSF10 | Tumor necrosis factor (ligand) superfamily, member 10 | -1.78 | 0.002941 | 0.059493 |
| TP73 | Tumor protein p73 | -1.59 | 0.003081 | 0.061138 |
| TRANK1 | Tetratricopeptide repeat and ankyrin repeat containing 1 | -1.71 | 0.023776 | 0.182037 |
| TRIB1 | Tribbles homolog 1 (Drosophila) | -1.54 | 0.004632 | 0.076096 |
| TRNAI6 | Transfer RNA isoleucine 6 (anticodon UAU) | -2.02 | 0.008901 | 0.106358 |
| TRNAU1AP | Trna selenocysteine 1 associated protein 1 | -1.61 | 0.002111 | 0.050981 |
| TRPC1 | Transient receptor potential cation channel, subfamily C, member 1 | -1.54 | 0.000144 | 0.015601 |
| TRPC3 | Transient receptor potential cation channel, subfamily C, member 3 | -1.68 | 0.023679 | 0.181575 |
| TSPAN18 | Tetraspanin 18 | -2 | 0.000233 | 0.019468 |
| TSPAN4 | Tetraspanin 4 | -1.55 | 0.007741 | 0.098835 |
| TTC32 | Tetratricopeptide repeat domain 32 | -1.54 | 0.005831 | 0.085317 |
| TTC9 | Tetratricopeptide repeat domain 9 | -1.51 | 0.010325 | 0.115482 |
| TTLL12 | Tubulin tyrosine ligase-like family, member 12 | -1.58 | 0.000397 | 0.023779 |
| TTLL7 | Tubulin tyrosine ligase-like family, member 7 | -1.76 | 0.00008 | 0.013636 |
| TTPA | Tocopherol (alpha) transfer protein | -1.73 | 0.000008 | 0.007769 |
| TXNIP | Thioredoxin interacting protein | -1.7 | 0.000225 | 0.019362 |
| TYMS, ENOSF1 | Thymidylate synthetase, enolase superfamily member 1 | -1.53 | 0.000142 | 0.01546 |
| UBL3 | Ubiquitin-like 3 | -1.52 | 0.003339 | 0.06376 |
| UGCG | UDP-glucose ceramide glucosyltransferase | -1.79 | 0.000001 | 0.003839 |
| UNG | Uracil-DNA glycosylase | -1.77 | 0.000128 | 0.015012 |
| UPP1 | Uridine phosphorylase 1 | -1.58 | 0.003505 | 0.065533 |
| USP43 | Ubiquitin specific peptidase 43 | -1.66 | 0.000212 | 0.018758 |
| VASH1 | Vasohibin 1 | -1.56 | 0.00565 | 0.08392 |
| VASH2 | Vasohibin 2 | -1.55 | 0.028815 | 0.202379 |
| VAT1 | Vesicle amine transport protein 1 homolog (T. Californica) | -1.54 | 0.001467 | 0.042343 |
| VCAN | Versican | -1.83 | 0.002242 | 0.052358 |
| VTRNA1-3 | Vault RNA 1-3 | -1.96 | 0.005233 | 0.080723 |
| WDR76 | WD repeat domain 76 | -1.77 | 0.00002 | 0.009546 |
| WFDC10B | WAP four-disulfide core domain 10B | -2.25 | 0.005215 | 0.080683 |
| WISP2 | WNT1 inducible signaling pathway protein 2 | -2.24 | 0.001094 | 0.037032 |
| XYLB | Xylulokinase homolog (H. Influenzae) | -1.69 | 0.005616 | 0.083661 |
| ZBED3 | Zinc finger, BED-type containing 3 | -1.75 | 0.001162 | 0.038022 |
| ZBTB20 | Zinc finger and BTB domain containing 20 | -1.67 | 0.005566 | 0.083408 |
| ZCCHC5 | Zinc finger, CCHC domain containing 5 | -1.74 | 0.002764 | 0.057594 |
| ZDHHC8P1 | Zinc finger, DHHC-type containing 8 pseudogene 1 | -1.64 | 0.00173 | 0.04616 |
| ZFP161 | Zinc finger protein 161 homolog (mouse) | -1.63 | 0.001633 | 0.04479 |
| ZFP36L2 | Zinc finger protein 36, C3H type-like 2 | -1.51 | 0.00324 | 0.062877 |
| ZHX2 | Zinc fingers and homeoboxes 2 | -1.76 | 0.000027 | 0.009862 |
| ZMIZ1 | Zinc finger, MIZ-type containing 1 | -1.86 | 0.000955 | 0.03501 |
| ZNF323 | Zinc finger protein 323 | -1.98 | 0.001706 | 0.04582 |
| ZNF367 | Zinc finger protein 367 | -1.65 | 0.000353 | 0.022896 |
| ZNF827 | Zinc finger protein 827 | -1.69 | 0.002765 | 0.057594 |
| ZNF860 | Zinc finger protein 860 | -1.86 | 0.004822 | 0.077858 |
