# Supplementary material for: Gene Expression Profile and Toxic Effects in Human Bronchial Epithelial Cells Exposed to Zearalenone
Source: PLoS One. 2014 May 2;9(5):e96404. doi: 10.1371/journal.pone.0096404 (PMC4008614; doi:10.1371/journal.pone.0096404)
Supplement: Table S4 — Summary of the enriched gene ontology (GO) terms in BEAS-2B cells after 6 h treatment with ZEA. (DOCX) [file pone.0096404.s004.docx]

Table S4. Summary of the enriched gene ontology (GO) terms in BEAS-2B cells after 6h treatment with ZEA.

| **GO Term*** | **No. of differential entities** | **Total no. of entities in the term** | **% of altered entities** | **p-values** |
| --- | --- | --- | --- | --- |
| Protein folding | 7 | 201 | 3.48 | 5.59E-07 |
| *Response to unfolded protein* | 5 | 70 | 7.14 | 7.74E-07 |
| *Response to topologically incorrect protein* | 5 | 75 | 6.67 | 1.09E-06 |
| *Response to stress* | 17 | 2033 | 0.84 | 2.97E-06 |
| *Protein refolding* | 3 | 12 | 25.00 | 3.02E-06 |
| Response to fluid shear stress | 3 | 14 | 21.43 | 4.99E-06 |
| *Negative regulation of biological process* | 17 | 2340 | 0.73 | 1.99E-05 |
| *Negative regulation of cellular process* | 16 | 2160 | 0.74 | 3.07E-05 |
| Response to organic substance | 12 | 1300 | 0.92 | 5.08E-05 |
| Regulation of apoptotic process | 10 | 928 | 1.08 | 6.84E-05 |
| Regulation of programmed cell death | 10 | 940 | 1.06 | 7.62E-05 |
| Negative regulation of apoptotic process | 7 | 427 | 1.64 | 7.64E-05 |
| Regulation of cell death | 10 | 950 | 1.05 | 8.32E-05 |
| Negative regulation of programmed cell death | 7 | 434 | 1.61 | 8.46E-05 |
| Negative regulation of cell death | 7 | 443 | 1.58 | 9.61E-05 |
| *Response to abiotic stimulus* | 8 | 612 | 1.31 | 1.09E-04 |
| Unsaturated fatty acid biosynthetic process | 3 | 38 | 7.89 | 1.11E-04 |
| Regulation of blood coagulation | 3 | 41 | 7.32 | 1.39E-04 |
| Regulation of homeostasis | 3 | 41 | 7.32 | 1.39E-04 |
| Regulation of coagulation | 3 | 45 | 6.67 | 1.84E-04 |
| Regulation of wound healing | 3 | 47 | 6.38 | 2.10E-04 |
| Response to chemical stimuli | 15 | 2297 | 0.65 | 2.52E-04 |

*The terms in italic represent enriched GO terms at both 6h and 24h.
