# Supplementary material for: Gene Expression Profile and Toxic Effects in Human Bronchial Epithelial Cells Exposed to Zearalenone
Source: PLoS One. 2014 May 2;9(5):e96404. doi: 10.1371/journal.pone.0096404 (PMC4008614; doi:10.1371/journal.pone.0096404)
Supplement: Table S5 — Summary of the enriched gene ontology (GO) terms in BEAS-2B cells after 24 h treatment with ZEA. (DOCX) [file pone.0096404.s005.docx]

Table S5. Summary of the enriched gene ontology (GO) terms in BEAS-2B cells after 24h treatment with ZEA.

| **GO Term*** | **No. of differential entities** | **Total no. of entities in the term** | **% of altered entities** | **p-values** |
| --- | --- | --- | --- | --- |
| DNA dependent DNA replication | 18 | 58 | 31.03 | 1.35E-14 |
| DNA replication initiation | 11 | 17 | 64.71 | 8.00E-14 |
| DNA replication | 29 | 193 | 15.03 | 1.44E-13 |
| Cell division | 31 | 339 | 9.14 | 8.21E-09 |
| Regulation of cell cycle | 35 | 440 | 7.95 | 3.21E-08 |
| Cell cycle phase | 36 | 464 | 7.76 | 3.84E-08 |
| Mitotic cell cycle | 33 | 406 | 8.13 | 4.81E-08 |
| Cell cycle process | 46 | 689 | 6.68 | 4.88E-08 |
| Mitosis | 24 | 242 | 9.92 | 8.96E-08 |
| Nuclear division | 24 | 242 | 9.92 | 8.96E-08 |
| Regulation of mitotic cell cycle | 20 | 178 | 11.24 | 1.41E-07 |
| Organelle fission | 24 | 252 | 9.52 | 1.92E-07 |
| Regulation of cell proliferation | 55 | 962 | 5.72 | 4.04E-07 |
| Sterol biosynthetic process | 9 | 36 | 25.00 | 4.76E-07 |
| Cholesterol biosynthetic process | 8 | 27 | 29.63 | 5.00E-07 |
| Urogenital system development | 20 | 202 | 9.90 | 1.09E-06 |
| M phase | 29 | 384 | 7.55 | 1.43E-06 |
| *Response to unfolded protein* | 11 | 70 | 15.71 | 3.65E-06 |
| DNA metabolic process | 43 | 739 | 5.82 | 5.15E-06 |
| Sterol metabolic process | 13 | 102 | 12.75 | 5.43E-06 |
| *Response to topologically incorrect protein* | 11 | 75 | 14.67 | 7.28E-06 |
| Blood vessel development | 23 | 291 | 7.90 | 8.39E-06 |
| Biosynthetic process | 96 | 2232 | 4.30 | 1.05E-05 |
| Cholesterol metabolic process | 12 | 93 | 12.90 | 1.09E-05 |
| DNA unwinding involved in replication | 5 | 12 | 41.67 | 1.14E-05 |
| *Protein refolding* | 5 | 12 | 41.67 | 1.14E-05 |
| Regulation of cell cycle process | 17 | 179 | 9.50 | 1.21E-05 |
| Vasculature development | 23 | 302 | 7.62 | 1.53E-05 |
| Metabolic process | 270 | 7989 | 3.38 | 2.02E-05 |
| Cell cycle checkpoint | 13 | 115 | 11.30 | 2.04E-05 |
| *Response to stress* | 88 | 2033 | 4.33 | 2.09E-05 |
| Cell cycle | 59 | 922 | 6.40 | 2.59E-05 |
| Regulation of phosphorylation | 37 | 639 | 5.79 | 2.65E-05 |
| Primary metabolic process | 229 | 6598 | 3.47 | 2.94E-05 |
| Regulation of protein phosphorylation | 35 | 593 | 5.90 | 2.96E-05 |
| Circulatory system development | 31 | 499 | 6.21 | 3.25E-05 |
| Chromosome segregation | 12 | 104 | 11.54 | 3.43E-05 |
| Spindle checkpoint | 5 | 15 | 33.33 | 4.03E-05 |
| Regulation of cellular component movement | 21 | 280 | 7.50 | 4.46E-05 |
| Transmembrane receptor protein serine/threonine kinase signalling pathway | 13 | 124 | 10.48 | 4.55E-05 |
| Regulation of phosphorus metabolic process | 52 | 1050 | 4.95 | 4.77E-05 |
| Cellular biosynthetic process | 88 | 2080 | 4.23 | 4.86E-05 |
| Alcohol biosynthetic process | 10 | 76 | 13.16 | 4.97E-05 |
| *Response to abiotic stimulus* | 35 | 612 | 5.72 | 5.62E-05 |
| Cell adhesion | 41 | 765 | 5.36 | 5.71E-05 |
| Biological adhesion | 41 | 768 | 5.34 | 6.23E-05 |
| Apoptotic cell clearance | 4 | 9 | 44.44 | 6.87E-05 |
| Regulation of phosphate metabolic process | 51 | 1038 | 4.91 | 6.92E-05 |
| Organic hydroxy compound biosynthetic process | 11 | 96 | 11.46 | 7.73E-05 |
| Regulation of G2/M transition of mitotic cell cycle | 5 | 17 | 29.41 | 7.93E-05 |
| Response to progesterone stimulus | 6 | 27 | 22.22 | 8.43E-05 |
| Positive regulation of cellular process | 97 | 2385 | 4.07 | 8.53E-05 |
| Cellular response to fatty acid | 3 | 4 | 75.00 | 8.57E-05 |
| Transforming growth factor beta receptor signaling pathway | 9 | 66 | 13.64 | 8.89E-05 |
| Cellular response to stress | 39 | 729 | 5.35 | 9.01E-05 |
| Response to organic substance | 60 | 1300 | 4.62 | 9.18E-05 |
| Response to oxygen level | 16 | 191 | 8.38 | 9.92E-05 |
| Organic substance metabolic process | 231 | 6784 | 3.41 | 1.05E-04 |
| Chromosome localization | 5 | 18 | 27.78 | 1.07E-04 |
| Establishment of chromosome localization | 5 | 18 | 27.78 | 1.07E-04 |
| Response to lipid | 29 | 484 | 5.99 | 1.10E-04 |
| DNA replication checkpoint | 4 | 10 | 40.00 | 1.12E-04 |
| *Negative regulation of cellular process* | 89 | 2160 | 4.12 | 1.13E-04 |
| Regulation of catalytic activity | 70 | 1600 | 4.38 | 1.20E-04 |
| Negative regulation of BMP signaling pathway | 6 | 29 | 20.69 | 1.29E-04 |
| Negative regulation of phosphorus metabolic process | 17 | 218 | 7.80 | 1.46E-04 |
| Alcohol metabolic process | 17 | 218 | 7.80 | 1.46E-04 |
| Cellular macromolecule biosynthetic process | 61 | 1354 | 4.51 | 1.56E-04 |
| DNA duplex unwinding | 8 | 56 | 14.29 | 1.57E-04 |
| Cell proliferation | 29 | 495 | 5.86 | 1.61E-04 |
| Regulation of protein modification process | 39 | 752 | 5.19 | 1.72E-04 |
| Negative regulation of G2/M transition of mitotic cell cycle | 4 | 11 | 36.36 | 1.72E-04 |
| Regulation of cellular component organization | 41 | 805 | 5.09 | 1.72E-04 |
| Response to decreased oxygen levels | 15 | 180 | 8.33 | 1.72E-04 |
| *Negative regulation of biological process* | 94 | 2340 | 4.02 | 1.76E-04 |
| DNA geometric change | 8 | 57 | 14.04 | 1.78E-04 |
| Macromolecule biosynthetic process | 62 | 1390 | 4.46 | 1.80E-04 |
| M-phase of mitotic cell cycle | 25 | 249 | 10.04 | 1.89E-04 |
| Response to transforming growth factor beta stimulus | 9 | 73 | 12.33 | 1.96E-04 |
| Cellular response to transforming growth factor beta stimulus | 9 | 73 | 12.33 | 1.96E-04 |
| Cellular macromolecule metabolic process | 154 | 4262 | 3.61 | 2.18E-04 |
| Organic cyclic compound biosynthetic process | 47 | 982 | 4.79 | 2.45E-04 |
| Mitotic spindle checkpoint | 4 | 12 | 33.33 | 2.52E-04 |
| Cellular metabolic process | 215 | 6328 | 3.40 | 2.72E-04 |

*The terms in italic represent enriched GO terms at both 6h and 24h.
