# Supplementary material for: Gene Expression Profile and Toxic Effects in Human Bronchial Epithelial Cells Exposed to Zearalenone
Source: PLoS One. 2014 May 2;9(5):e96404. doi: 10.1371/journal.pone.0096404 (PMC4008614; doi:10.1371/journal.pone.0096404)
Supplement: Table S6 — Pathways enriched in BEAS-2B cells after 24 h treatment with ZEA. (DOC) [file pone.0096404.s006.doc]

Table S6. Pathways enriched in BEAS-2B cells after 24h treatment with ZEA.

| **Pathways** | **No. of genes in pathway** | **ES*** | **NES*** | **FDR*** |
| --- | --- | --- | --- | --- |
| RUIZ_TNC_TARGETS_DN | 141 | -0.70891 | -2.5935 | 0 |
| SENESE_HDAC1_AND_HDAC2_TARGETS_DN | 221 | -0.65084 | -2.49679 | 0 |
| STEIN_ESRRA_TARGETS_RESPONSIVE_TO_ESTROGEN_DN | 41 | -0.79602 | -2.39452 | 0 |
| REACTOME_SYNTHESIS_OF_DNA | 90 | -0.69222 | -2.40764 | 0 |

*ES: Enrichment; NES: normalized enrichment; FDR: false discovery rate
